# MuSpAn: a toolbox for multiscale spatial analysis

**DOI:** 10.1038/s41467-026-75649-7

**Published:** 2026-08-13

**Authors:** Joshua A. Bull, Joshua W. Moore, Shania M. Corry, Muyang Lin, Hayley L. Belnoue-Davis, Eoghan J. Mulholland-Illingworth, Simon J. Leedham, Helen M. Byrne

**Affiliations:** 1 Wolfson Centre for Mathematical Biology, Mathematical Institute, University of Oxford, Oxford, UK; 2 Centre for Human Genetics, University of Oxford, Oxford, UK; 3 Ludwig Institute for Cancer Research, University of Oxford, Oxford, UK

**Keywords:** Software, Data processing, Statistical methods, Software, Cancer

## Abstract

Advances in multiplex imaging and spatial omics have revolutionised spatial data generation in biology, revealing complex tissue organisation across multiple scales. However, methods for analysing these data have lagged behind, with fragmented, study-specific pipelines and limited guidance for tool selection. To address this, we introduce MuSpAn, a Multiscale Spatial Analysis package offering intuitive, flexible access to a wide range of mathematical tools - including spatial statistics, topological data analysis, geometry, and networks - within a unified framework. MuSpAn supports efficient data querying, is agnostic to imaging modality, and provides extensive documentation and community support. It enables users to create custom pipelines or conduct unbiased exploratory analyses. We demonstrate MuSpAn’s capacity to interrogate cross-compartmental cell interactions at multiple length scales in both normal and neoplastic tissue using mouse intestinal spatial transcriptomic datasets. Applied to a CMS4-like murine intestinal cancer model, MuSpAn identifies a continuum of fibroblastic functional phenotypes associated with discrete and coordinated fibroblast-immune interactions, highlighting its utility as a discovery tool across diverse biological contexts.

## Abstract (web summary)

Bull, Moore, and colleagues present MuSpAn, a flexible platform for analysing spatial biology data across scales. It integrates diverse methods to enable quantitative characterisation of tissue organisation and reveals coordinated fibroblast-immune interactions in colorectal cancer.

## Introduction

Coordinated interactions between diverse cell types define tissue architecture and aberrant disruption of these interactions is recognisably pathognomonic in disease. Advances in imaging technologies now enable these spatial associations to be mapped at unprecedented resolution, producing spatial datasets that span biological scales from subcellular features to entire organs^1–6^. Methods such as fluorescence in situ hybridisation-based imaging^7,8^, spatial transcriptomics^9^ and spatial proteomics^10–13^ generate rich, multiplexed views of tissue composition, yet this richness creates analytical bottlenecks that limit biological discovery.

Considerable progress has been made in addressing technical challenges such as storage, visualisation, segmentation and phenotyping, often with the help of Artificial Intelligence-based (AI) tools^14–21^. Tools such as DeepCell^22^, StarDist^23^ and CellPose^24^ provide increasingly robust cell segmentation, while packages including ScanPy^25^ and Seurat^26^ work with spatial data to assign phenotypes to distinct cells. However, after applying these tools a central challenge remains: extracting meaningful biological insight from spatially complex data. Averaged metrics such as cell counts or densities obscure patterns of cellular positioning that often hold the key to biological function. For example, the prognostic significance of immune cells in cancer differs dramatically depending on whether they cluster at the tumour margin or infiltrate the lesion^27^.

Crucially, cell signalling influences both cellular phenotypes and tissue organisation, and this ranges from local autocrine and paracrine pathways through to long range communication (e.g. the endocrine system and extracellular vesicles)^28^. Resultant cell interactions occur across multiple spatial scales: from local, proximity-based interactions (e.g. antigen presentation) to formation of higher-order multicellular structures (e.g. tertiary lymphoid aggregates, intestinal crypts, hepatic lobules). Analyses focussed on a single scale risk overlooking essential biology, underscoring the need for multiscale methods that relate spatial patterns to underlying processes^29^.

Most existing software tools focus on specific imaging modalities or biological questions, typically implemented as fixed, sequential pipelines^30–36^. For instance, Schürch et al.^30^ used sliding windows to characterise neighbourhoods; SpottedPy applies Getis-Ord hotspot analysis to Visium data^31^; and SPIAT employs cross-K functions and clustering^32^. While powerful in their proposed contexts, it can be difficult to compare methods and select appropriate tools due to differences in programming language, imaging modality and chosen analytical approaches. Few platforms support systematic comparison and integration of diverse methods; notably SquidPy provides a range of graph-based approaches with efficient data structures^37,38^ and Giotto Suite^39^ includes an end-to-end ecosystem containing several spatial analysis methods. However, opportunities to combine complementary mathematical approaches remain underexploited, despite improving feature detection^29^. In response to this, we present an innovative platform, MuSpAn, which extends the work of previous toolboxes by exploiting the generic mathematical structures underlying spatial data to empower the user to conduct generic spatial analyses on cells, cell-derived objects such as neighbourhoods, or indeed any spatial objects including manual annotations, tissue boundaries, transcripts and more.

To address these challenges, we introduce **MuSpAn**, a Python library for **Mu**ltiscale **Sp**atial **An**alysis. MuSpAn unifies more than 100 methods for quantifying spatial relationships across biological scales. Researchers can interactively compare analytical strategies tailored to their own questions, from cell-cell contacts to tissue geometry and topology. Critically, MuSpAn enables cross-scale integration, allowing insights at one level (e.g. subcellular expression) to inform analyses at another (e.g. tissue microenvironment). Its modular design supports incorporation of new methods, is underpinned by robust testing, and is accompanied by detailed tutorials, comprehensive documentation and an active online user-developer community. Here, we demonstrate the capability of MuSpAn to interrogate cross-cell compartment spatial associations across length scales in normal and neoplastic mouse intestine. Together, these approaches illustrate how MuSpAn detects hierarchical organisation across scales - from single-cell positioning to tissue compartmentalisation - enabling applications such as immune niche mapping and structural feature discovery.

## Results

### MuSpAn provides a framework for quantitative spatial analysis at multiple scales

**Fig. 1:**
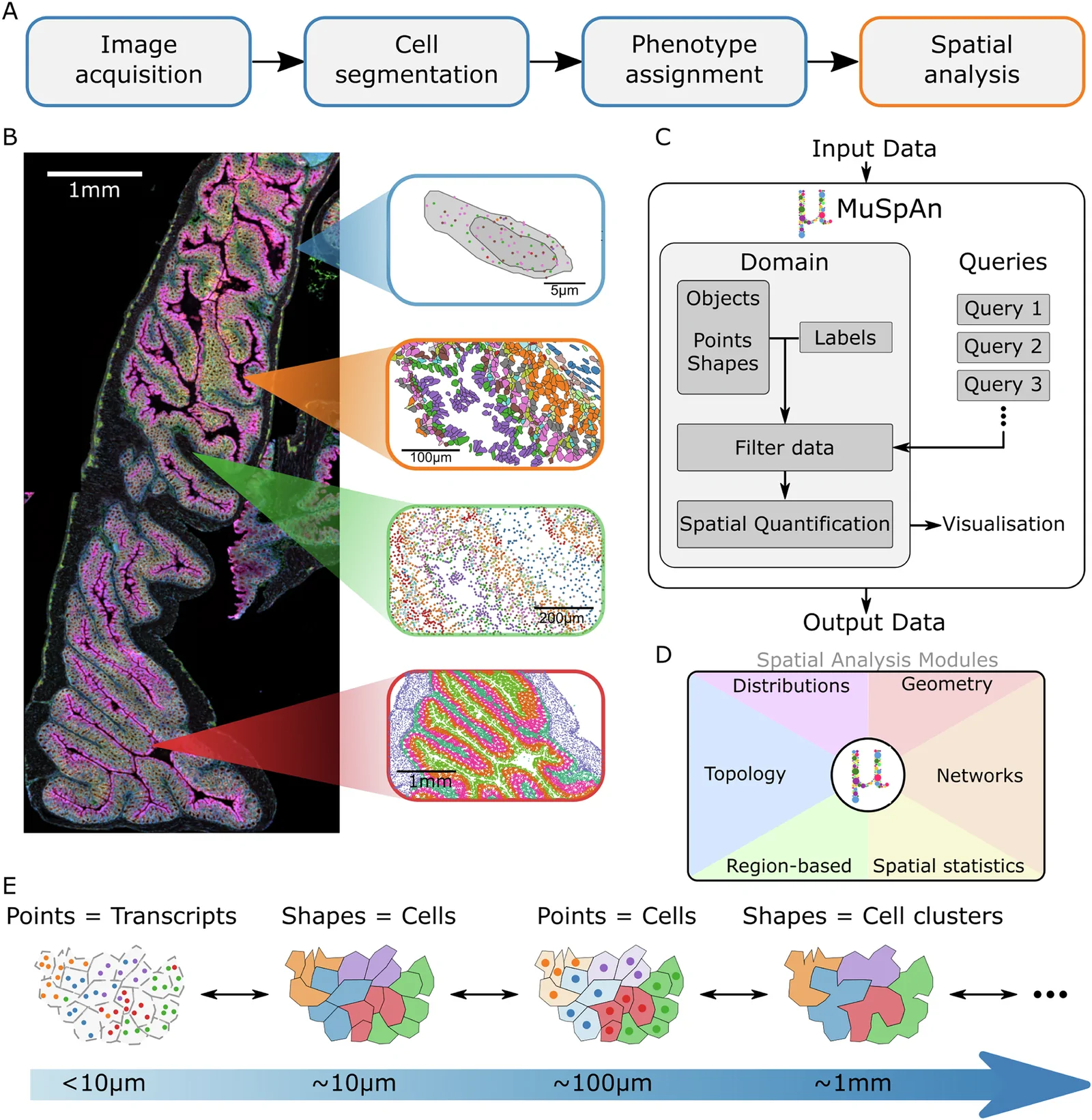
Overview of MuSpAn. MuSpAn conducts quantitative spatial analysis, the end point of typical workflows for processing spatial biology (**A**). MuSpAn handles spatial structure across length scales, illustrated here using healthy mouse colon tissue from the Xenium platform (**B**). Internally, MuSpAn holds geometric information within a single ‘domain’, which the user can interact with via ‘queries’ (**C**), and by selecting from spatial quantification methods which are organised into six modules (**D**). MuSpAn represents data as geometric objects (**E**), ensuring analytical methods can be applied at any length scale, provided the objects are structurally compatible.

MuSpAn is a Python package for spatial analysis, which we here take to encompass any quantitative metric designed to describe the spatial structure of biological data, and which is applicable following segmentation and phenotype assignment (Fig. 1A). It accepts spatial coordinates (e.g. centroids, boundaries) and metadata (e.g. cell types, marker intensities, morphometrics), and is compatible with outputs from any generic segmentation and phenotyping pipelines. Additionally, MuSpAn provides helper functions to facilitate data import from frameworks including SpatialData^19,38^, QuPath^14^ and Xenium Explorer (10x), or from the segmentation masks produced by tools such as CellPose^24^, StarDist^23^ and CellSAM^22^. The package is supported by extensive documentation and tutorials (https://www.muspan.co.uk) and rigorous unit testing.

The key design principle underlying MuSpAn is that quantitative descriptions of spatial structure can be applied to any geometric objects, regardless of their biological interpretation. Intuitively, we understand that a polygon has an area which can be measured, regardless of whether the polygon represents the computationally detected boundary of a cell, a manually drawn annotation, or the boundary of a necrotic region of tissue on a digital pathology slide. MuSpAn provides a vast library of quantitative descriptive tools for working with arbitrary geometric data, based on mathematical methods developed in fields as diverse as astronomy, ecology and statistics. In spatial biology, we can derive second-order objects from primary objects of biological interest: a collection of transcripts in Xenium data can be viewed as belonging to a single cell, clusters of cells with similar profiles may be treated as forming a single emergent tissue unit (e.g. a colorectal crypt), and these can in turn be viewed as geometric objects that form part of a larger emergent structure. By providing flexible tools to interrogate the spatial arrangement of these structures that divorce their geometric interpretation from their biological one, the key strength of MuSpAn is its capacity to move easily between different biological spatial scales, and to chain together complex spatial analyses of derived objects into flexible computational pipelines.

MuSpAn enables flexible and interactive analysis across length scales. For example, spatial transcriptomics data may describe subcellular transcript localisation (<10 μm), cell-cell contact (≈100 μm), or tissue structures (≥1000 μm) (Fig. 1B). Rather than focus on a fixed length scale, MuSpAn represents spatial data as geometric objects (points or shapes) which could reflect diverse spatial features (e.g. individual transcripts, cell centres, cell boundaries, user-defined annotations; Fig. 1C). Objects and associated metadata labels are stored within a single domain, which users may query to specify subsets of objects via spatial cues (e.g. ‘points inside a hypoxic region’, ‘cell boundaries within 50 μm of the tumour invasive margin’), labels (e.g. ‘macrophages’, ‘cells highly expressing PD-L1’, ‘cellular neighbourhoods enriched with T cells’), or other object properties. Over 100 analysis methods are available to interrogate objects within a given region of interest (ROI, shape object), described in detail in the Supplementary Information. Collections of shapes can be used to interactively restrict analyses to a specific set of ROIs such as manual annotations or tissue compartments. Within these regions, boundary corrections and updates to null models are calculated automatically to ensure spatial quantification methods remain statistically valid. These methods are organised into six modules (Fig. 1D), and each can be applied to objects at any length scale. Complex objects can be simplified (e.g. shapes to centroids) to facilitate analyses at different scales, propagating embedded metadata and reducing computational cost (Fig. 1E).

Below, we apply MuSpAn to Xenium datasets of healthy mouse colon and a consensus molecular subtype 4 (CMS4)-like murine colorectal cancer model. The first example showcases MuSpAn’s ability to identify structural features across multiple scales in a publicly available tissue, while the second shows how it can be used to identify new hallmarks of disease using a bespoke Xenium panel and targeted analysis.

### Validation of MuSpAn in a healthy mouse colon exemplar

We demonstrate MuSpAn’s capacity for exploratory data analysis across length scales using an ‘off-the-shelf’ dataset of healthy mouse colon from the Xenium platform (10x). The gene panel and cell annotations represent a generic subset of cells whose behaviour is well characterised in the healthy colon. We show how some of the quantitative tools within MuSpAn can identify this expected cell behaviour, validating their use for spatial data analysis and showcasing some of the features outlined in Fig. 1. These include analysis of data across different length scales, calculating statistics within irregularly shaped regions, and the use of multiple complementary methods to support the same biological conclusions. First, we consider cell-scale geometry and subcellular transcript localisation, then we progress up the spatial scales to characterise the whole tissue section. The length scales considered are selected to showcase MuSpAn’s ability to analyse biological data across spatial scales; any spatial method can be applied to data at any biological scale, and lengthscale parameters should be motivated by biological, rather than mathematical, considerations. Where appropriate, we explain briefly why specific length scales were chosen for these examples.

Many of the descriptive statistics in these examples use significance testing to identify non-random spatial structure. MuSpAn uses tests against null hypotheses of complete spatial randomness to highlight data which exhibits non-random spatial structure; full details of the null models used in this section can be found in the Supplementary Information (Supplementary Note 5).

### Cell geometry and subcellular transcript localisation

**Fig. 2:**
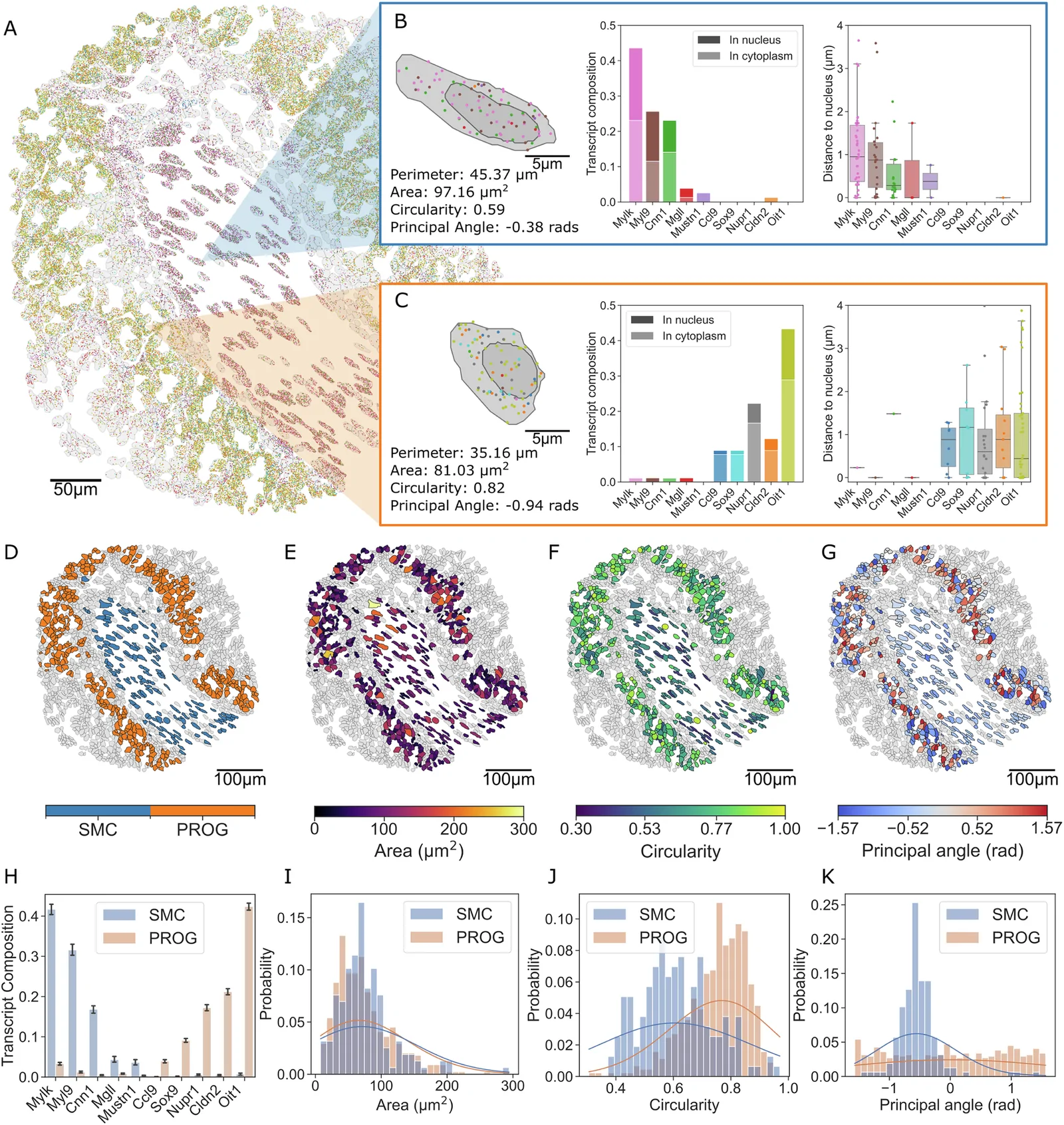
Quantification at the cell scale. Cell scale analysis on a ROI from a healthy mouse colon (**A**). Morphological metrics and transcript summary statistics are shown for a representative Smooth Muscle Cell (SMC; **B**) and Epithelial Progenitor cell (PROG; **C**). Boxplots show the median (centre line), 25th and 75th percentiles (box), and minima/maxima (whiskers). (**B**: Mylk, *n* = 37; Myl9, *n* = 22; Cnn1, *n* = 18; Mgll, *n* = 3; Mustn, *n* = 2; Cldn2, *n* = 1; all others *n* = 0. C: Mylk, *n* = 1; Myl9, *n* = 1; Cnn1, *n* = 1; Mgll, *n* = 1; Mustn, *n* = 0; Ccl9, *n* = 8; Sox9, *n* = 8; Nupr1, *n* = 20; Cldn2, *n* = 11; Oit1, *n* = 41.). Cell summary labels are visualised as heatmaps colouring the cell boundaries for SMCs and PROG cells (**D**: Celltype. **E**: Area. **F**: Circularity. **G**: Principal angle.). Summary information to compare SMCs (*n* = 158) and PROG cells (*n* = 444) can also be viewed quantitatively, such as the proportion of selected transcripts in each cell (**H**; mean, 95% CI), or distributions of morphological metrics (metric, Wilcoxon rank-sum test two-sided *p*-value; **I**: area, *p* = 0.26. **J**: circularity, *p* = 1.89 × 10^−31^. **K**: principal angle, *p* = 1.47 × 10^−31^). Solid lines show Gaussian KDEs of continuous distributions.

MuSpAn can analyse transcriptional polarisation and morphometrics at subcellular resolution, through characterisation of the spatial localisation of transcripts associated with individual Smooth Muscle Cells (SMCs) and Epithelial Progenitor cells (PROG) in the normal mouse colon (Fig. 2). Within a small ROI (0.13mm^2^), we identified five transcripts preferentially expressed by PROG cells (*Ccl9, Sox9, Nupr1, Cldn2, Oit1*) and five preferentially expressed by SMCs (*Mylk, Myl9, Cnn1, Mgll, Mustn1*), using the segmentation mask to define the shapes of individual cell boundaries and nuclei for analysis. For representative cells, we quantify both cell geometry and transcript localisation within the nucleus and cytoplasm (Fig. 2B, C). For each metric, MuSpAn facilitates visualisation across these cell subtypes in the ROI (Fig. 2D–G) and quantification of differences in its distribution between SMC and PROG populations (Fig. 2H–K). The cell types have comparable areas, but we find significant differences in cell circularity and principal angle consistent with the tissue orientation of these different individual cell types. These results show that by interpreting individual transcripts as points MuSpAn can quantify subcellular transcript distributions and relate them to individual cell geometric features such as morphology, polarisation and tissue orientation.

### Quantification of cell proximity and interaction networks

**Fig. 3:**
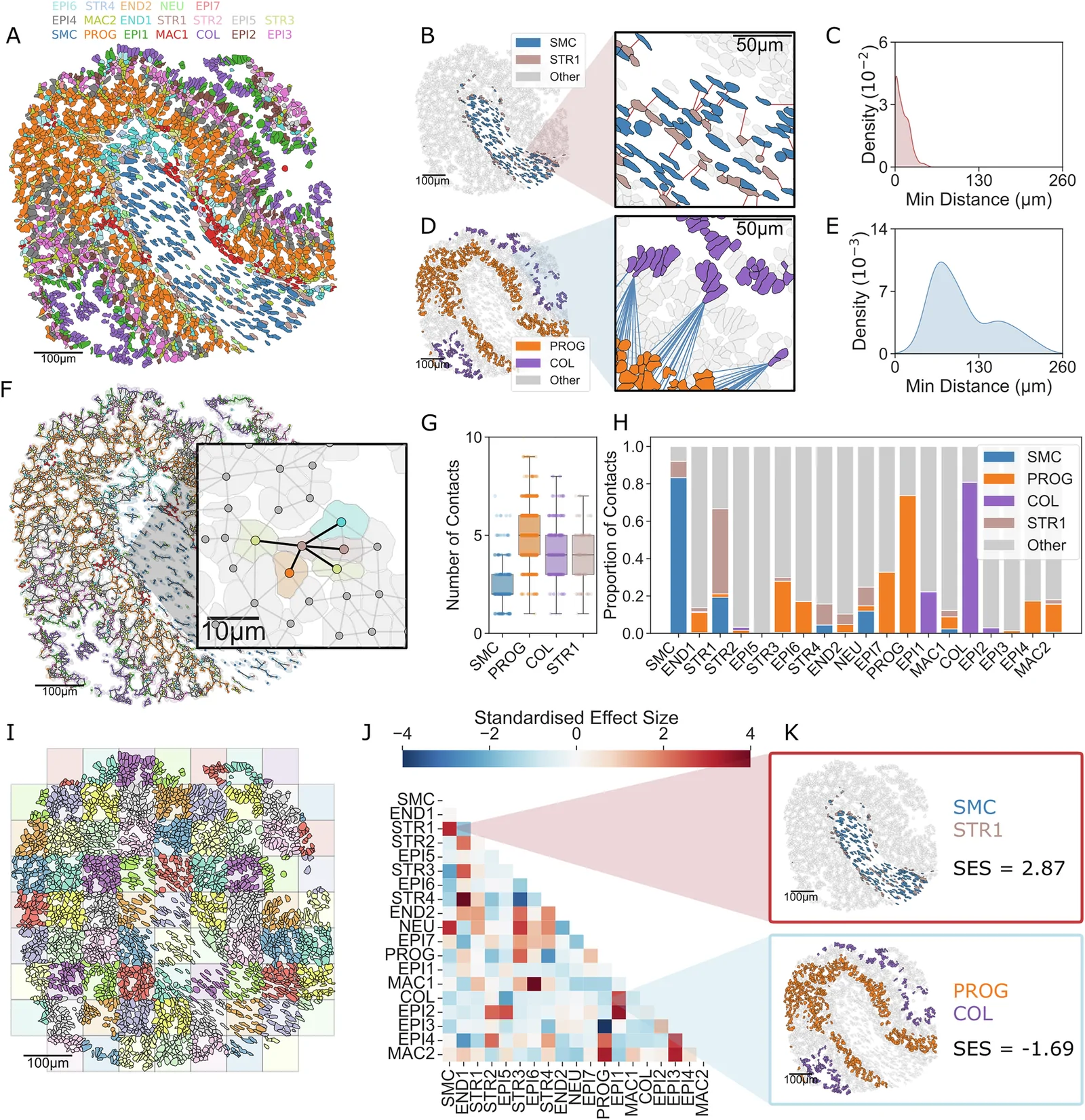
Quantification of cell-cell proximity. Cell boundaries coloured by cell type (**A**), exemplifying how cell-cell proximity can be measured using the nearest neighbour distances for individual cells (**B**: SMC-STR1; **D**: PROG-COL) and their distributions (**C**: SMC-STR1, **E**: PROG-COL). This ROI could alternatively be treated as a proximity network (**F**), with nodes placed at cell centroids and edges present when cell boundaries are within 1 μm. Selected cell types can then be compared by degree distribution (**G**; SMC, *n* = 203; PROG, *n* = 728; COL, *n* = 267; STR1, *n* = 91; boxplots show the median (centre line), 25th and 75th percentiles (box) and minima/maxima (whiskers)) or the mean proportion of neighbours of different types (**H**). Alternatively, tiling the ROI into 75 μm × 75 μm quadrats (**I**) permits comparison using the quadrat correlation matrix (**J**; QCM), which identifies colocalisation (**K**; SMC-STR1, *z* = 2.87) and exclusion (**K**; PROG-COL, *z* = −1.69).

Next, we used three complementary methods within MuSpAn to examine proximity based cell interaction networks within a larger ROI (≈ 0.38 mm^2^, Fig. 3A) in the normal mouse colonic mucosa. First, nearest-neighbour distances revealed strong (non random) proximity for SMCs and Type 1 Stromal cells (STR1) (10.9 μm, Fig. 3B, C) within the intestinal lamina propria with contrasting exclusion for PROG cells to Colonocytes (COLs) (106.0 μm, Fig. 3D, E) which were distributed differently along the epithelial vertical axis. *z*-scores from the Average Nearest Neighbour Index^32,40^ confirmed significance (*p* < 10^−3^). Secondly, contact networks (Fig. 3F) showed that SMCs were relatively isolated (low degree, Fig. 3G) but frequently connected to STR1s, consistent with the anticipated presence of these cell types in the lamina propria (Fig. 3H). Lastly, quadrat correlation analysis confirmed positive correlation for SMC-STR1 and exclusion for PROG-COL (Fig. 3I–K), and also identified other, non-random, positive and negative cell associations. Quadrats of size 75 μm × 75 μm were chosen to ensure that most quadrats would contain around 20–30 cells. Together, these data highlight MuSpAn’s ability to integrate multiple analytical methods to assess proximity based cell correlations. The convergence of multiple mathematical techniques in identifying the same cell relationships provides reassurance of biological relevance^29^. These tools can be used to rapidly identify significant cell correlations across heterogenous cell types within a tissue ecosystem. Applied in this way, MuSpAn acts as a hypothesis generating discovery tool to quantify local tissue cellular organisation and to begin to decipher the signalling networks that underpin key identified proximity-based cell associations.

### Assessment of multiscale spatial patterns

**Fig. 4:**
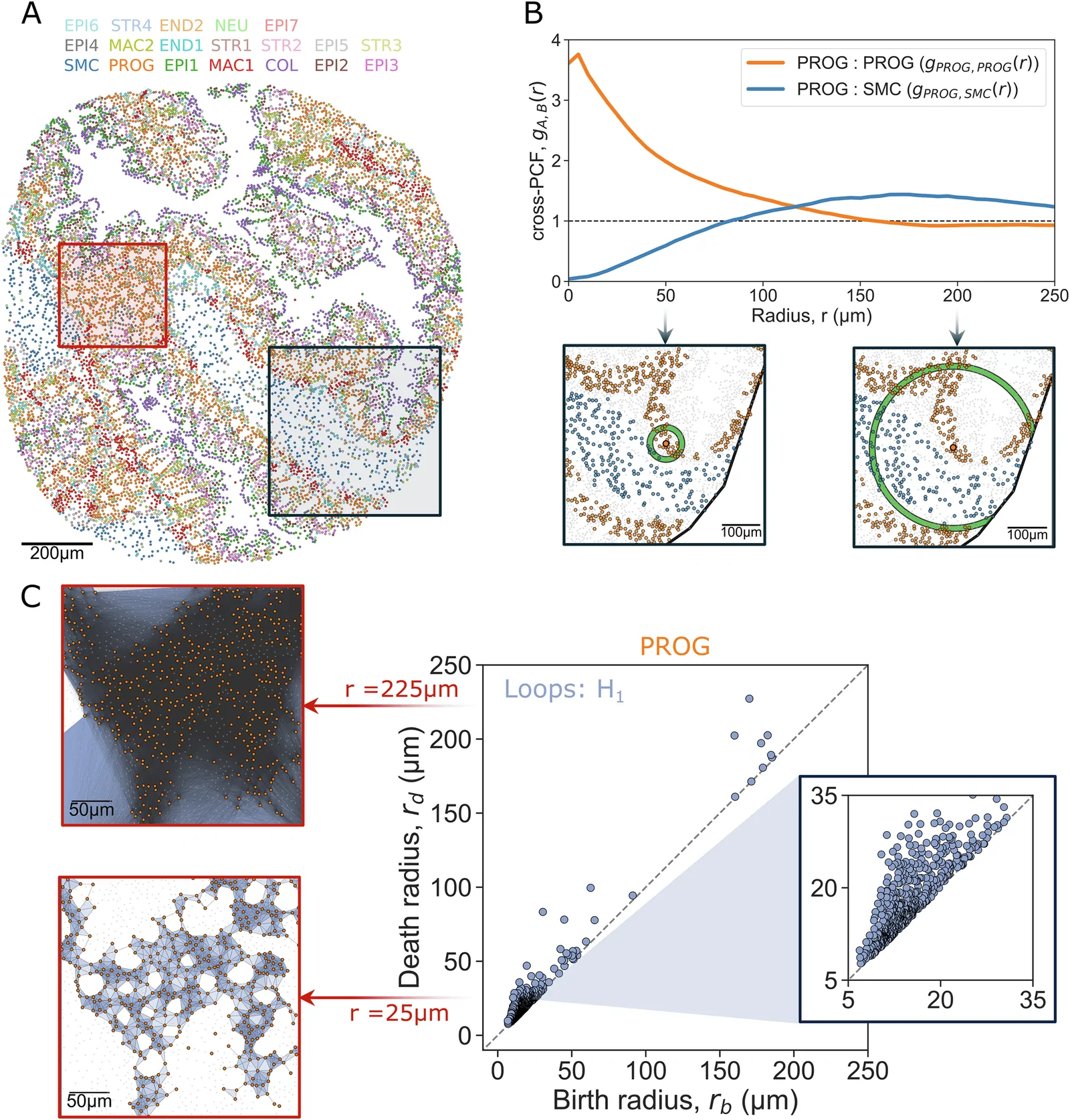
Quantification of cell-cell relationships across length scales. Cell-cell relationships across length scales are exmplified using an ROI with cell centres coloured by cell type (**A**). The cross-PCFs (**B**) for PROG-SMC (blue curve) and PROG-PROG (orange curve) show exclusion and colocalisation, respectively. The annuli of radii 50 μm and 200 μm show the stages in calculating the cross-PCF, with automatic boundary correction (insets **B**, corresponding to the black box in **A**). The persistence diagram of the VR filtration (**C**) identifies loops (*H*_1_, purple), with simplicial complexes at 25 μm and 225 μm showing stages involved in constructing the VR filtration on PROG cells (red box in **A**).

Having identified some proximity based cell colocalisation and exclusion, we next used two complementary techniques to assess the distribution and topology of epithelial (PROG) and smooth muscle (SMC) cell types. We consider a larger ROI (≈1.13 mm^2^, Fig. 4A) showing the spatial position of cell centroids within a section of normal colonic tissue architecture. The cross pair correlation function (cross-PCF) identified short to medium range clustering of PROG cells (Fig. 4B, *g* > 1 for *r* < 150 μm) but exclusion of PROG-SMCs (Fig. 4B, *g* < 1 for *r* < 80 μm), consistent with the discrete compartmentalisation of the epithelium and sub-crypt lamina propria. The cross-PCF was calculated for *r* < 250 μm (approximately the width of the epithelium), using annuli of width 15 μm (approximately one cell diameter). MuSpAn automatically corrected for boundary effects involving points located close to the boundary of the ROI (Fig. 4B insets). These results show how the cross-PCF can quantify both the strength and length scale of positive and negative cell associations. We also used persistent homology (PH), a form of topological data analysis (TDA), to identify loop-like (‘*H*_1_’) features in the distribution of PROG cells with an approximate radius of ≈25 μm, corresponding to transected colonic crypts (Fig. 4C). PH captures birth and death radii of these features, consistent with intercellular spacing and crypt diameters, respectively. This analysis demonstrates the ability of PH to identify and quantify the organisation of multiple cell types into higher-order tissue structural features such as crypts, alveoli, or hepatic lobules which may be difficult to discern with conventional correlation analyses. The loop features may define normal tissue architecture which can be lost during disease progression, as seen in inflammation or neoplasia.

### Resolving tissue-scale organisation

**Fig. 5:**
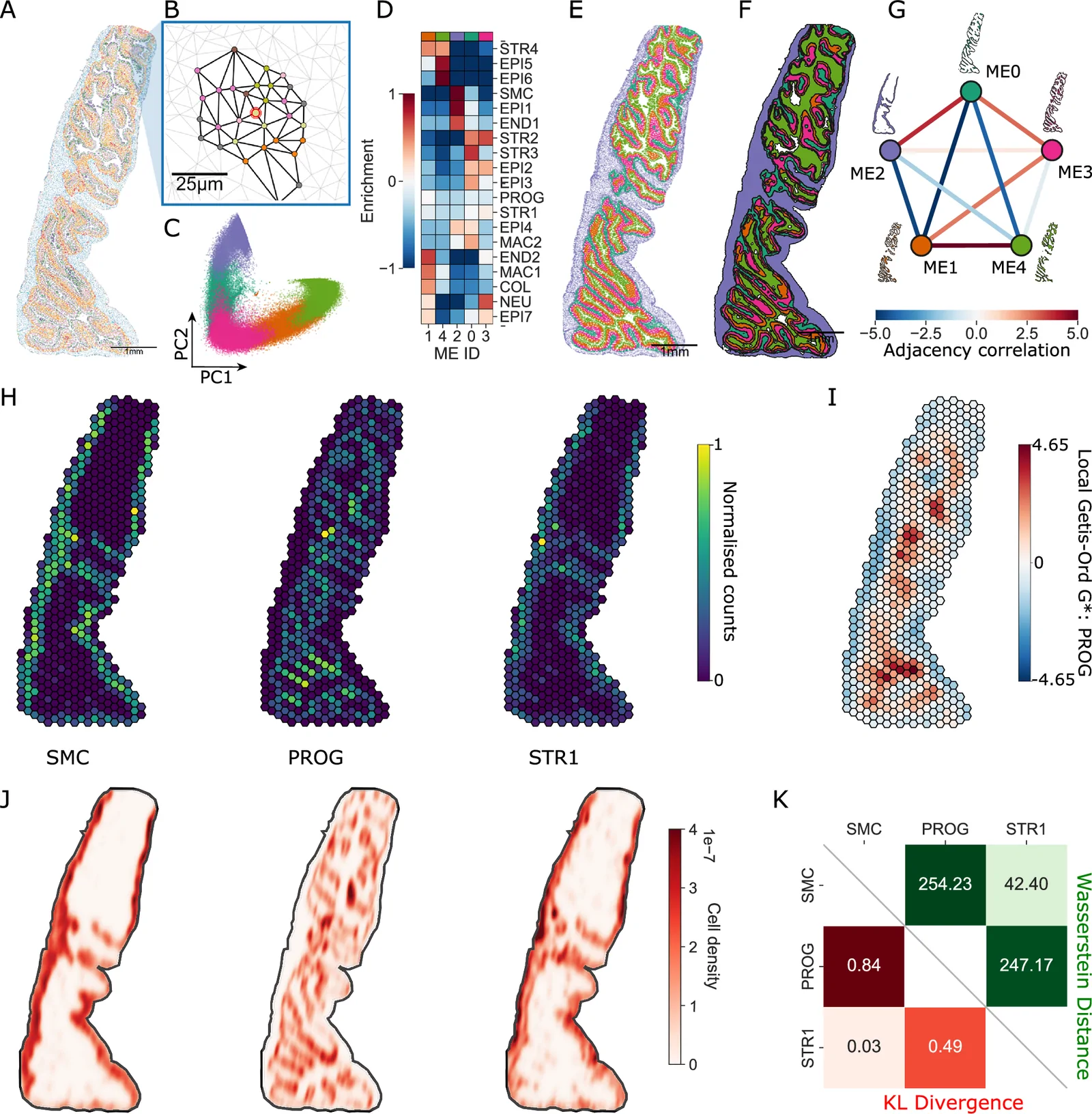
Quantification of tissue scale features. At tissue scale, we demonstrate microenvironment analysis (**A**–**G**), region-based analysis (**H**, **I**) and distribution-based analysis (**J**, **K**), using a large ROI from the healthy mouse dataset (**A**, cell centres coloured by cell type). Microenvironment analysis uses 3-hop neighbourhoods of a Delaunay Triangulation of the cells (**B**; centred on the red-outlined cell). 3-hop neighbourhood compositions are visualised with PCA (**C**), with cells assigned microenvironment (ME) IDs via *k*-means clustering (*k* = 5). Each microenvironment has distinct cell type compositions (**D**), and with spatial distributions observed by colouring cell centres by ME ID (**E**). Spatially contiguous cell centres with the same ME ID are grouped into shapes (**F**), which can be used for adjacency correlation of shapes with different ME IDs (**G**). Region-based analysis can be conducted by grouping cells into a hexagonal lattice (**H**, 100 μm sides, coloured by cell counts of selected cell types), and hotspots identified using Getis-Ord^*^ analysis (**I**, PROG cells). Alternatively, kernel density estimates (KDEs) show the distribution of selected cell types (**J**), which can be compared using KL-divergence (**K**, red, lower triangle) or Wasserstein distance (**K**, green, upper triangle).

We used three complementary methods to characterise the tissue organisational structures within the normal mouse colon at the level of the whole section (Fig. 5A). We used Delaunay graphs (≤30 μm, approximating a cell-cell contact network inferred from cell centres) to define cellular neighbourhoods; assigning each cell centre to one of five distinct microenvironments (MEs) according to the composition of its 3-hop neighbourhood (Fig. 5B–E)^30,41,42^. We extended this ‘cellular neighbourhood’ analysis by converting contiguous regions of cells assigned to the same ME into shapes. This enabled us to conduct an adjacency permutation test (APT) on the derived cell neighbourhood objects which revealed ordered transitions from the serosal wall through to the epithelial cell compartment (Fig. 5E–G). A similar analysis performed directly on the ME labelled cell objects (Fig. 5E) would lead to misleading *z*-scores strongly affected by cell densities along the borders of the regions.

Secondly, we compartmentalised the ROI into a hexagonal lattice of side length 100 μm (reflecting the approximate width of the tissue structures observed above). Cell counts within each hexagon (Fig. 5H) highlighted peripheral enrichment of muscularis cell types SMC and STR1 and central hotspots of PROG, detected by Local Getis-Ord^*^ (Fig. 5I).

Lastly, we use kernel density estimation (KDE) to generate smooth maps of distributions (Fig. 5J). KL divergence and Wasserstein distance quantified overlap and divergence of cell population distributions (Fig. 5K).

These methods each demonstrate how relationships between individual cells can define tissue-scale cell compartments, and show how MuSpAn can quantify the distribution and interactions between tissue structures across whole tissue sections.

The previous examples characterise healthy mouse intestinal tissue, within which the interactions we have described are well known. We now consider a bespoke Xenium panel for a murine colorectal cancer model, designed to characterise spatial associations between fibroblasts and immune cell subtypes. We show how MuSpAn can be leveraged to investigate specific cell relationships and uncover new biological insight.

### Multiscale spatial profiling of fibroblast-immune associations in murine intestinal neoplasia

**Fig. 6:**
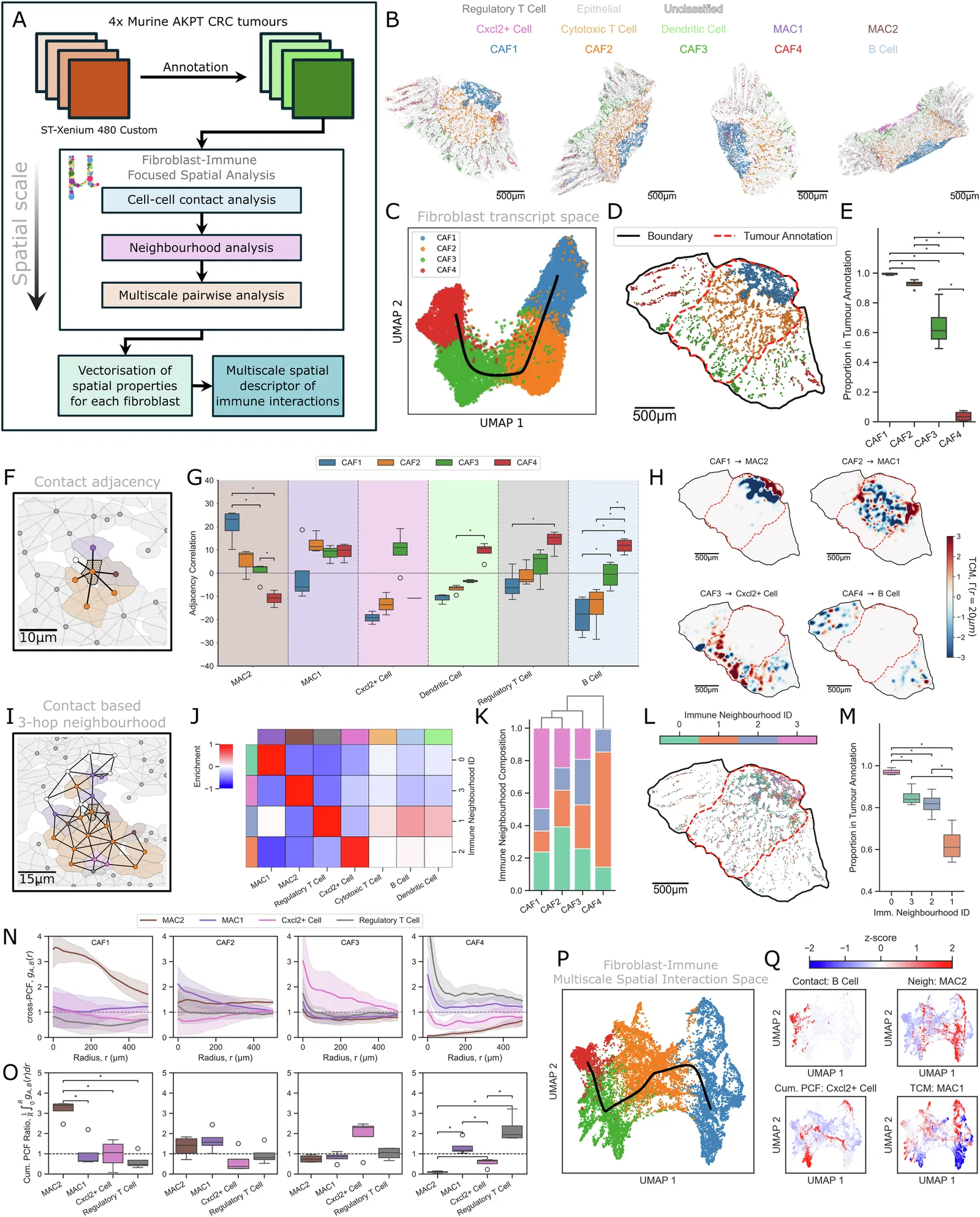
Multiscale spatial correlation of fibroblasts and immune cells in murine AKPT CRC tumours. Spatial analysis workflow overview (**A**). Cell boundaries in the murine AKPT colorectal tumour samples are coloured by cell type (**B**). Fibroblast transcriptional profiles (*n* = 16,512) in these samples are projected via UMAP (**C**), where the black line represents an inferred trajectory through transcript clusters. Fibroblasts in a representative tumour sample (**D**) show the spatial variation in CAF locations, with sample boundary (black) and pathologically annotated tumour region (red dashes). The proportion of CAF subtypes within the tumour annotation (**E**) across all samples varies substantially (*: *p* = 0.0286, Mann-Whitney, two-sided. *n* = 4 for each). We perform cell-cell contact analysis (**F**–**H**), defining an adjacency network using 1-hop neighbourhoods on segmented cell boundaries (**I**, connectivity distance <1.5 μm, coloured by cell type). An adjacency permutation test (**G**, APT) identifies fibroblast correlation with immune cell subtypes (*: *p* = 0.0286, Mann-Whitney, two-sided. *n* = 4 for each cell pair). Short range colocalisation (<20 μm) between selected fibroblasts and immune cells is quantified using topographical correlation maps (**H**, TCMs). A neighbourhood analysis (**I**–**M**) instead uses a 3-hop neighbourhood on cell boundaries (**I**, connectivity distance <1.5 μm, coloured by cell type) to produce an enrichment matrix for immune cells in 3-hop neighbourhoods centred around each fibroblast (**J**, *k*-means clustering, *k* = 4, square-root-transformed *z*-scores). The distribution of these immune neighbourhoods differs around each fibroblast subtype (**K**, coloured by neighbourhood ID), and is shown in a representative region (**L**, annotations as in D; fibroblast cell centres coloured by immune neighbourhood ID). Proportions of fibroblasts with each immune neighbourhood classification within the tumour annotation differ (*: *p* = 0.0286, Mann-Whitney, two-sided. *n* = 4). Finally, multiscale analysis of fibroblast-immune spatial relationships (**N**–**Q**) uses cross-PCFs between selected fibroblast-immune cell pairs (**N**, mean, 95% confidence intervals). The cumulative PCF ratios (**O**) summarise the first 150 μm of each cross-PCF, $\frac{1}{150}\int_{0}^{150}{g}_{A,B}(r)dr$, for each fibroblast-immune cell pair (*: *p* = 0.0286, Mann-Whitney, two-sided). All multiscale spatial immune interaction metrics (**F**–**O**) are combined across all fibroblasts (**P**, UMAP projection, coloured by fibroblast type; line represents inferred trajectory). Colouring the UMAP projection by four selected spatial interaction features shows how the immune context of the fibroblasts subtypes varies (**Q**, *z*-score: 1-hop adjacency with B cells, abundance of Macrophage Type 2 cells in 3-hop neighbourhoods, cumulative cross-PCF ratio with Cxcl2+ Cells and TCM values with Macrophage Type 1 cells). All boxplots show the median (centre line), 25th and 75th percentiles (box), and minimum and maximum values (whiskers).

Having demonstrated the capacity of MuSpAn to conduct exploratory analyses of multiscale, cross compartment cell structure in normal mouse colon tissue, we next used it for focussed analysis of pathological cancer-associated fibroblast-immune cell relationships in murine intestinal neoplasia. We undertook analysis of a bespoke 480-gene Xenium spatial transcriptomics panel on a dataset of intestinal tumours arising in *Villin-CreER*^T2^; *Apc*^fl/fl^; *Kras*^G12D/+^; *Trp53*^fl/fl^; *Tgfβr1*^fl/fl^ mice (hereafter AKPT) (*n* = 4), as they generate stromal rich, metastatic, CMS4 cancers^43^. A multiscale workflow (Fig. 6A) was constructed, analysing immune context around cancer-associated fibroblast subtypes (Fig. 6B–E) at three scales: direct cell-cell contact (⪅10 μm, Fig. 6F–H), immune neighbourhood composition (⪅60 μm, Fig. 6I–M), and pairwise spatial correlations (⪅500 μm, Fig. 6N–P).

Cell annotation identified four transcriptionally distinct cancer-associated fibroblast subtypes, spanning a trajectory from fibrotic CAF1 to myofibroblast-like CAF4^44^ (Fig. 6C). Their spatial distributions reflected this progression: CAFs 1–3 localised inside annotated tumour regions, while CAF4 cells were enriched outside the tumour periphery (Fig. 6D, E).

At the cell-cell contact scale, proximity networks (≤1.5 μm; ensuring connections only between cells in direct contact) defined 1-hop fibroblast neighbourhoods (Fig. 6F). APT revealed subtype-specific contact co-localisations: CAF1 fibroblasts with Spp1+ macrophages (MAC2), CAF3 with Cxcl2+ cells and CAF4 with B cells and dendritic cells (Fig. 6G; Supplementary Fig. 11I). Topographical Correlation Maps (TCM, *r* < 20 μm; chosen to identify correlation between cells within close proximity) highlighted regional variation in cell contact co-localisation: for example, CAF2-MAC1 contacts were concentrated at the invasive front, but absent in the tumour bulk (Fig. 6H, Supplementary Fig. 11H).

At the neighbourhood scale (≈60 μm; representing environments within approximately 3 cell diameters), 3-hop immune environments around fibroblasts were clustered by immune cell composition, yielding four neighbourhoods dominated by MAC1, MAC2, Cxcl2+ cells, or a lymphoid mixture including regulatory T cells (Tregs) respectively (Fig. 6I, J; Supplementary Note 6). CAF1 cells were surrounded by MAC2-dense neighbourhoods (51%), while CAF4 cells were strongly associated with Treg cell environments (67%) (Fig. 6K). Spatial differences in the localisation of the different immune neighbourhoods were evident: macrophage- and Cxcl2+-rich neighbourhoods were localised within the tumour mass, while adaptive cell-enriched neighbourhoods mapped to the periphery (Fig. 6L, M).

At the tissue scale (≈500 μm; representing a substantial section of each ROI), cross-PCFs confirmed these associations: CAF1 cells positively correlated with MAC2 cells, and were excluded from Treg cells, whereas CAF4 cells co-localised with Tregs and were excluded from MAC2 cells (Fig. 6N, O).

To integrate findings across scales, we constructed an 87-dimensional summary vector for each fibroblast, consisting of vectorised metrics describing adjacency, neighbourhood composition and correlation structure (see ‘Methods’). This feature space revealed clear segregation of fibroblast subtypes, with spatial association profiles closely aligned with transcriptional states (Fig. 6P). Trajectory inference further supported a continuum of fibroblast-immune associations that mirrors transcriptional progression. Notably, this alignment is not driven by tumour compartmentalisation: the trajectory structure and its correspondence with transcriptional state are preserved when the analysis is restricted to tumour-resident fibroblasts only (see Supplementary Information, Supplementary Note 6).

This analysis demonstrates MuSpAn’s capacity to reveal structured stromal-immune organisation across spatial scales. Transcriptionally and spatially distinct cancer associated fibroblast populations generate discrete immune niches, with CAF1 located in central tumour regions which are macrophage-rich and CAF4 cells concentrated in adaptive immune cell-enriched regions outside the tumour periphery. These patterns recapitulate hallmark features of CMS4 tumours, including immunosuppressive macrophage enrichment, fibroblast heterogeneity and compartmentalised immune exclusion. By integrating spatial data from direct contact to tissue-wide co-localisation, MuSpAn reveals coordinated stromal-immune organisation across scales, providing a spatial framework consistent with reported links between stromal architecture and immune evasion or therapy resistance^45,46^.

### Enabling reproducible and customisable spatial analysis

MuSpAn provides a flexible toolbox which can be applied in many ways and contexts. Central to this is MuSpAn’s ability to easily reproduce existing data analysis pipelines, proposed for specific biological contexts and data types. Further, MuSpAn makes extending or adapting such pipelines trivial. This is demonstrated in the case studies in the preceding sections, which have adapted existing pipelines for exploratory data analysis (in the healthy tissue example) and for biologically-driven pipeline development (in the AKPT tissue example). Further case studies are presented in the Supplementary Information (Supplementary Note 1), where we reproduce previous data analysis workflows on datasets from COVID-19 lung tissue, head and neck cancer and colorectal cancer tissue microarrays. These examples further demonstrate the platform agnostic nature of MuSpAn, with datasets from co-registered IHC slides, CODEX and imaging mass cytometry. The flexibility of MuSpAn’s ‘library’ approach differs from commonly used ‘pipeline’ approaches to spatial data analysis, and, as such, care must be taken to ensure that pipelines constructed using MuSpAn are relevant to the research question of interest. An in-depth discussion of key factors to consider when developing an effective biologically-driven pipeline is present in the Supplementary Information (Supplementary Note 2).

## Discussion

Spatial biology enables precise mapping of cell populations, their interactions and structural arrangements within complex tissues. By quantifying intercellular organisation, spatial analysis has become a powerful driver of biological discovery. Yet, the diversity of imaging modalities, scales of analysis and analytical frameworks makes it difficult to extract consistent biological insights. Existing tools are often specialised for a single imaging platform or a small set of questions, limiting researchers’ flexibility to compare or integrate methods across scales (see the Supplementary Information, Supplementary Note 4, for a detailed comparison between MuSpAn and selected spatial biology packages).

MuSpAn addresses this gap by offering a versatile, multiscale platform for spatial analysis in a modality-agnostic manner, compatible with diverse imaging and omics technologies. It provides streamlined access to over 100 methods drawn from geometry, topology, network theory, spatial statistics, optimal transport and probability. By allowing analyses at one scale to inform those at another, MuSpAn helps quantify biological complexity that may otherwise be overlooked. Here, we have demonstrated the application of MuSpAn for analysis of spatial datasets from the subcellular level through to tissue structural organisation in mouse normal and neoplastic intestinal datasets. We have highlighted the convergent use of diverse mathematical tools to identify and quantify cell morphology, distribution and cross-compartmental spatial associations and demonstrated the power of this for biological discovery.

MuSpAn emphasises flexibility, supporting both detailed exploratory analyses in discovery biology and scalable, high-throughput workflows. Users can easily benchmark and cross-validate complementary methods within the same dataset, overcoming the limitations of relying on any single analytic approach and providing confidence that identified cell associations are genuine. Comprehensive online resources, including documentation, detailed tutorials and scientific case studies, support spatial data exploration and foster community engagement (https://www.muspan.co.uk). MuSpAn’s user-focussed design permits most statistics to be executed and visualised with a single line of code, and moderately sized datasets can be analysed in seconds on a standard laptop without requiring high performance computing resources (see Supplementary Information, Supplementary Note 7). MuSpAn’s *query* system is intuitive for routine use while enabling highly customisable spatial data filtering for advanced investigations. These features combine to maximise flexibility and foster innovation, empowering researchers to rapidly develop and refine bespoke spatial analysis pipelines.

Beyond exploratory analysis, MuSpAn complements the growing role of AI in spatial biology. While AI excels at pattern recognition, it often lacks interpretability^47^. MuSpAn delivers directly interpretable metrics that provide mechanistic insight (e.g. describing cell-cell contact, exclusion zones, morphology). Metrics can be vectorised for integration into AI workflows, bridging interpretable quantification and predictive modelling (an approach successfully applied in TDA^48,49^). Agentic AI currently relies on lower-level implementations of code to conduct data analyses, indicating that dedicated analytical packages will remain important even as AI-authored pipelines evolve. Although these systems may increasingly automate spatial analyses, reliable biological interpretation will still depend on domain-specific tools that provide robust and transparent quantification.

Looking ahead, MuSpAn is positioned to address challenges in integrating spatial data with other modalities including single-cell RNA sequencing, as well as with patient metadata to link spatial patterns to clinical outcomes. Its architecture supports future extensions to 3D and longitudinal datasets, enabling spatio-temporal analyses that capture dynamic tissue reorganisation. By combining accessibility, mathematical depth and multiscale capability, MuSpAn bridges the gap between advanced imaging technologies and interpretable analytics, and complements the wider community of tools for exploratory spatial biology^14,20,37,50^. Alongside these tools, MuSpAn empowers researchers to move beyond qualitative mapping toward mechanistic, quantitative and multiscale biological insight.

## Methods

This research complies with all relevant ethical regulations; all procedures were carried out in accordance with Home Office UK regulations (including approval by the local animal welfare ethical review committee) and the Animals (Scientific Procedures) Act 1986.

### MuSpAn

#### Overview

MuSpAn provides a general framework for conducting spatial analyses. In this section, we summarise its structure, and explain how this facilitates flexible and targeted spatial analyses which can be applied to any imaging modality or data source. We emphasise that MuSpAn is designed for spatial analysis, and not for preprocessing of raw imaging data. Cell or object identification, region annotation and labelling should be conducted prior to spatial analysis, using available pipelines or image analysis software (e.g. Xenium explorer, QuPath, Halo, Deepcell). MuSpAn imports array-like data directly within Python - by reading CSV files with libraries like Pandas, or using Numpy arrays - and provides additional ‘quality of life’ tools to import annotations or labels from software such as QuPath or Xenium Explorer.

In the following sections, we outline MuSpAn’s design, key features and how it enables highly flexible spatial analysis. While many features are not user-facing, and therefore do not require user understanding, understanding the infrastructure supporting MuSpAn can aid more advanced use.

##### Summary

All MuSpAn analyses begin with the *domain*, a class which includes data, labels and metadata. The domain can be viewed as a container to which data is added; this standardises the internal representation of the data so they can be interactively queried and/or analysed by the user. This ensures that analysis methods are not called on incompatible data (e.g. requesting the area of point-like data), that data can easily be filtered for subsequent analysis, and permits straightforward expansion of the spatial analysis toolbox.

Spatial data are stored within the domain as *objects*, which represent distinct biological entities as points or shapes - the interpretation of objects can vary. For example, both transcripts and cell centres are represented as point objects, while cell boundaries, manually specified annotations and region of interest boundaries are shape objects. Each object can have metadata associated as *labels*, which can be used to isolate populations of interest for statistical analysis. By ensuring all data within a domain are held in a standardised format, the data can be easily interrogated using individual quantitative analysis methods, and results visualised within MuSpAn, or returned as raw data for subsequent visualisation or onward use outside of MuSpAn.

##### Domain

A MuSpAn *domain* is a class which standardises the structure of spatial data and metadata, and can be thought of as a container within which objects for analysis are located. Biologically, it represents a single area containing all relevant data for analyses within the same spatial frame of reference, such as a region of interest within an image where all cell (or transcript) locations are represented within the same coordinate system. The domain is enclosed by a continuous closed *boundary*, which defines a simply connected space that fully contains all objects.

When adding new objects to the domain outside of the existing boundary, the boundary of the domain updates by default to be the axis aligned bounding box surrounding all objects; however, the boundary can be easily changed to any non-self intersecting polygon which encloses all objects, such as the convex hull, an alpha shape that surrounds the objects, or an annotation made in external software (for instance, the boundaries of the domains shown in Figs. 2–5 are annotations drawn within Xenium explorer).

The domain also contains helpful metadata, such as colourmaps for visualisation of objects under different criteria (e.g. for colouring objects according to associated labels), or caches to ensure computationally expensive calculations such as distances between objects or network representations of the data can be retained where needed.

##### Objects

MuSpAn *objects* represent biological entities as either *points* or *shapes*, each assigned a unique Object ID. Points are defined by (*x*, *y*) coordinates, while shapes are non-self-intersecting polygons defined by ordered coordinate lists. Shapes can include complex features like internal voids, allowing them to represent diverse regions such as cell boundaries, tumour zones, or areas for exclusion in spatial analyses.

Each object’s Object ID links it to metadata, including labels, spatial properties, networks, distances and other features. Objects can be added or removed from a domain and may store pre-calculated attributes, such as area, perimeter, bounding circles for efficient distance calculations, or relationships to other objects (e.g. parent-child links created through transformations like shape decomposition). The spatial distance between two objects is defined as the minimum Euclidean distance, accommodating both point-like and shape-like objects. For shapes, distances can be calculated using the centroid or via the nearest part of the relevant line segment in the shape boundary.

Objects can belong to one or more collections, which are named subsets of objects (e.g. ‘cell boundaries’, ‘transcripts’, or ‘damaged tissue’). A collection denotes a subset of objects, and is one way of referring to multiple objects at once. Collections have no restrictions on names or overlapping membership.

##### Labels

The main mechanism by which MuSpAn associates metadata with an object is through the use of *labels*. Each object may be assigned any number of different labels, which may be categorical (discrete) or numeric (continuous). For example, in Fig. 2D–G, cells are shape objects that have been assigned a categorical label ‘Celltype 1’ or ‘Celltype 2’, as well as numerical labels ‘Area’, ‘Principal angle’ and ‘Circularity’. Labels can be assigned to some subset of objects (for instance, all objects in one collection), either based on metadata obtained from outside of MuSpAn (e.g. labelling cells from multiplex immunohistochemistry according to their mean pixel intensity of a marker of interest) or from within MuSpAn (e.g. labelling cells according to the result of some spatial analysis of interest). In Fig. 2D–G, the celltype label is based on the preprocessing steps conducted in Xenium explorer, while the other labels are derived from geometric summary statistics calculated for each cell within MuSpAn and subsequently added to the shapes. Labels can be used for analysis or visualisation, or as part of the calculation of other spatial metrics.

##### Moving between spatial scales

As indicated in Fig. 1D, a key way of moving between spatial scales in MuSpAn is by defining new objects based on existing ones. Starting from the lowest spatial scale added to the domain (in our examples, point objects representing transcript locations), we can move up spatial scales by combining transcript locations into a single cell object (using the segmentation mask if available, or by finding, for instance, the convex hull or *α*-shape of the points). Converting a shape into a single point (e.g. turning a cell boundary into a cell centroid) also moves up spatial scales by ‘simplifying’ a collection of more complex spatial information into a single object. These processes effectively lower the resolution of the object or objects in question (moving from a set of points to a single shape, or from a detailed shape boundary to a single centroid), which can then be considered in the context of other objects at the same spatial scale without becoming computationally infeasible.

Data can be transferred across spatial scales through the use of labels, which allow a ‘coarse grained’ object to contain information about the ‘fine grained’ objects from which is has been derived—for instance, a cell centroid could be labelled according to the circularity or area of the parent cell boundary object. This allows features computed at one scale to be inherited by the new ‘child’ objects derived from the ‘parent’. Parent-child relationships are stored within the domain as new objects are created, which allows easy construction of ancestry trees for multiscale information retrieval.

##### Queries

MuSpAn provides powerful query tools to filter and analyse objects based on specific properties, enabling both straightforward and advanced data interrogation. While most users benefit from this infrastructure automatically, advanced users can directly create and use queries to extract complex data subsets using the query module. A query in MuSpAn specifies criteria for selecting objects. It comprises three components:

1. a property of interest (e.g. the label ‘Celltype’);
2. a relation (e.g. ‘is equal to’);
3. a comparison value (e.g. ‘T cell’).

These components would generate a query that returns any objects with a ‘Celltype’ label equal to ‘T cell’. This query is evaluated at runtime to produce a boolean array (i.e. True for matching objects, False otherwise). Once created, queries can be reused across various functions, including spatial analysis and visualisation tools.

Users can construct queries explicitly or rely on MuSpAn’s intuitive syntax for common cases. For example, passing the tuple (‘Celltype’, ‘T cell’) to a function will automatically lead to its interpretation as a query relating to the ‘Celltype’ label. Advanced queries can combine multiple criteria using query containers, which support logical operations (e.g. AND, OR). These containers allow nesting of conditions, enabling highly detailed filtering. For example, a query container might select all T cells within 20 μm of a tumour boundary, or even more complex combinations of spatial relationships, label values and even summary statistics.

Queries persist in the Python workspace, allowing consistent use across analysis and visualisation methods. While beginner users can rely on MuSpAn’s default behaviour and ‘behind the scenes’ use of the query infrastructure to conduct basic analyses, leveraging the query system provides a powerful way to explore and analyse data efficiently. Tutorials and examples of advanced queries are provided on the MuSpAn website.

##### Visualisation

While not primarily a visualisation package, MuSpAn leverages Python’s matplotlib package^51^ to provide easy access to data visualisation tools through MuSpAn’s visualise module. In particular, this permits straightforward visualisation of the domain and objects within it, coloured according to labels, collections, or other properties of interest. Domain visualisation accepts query arguments to specify the objects to plot or the metadata used to colour the output, permitting easy publication-ready plotting of complex subsets of objects; almost all the panels in this manuscript are generated using the visualisation tools available within MuSpAn, and detailed tutorials showing how this is achieved are available on the MuSpAn website and alongside this work.

MuSpAn can also be used to natively plot the outputs of selected spatial analysis methods. Every MuSpAn spatial analysis method returns the output as raw data that can be passed by the user to other analysis or visualisation tools; however, this data can also be passed to dedicated functions within the visualise module to straightforwardly generate plots. These functions can also be called automatically using the visualise_output keyword argument associated with most spatial analysis methods. While the visualise module provides customisation via matplotlib, it is generally recommended that users use raw outputs for creating custom visualisation.

#### Quantitative analyses

Central to MuSpAn are the quantitative analyses it provides. All of the methods available within MuSpAn v1.2.5 have been previously described in the academic literature, and so are not described in detail here; a complete list of implemented methods, with references to their use in spatial biology where possible, can be found in the Supplementary Information. Where possible, MuSpAn leverages implementations of individual methods from established discipline-specific Python packages^52–54^.

The spatial analysis methods provided within MuSpAn are divided between six submodules, named according to the underlying mathematical framework within which the analysis methods are contained. These modules are:

- **Distribution**—containing methods through which spatial data is considered as a continuous distribution, such as a density function.
- **Geometry**—containing methods that describe the morphology or other characteristics of shapes.
- **Networks**—containing methods through which data is represented as spatially embedded networks.
- **Region Based**—containing methods which represent data within spatially-averaged subregions of the region of interest.
- **Spatial Statistics**—containing methods from the field of spatial statistics, which compare observations of point data against statistical null models.
- **Topology**—containing methods from the field of topological data analysis, which describes data in terms of topological invariants.

We reiterate here that each method within these modules is designed to investigate different types of spatial relationships. As such, techniques from any module can be applied at any length scale. To generate pipelines that are robust to potential error introduced by viewing data through the lens of one particular methodology, we suggest that methods from across multiple submodules should be employed where possible (see the Supplementary Information, Supplementary Note 2).

##### Specifying populations

Each of the functions available within MuSpAn for spatial analysis accepts different classes of objects; for example, spatial statistics such as the cross-PCF require two point populations to be defined, while a geometric statistic such as convexity is only defined for shape-like (polygonal) objects. Each statistic allows the population (or populations) to be specified when calculated, for instance by using the MuSpAn query module described above. This permits subsets of the data to be isolated and analysed very easily, and for the same population to be passed to multiple functions in series, making pipeline construction simple.

##### Specifying analysis boundaries

Many statistics are dependent not only on the population of objects they consider, but also on the boundaries of the analysis. As a simple (non spatial) example, the density of a fixed number of cells varies depending on the size of the region in which they are measured. MuSpAn permits analysis boundaries to be specified via queries in the same way as populations are. Users can specify a set of shapes that define the boundaries of a calculation region, or of shapes defining regions that should be excluded from analysis. MuSpAn converts these requests on-the-fly into simply connected spaces for analysis, and where appropriate adjusts statistical analyses to account for boundary corrections. Where no boundary is specified, analyses will use the domain boundary. This functionality enables users to assess the impact of compartmentalisation on spatial structure, for instance by comparing the value of within-compartment statistics against those calculated ignoring compartment boundaries. See Supplementary Note 1 in the SI for examples of spatial analysis within shape boundaries.

### Spatial analysis methods overview

MuSpAn v1.2.5 (used for the analyses in this manuscript) includes a diverse collection of 40+ quantitative analysis methods which have been described in the scientific literature. Table 1 summarises the spatial methods used in the exemplar analyses shown in Figs. 2–6, and provides references containing detailed methodological descriptions (for additional information see the SI, Supplementary Note 5). A complete list of all spatial analysis methods implemented in MuSpAn v1.2.5 is provided in the SI (Supplementary Note 3). In addition, detailed information on all 150+ MuSpAn functions can be found at https://docs.muspan.co.uk.

**Table 1:** Overview of spatial analysis methods featured in Figs. 2–6

Figure	Method (MuSpAn module)	Summary	Ref
2	Circularity (geometry)	A measure of how similar the shape of a polygon is to a circle, with values ≈1 indicating shapes that are closer to perfectly round.	-
2	Principal angle (geometry)	The angle of the longest line through the centre of a polygon that best represents its overall orientation, often used to describe a shape’s direction or alignment.	^55^
2	Label composition (summary_statistics)	The count of a specific label category, normalised relative to the total number of labels in the region.	-
3	Average nearest neighbour index (ANNI) (spatial_statistics)	A statistical measure used to determine whether a spatial distribution of points is clustered, random, or dispersed by comparing observed and expected nearest neighbour distances.	^40^
3	Proximity network (networks)	A network that connects nodes based on their spatial distance.	^56^
3	Quadrat correlation matrix (QCM) (spatial_statistics)	A measure of correlation between the counts of different cell types within a set of regions.	^57^
4	Cross pair correlation function (cross-PCF) (spatial_statistics)	A measure of spatial aggregation between pairs of objects across length scales.	^58^
4	Vietoris-Rips filtration (topology)	A method in topological data analysis that constructs a nested sequence of simplicial complexes by connecting points within increasing distances, capturing the shape and features of a dataset across scales.	^52^
5	Delaunay network (networks)	A network where nodes in a plane are connected to form triangles such that no point lies inside the circumcircle of any triangle.	^56^
5	*k*-hop neighbourhood (networks)	The set of all nodes that can be reached from a given node within *k* steps or connections.	-
5	Neighbourhood clustering (networks)	Group similar neighbourhoods together based on the enrichment of labels within the neighbourhoods.	^56^
5	Adjacency permutation test (networks)	A statistical method that assesses the significance of node label adjacency by randomly permuting node labels and comparing the observed property to the distribution from the permutations.	^59^
5	*α*-shape (helpers)	A method to define the shape of a set of points in space by connecting points that are close to each other, using a parameter *α* to determine the length scale captured.	-
5	Hexgrid (region_based)	Generate a mesh of regular hexagons covering the requested space.	-
5	Local Getis-Ord^*^ (spatial_statistics)	A statistic that identifies spatial clusters by measuring the degree of local spatial association for a variable, highlighting areas of high or low values relative to their surroundings.	^60^
5	Kernel density estimation (KDE) (distribution)	A non-parametric statistical method for estimating the probability density function of a random variable by smoothing data points using a kernel function.	^53^
5	Wasserstein distance (distribution)	A metric for comparing probability distributions by measuring the optimal transport cost required to move one distribution to another.	^33^
5	KL-divergence (distribution)	A measure of the difference between two probability distributions by quantifying the amount of information lost when approximating one distribution with another.	^61^
6	Topographical correlation map (TCM) (spatial_statistics)	A Local Indicator of Spatial Association (LISA) which quantifies the pairwise correlation between two populations of points and is visualised locally as a heatmap.	^62^
6	Ripley’s K-function (spatial_statistics)	A measure of pairwise spatial correlation between two populations of points, based on comparing the observed numbers of points within a minimum pairwise distance of one another.	^58^
6	Empty space function (spatial_statistics)	A function which characterises empty spaces by repeatedly sampling random locations and measuring the distance to the nearest point.	^58^

We indicate each method’s module within the MuSpAn package and provide a concise description. For full details on the definitions of these methods, please see the references provided. References for standard methods are omitted (denoted by ‘-’).

### Datasets

#### Healthy murine colon—Xenium

Spatial analysis was conducted on murine colon tissue stained with the Xenium Mouse Tissue Atlas Panel (10x Genomics, USA), using dataset ‘Fresh Frozen Mouse Colon with Xenium Multimodal Cell Segmentation’ sourced from the 10x Genomics website and available under a CC BY license (available from https://www.10xgenomics.com/datasets/fresh-frozen-mouse-colon-with-xenium-multimodal-cell-segmentation-1-standard). Fresh-frozen tissue arrays were prepared from 8-week-old male C57 mice (Charles River Laboratories, USA). Tissue samples were processed according to the Xenium protocols for fresh-frozen tissues, following the Xenium In Situ for Fresh Frozen Tissues—Tissue Preparation Guide (CG000579) and Fixation & Permeabilisation Guide (CG000581). Staining procedures involved probe hybridisation, washing, ligation, amplification and cell segmentation staining, carried out in alignment with the Xenium In Situ Gene Expression with Cell Segmentation Staining User Guide (CG000749). Post-instrument processing, including hematoxylin and eosin (H&E) staining, followed the Xenium In Situ Gene Expression - Post-Xenium Analyser H&E Staining Protocol (CG000613). The Xenium Mouse Tissue Atlas Panel, pre-designed by 10x Genomics, includes 379 genes selected to represent major cell types and optimised for spatial gene expression profiling in mouse tissues.

Transcript clustering was provided with the Xenium experiment, which assigned 25 distinct cluster IDs to cells using a graph-based approach (Leiden clustering). Cluster IDs 1–19 were considered in our demonstrative analysis due to low observations of cells with Cluster IDs 20–25. The 19 retained clusters were subsequently annotated on the basis of the differential expression matrix provided by 10x Genomics (Table 2).

**Table 2:** Xenium cluster labels

Cluster ID	Celltype (long)	Celltype (short)
Cluster 1	Smooth Muscle Cell	SMC
Cluster 2	Epithelial Progenitor	PROG
Cluster 3	Epithelial Type 1	EPI1
Cluster 4	Macrophage Type 1	MAC1
Cluster 5	Colonocyte	COL
Cluster 6	Epithelial Type 2	EPI2
Cluster 7	Epithelial Type 3	EPI3
Cluster 8	Epithelial Type 4	EPI4
Cluster 9	Macrophage Type 2	MAC2
Cluster 10	Endothelial Type 1	END1
Cluster 11	Stromal Cell Type 1	STR1
Cluster 12	Stromal Cell Type 2	STR2
Cluster 13	Epithelial Type 5	EPI5
Cluster 14	Stromal Cell Type 3	STR3
Cluster 15	Epithelial Type 6	EPI6
Cluster 16	Stromal Cell Type 4	STR4
Cluster 17	Endothelial Type 2	END2
Cluster 18	Neural Cell	NEU
Cluster 19	Epithelial Type 7	EPI7

Transcript-defined cell clusters with corresponding full and abbreviated cell type annotations from the healthy murine colon Xenium dataset.

#### AKPT murine colorectal cancer—Xenium

We utilised the *Villin-CreER*^T2^; *Apc*^fl/fl^; *Kras*^G12D/+^; *Trp53*^fl/fl^; *Tgfβr1*^fl/fl^ (*n* = 2) or *Villin-CreER*^T2^; *Apc*^fl/+^; *Kras*^G12D/+^; *Trp53*^fl/fl^; *Tgfβr1*^fl/fl^ (*n* = 2) (AKPT) murine model of colorectal cancer. Driver alleles were initiated as follows: A is *Apc*^fl/fl^, K is *Kras*^G12D^, P is *p53*^fl/fl^, T is *Tgfβr1*^fl/fl^. This combination recapitulates the genetic and microenvironmental features of aggressive, stromal rich, colorectal cancer. All animals were on C57BI/6J background. Both females and males have been used in this study, we did not report sex as a variable in our mouse experiments. All procedures were carried out in accordance with Home Office UK regulations (including approval by the local animal welfare ethical review committee, the University of Oxford Animal welfare ethical review board (AWERB)) and the Animals (Scientific Procedures) Act 1986. All mice were housed at the Functional Genetics Facility (Centre for Human Genetics, University of Oxford).

Gut preparations were washed in PBS, fixed overnight in 10% neutral buffered formalin and then transferred to 70% ethanol before processing for embedding. Formalin-fixed gut sections were rolled into Swiss Rolls, pinned and placed in a histology cassette. Specimens were processed using a Histomaster machine (Bavimed). Processed samples were embedded in paraffin wax using a paraffin embedding station (EG1150H, Leica). 5 μm formalin-fixed, paraffin-embedded tissue sections were used on Xenium slides. Slides were baked at 42 ^∘^C for 3 h. The sections stained following the manufacturers protocol with a custom 480 panel of genes with Xenium v1 chemistry.

Seurat (v5.2.0) was used in R (v4.3.3) to annotate four AKPT murine CRC Xenium samples using transcript profiles, which were merged, normalised and clustered, with epithelial-rich clusters identified by Epcam and Cdh1 expression and removed. The remaining non-epithelial cells were re-clustered at higher resolution to identify fibroblast (*Pdgfra*+) and immune (*Ptprc*+) populations, with classification based on scaled aggregate gene expression across 22 clusters. Immune clusters were further subtyped using a marker-based heatmap (ComplexHeatmap v2.18.0), resulting in a low-resolution annotation of 12 immune cell populations. For further information on sample annotation, see Supplementary Note 6.

### Multiscale spatial profiling of fibroblast-immune interactions in colorectal tumours

To investigate fibroblast-immune interactions across spatial scales, we applied a series of spatial statistical and network-based analyses using the MuSpAn. For each fibroblast type of interest, we quantified spatial context of immune interactions at three spatial scales: (i) direct cell-cell contact, (ii) neighbourhood composition and (iii) multiscale (0–500 μm) spatial association.

At the cell-cell contact level, we built proximity networks from cell boundaries (contact distance ≤1.5 μm) to define immediate (1-hop) adjacency immune cells and computed an *Adjacency Permutation Test* (APT) to identify correlations between neighbouring fibroblasts and immune cells. We also computed *Topographical Correlation Maps* (TCMs) to quantify the spatial significance of resultant co-localised pairs from the APT. At an intermediate scale, extended (3-hop) neighbourhoods centred on the selected fibroblasts in the proximity network were computed and clustered by immune composition using *k*-*means* with *k* = 4, selected to yield distinct and interpretable immune microenvironments around each fibroblast. For larger length scales, we used the *cross-pair correlation function* (cross-PCF) to quantify spatial associations between fibroblast and immune populations from 0 to 500 μm.

The above measurements were aggregated into an 87-dimensional feature vector for each fibroblast across all samples. For each fibroblast, the features include:

- Number of direct cell-cell contacts with each immune subtype (7);
- Number of cells within a 3-hop contact neighbourhood with each immune subtype (7);
- TCM value at the fibroblast’s location with respect to each immune subtype at *r* = 30 μm (7)
- Contribution of fibroblast to the respective cross-PCF from all fibroblasts to each immune and fibroblast subtype at 0, 100, 200, 300, 400 μm (55);
- Cumulative cross-PCF ratio up to *R* = 150 μm for each immune and fibroblast subtype (11).

Feature vectors were *z*-score normalised and summarised medians were computed for each fibroblast type. Pairwise distances between fibroblast type median summary vectors were used to construct a weighted minimum spanning tree, which was projected into UMAP space to visualise an inferred trajectory of fibroblast-immune states. For an extended description of methods, see Supplementary Note 6.

### Reporting summary

Further information on research design is available in the Nature Portfolio Reporting Summary linked to this article.

## Supplementary information

Supplementary Information

Reporting Summary

Transparent Peer Review file

## Source data

Source Data

## Data Availability

The ‘healthy murine colon’ dataset used in Figs. 1–5 is available from 10x Genomics (https://www.10xgenomics.com/datasets/fresh-frozen-mouse-colon-with-xenium-multimodal-cell-segmentation-1-standard). MuSpAn domains containing the cell location data used in Figs. 2–6 are provided at https://doi.org/10.5281/zenodo.17176282. The synthetic dataset used in Supplementary Fig. 9 is provided within the MuSpAn package, via the command muspan.datasets.load_example_domain(‘Synthetic-Points-Architecture’). Source data are provided with this paper.

## Code availability

MuSpAn is freely available for non-commercial use via the MuSpAn website (https://www.muspan.co.uk/get-the-code) under an Academic Use Licence. Commercial users should contact Oxford University Innovation (https://innovation.ox.ac.uk/). Comprehensive user guides including installation, documentation, and detailed tutorials are available via https://docs.muspan.co.uk. Scripts that reproduce the analysis conducted in this paper can be found at https://github.com/MuSpAn-Multiscale-Spatial-Analysis/Supporting_material_muspan_paper, or https://doi.org/10.5281/zenodo.20933039. A public Github repository for issues tracking, feature suggestion, and community discussion, is available at https://github.com/MuSpAn-Multiscale-Spatial-Analysis/MuSpAn-Public.
