# Supplementary material for: MuSpAn: a toolbox for multiscale spatial analysis
Source: Nat Commun. 2026 Aug 13;17:9737. doi: 10.1038/s41467-026-75649-7 (PMC13562714; doi:10.1038/s41467-026-75649-7)
Supplement: Supplementary file 1 [file 41467_2026_75649_MOESM1_ESM.pdf]

Supplementary Note 1 Application of MuSpAn to other imaging modalities

We demonstrate how MuSpAn can be used to reproduce and extend existing data analysis pipelines across diverse imaging modalities. To illustrate its versatility, we present three case studies:

- Head and neck cancer tissue, imaged via stacked, registered IHC slides [S1];
- COVID-19 infected lung tissue, imaged with Imaging Mass Cytometry [S2];
- Colorectal cancer tissue microarrays, imaged using the CODEX platform [S3].

In each case, MuSpAn replicates published analyses and augments them with additional methods (Supplementary Fig. 1). Note that each pipeline could be indefinitely extended straightforwardly using alternative MuSpAn components; these examples are chosen to complement the methods used in previous studies. Code is available in the Supplementary Information GitHub repository: https://github.com/joshwillmoore1/Supporting_material_muspan_paper. Further examples, including applications to Visium data, are provided on the MuSpAn website: https://docs.muspan.co.uk/latest/tutorials.html.

Supplementary Note 1.1 Macrophage localisation in head and neck cancer (IHC)

We use MuSpAn to analyse stacked IHC images from a resected human head and neck cancer sample [S1, S4] (Supplementary Fig. 2). The dataset comprises four consecutive tissue slices stained with PanCK (epithelial cells), CAIX (hypoxia), pimonidazole (severe hypoxia), and CD68 (macrophages) (Supplementary Fig. 2A). These slides were registered to form a single composite image (Supplementary Fig. 2B). The ROI used in this analysis is available as an exemplar domain within MuSpAn and can be loaded via `muspan.datasets.load_dataset('Macrophage-Hypoxia-ROI')` (Supplementary Fig. 2C). Macrophage centres are represented as point objects, while PanCK, pimonidazole, and CAIX boundaries define shape objects.

In the original study [S1], the pair correlation function (PCF) and J-function were used to assess macrophage infiltration into unannotated tumour nests. Using MuSpAn, we replicate these analyses and extend them by using MuSpAn's `include_boundaries` and `exclude_boundaries` keywords to compare stroma and tumour regions. Additionally, we compute kernel density estimates (KDEs) to visualise macrophage density, both across the entire ROI and within separate tissue compartments.

We first demonstrate that MuSpAn reproduces the spatial statistics used in the original analysis, specifically the macrophage-macrophage cross-PCF and J-function (Supplementary Fig. 2D-E). Calculating the cross-PCF across the entire ROI (blue dashed line) reveals short-range exclusion - reflecting the physical spacing between adjacent macrophage cell centres - and clustering at distances beyond around 20 pixels (17.6µm). At this length scale, macrophages are approximately 2.25 times more likely to be found near another macrophage than if they were randomly distributed.

In the original pipeline, the ROI was treated as a uniform square without distinguishing tumour and stroma regions. Using MuSpAn's `include_boundaries` and `exclude_boundaries` keywords, we extend the analysis to account for these compartments. Both the cross-PCF and J-function show stronger clustering within tumour regions (green line) compared to stroma (red line). Specifically, two stromal macrophages are around 1.75 times more likely than random to be 20µm apart, while tumour macrophages are around 2.75 times more likely - an insight not accessible via the

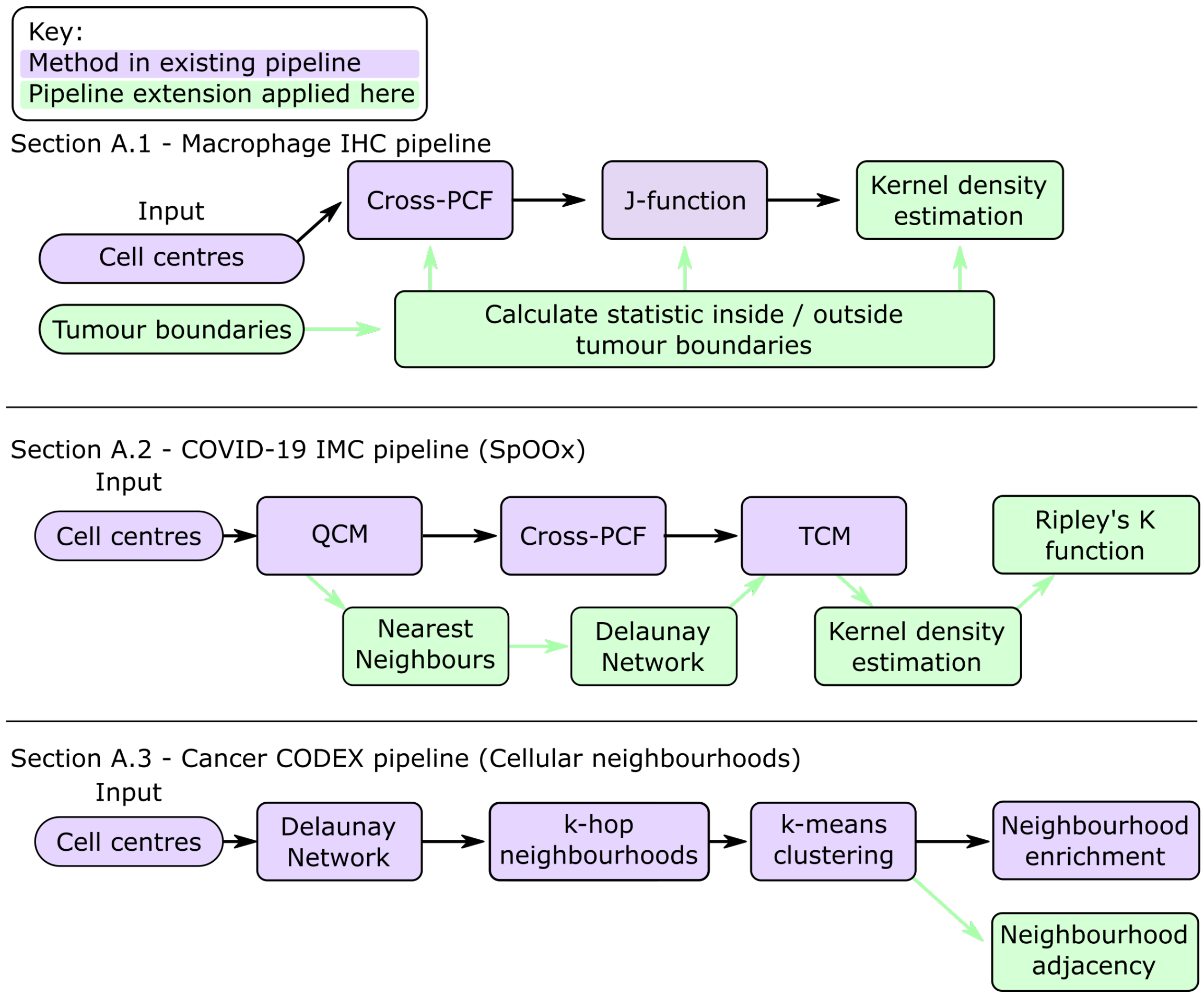

Supplementary Figure 1: **Case studies using MuSpAn to reproduce and extend existing pipelines.**
Spatial quantification components of three previously published pipelines used for spatial data analysis (A.1: [S1]; A.2: [S2]; A.3: [S3]). Key: Purple boxes - existing analyses. Green boxes - methods used to extend each pipeline here.

original pipeline. MuSpAn automatically adjusts statistical calculations to account for edge effects introduced by the new boundaries.

We further extend the analysis by computing KDEs to visualise macrophage density across different compartments (Supplementary Fig. 2F-H). The global KDE (Supplementary Fig. 2F) highlights a dense macrophage cluster in the upper right of the domain. When restricted to the stroma (excluding PanCK shapes), this cluster dominates the density map (Supplementary Fig. 2G). In contrast, the KDE within tumour regions (PanCK shapes) reveals that the tumour island with the highest macrophage density is in fact in the lower left (Supplementary Fig. 2H), which is obscured in the global view.

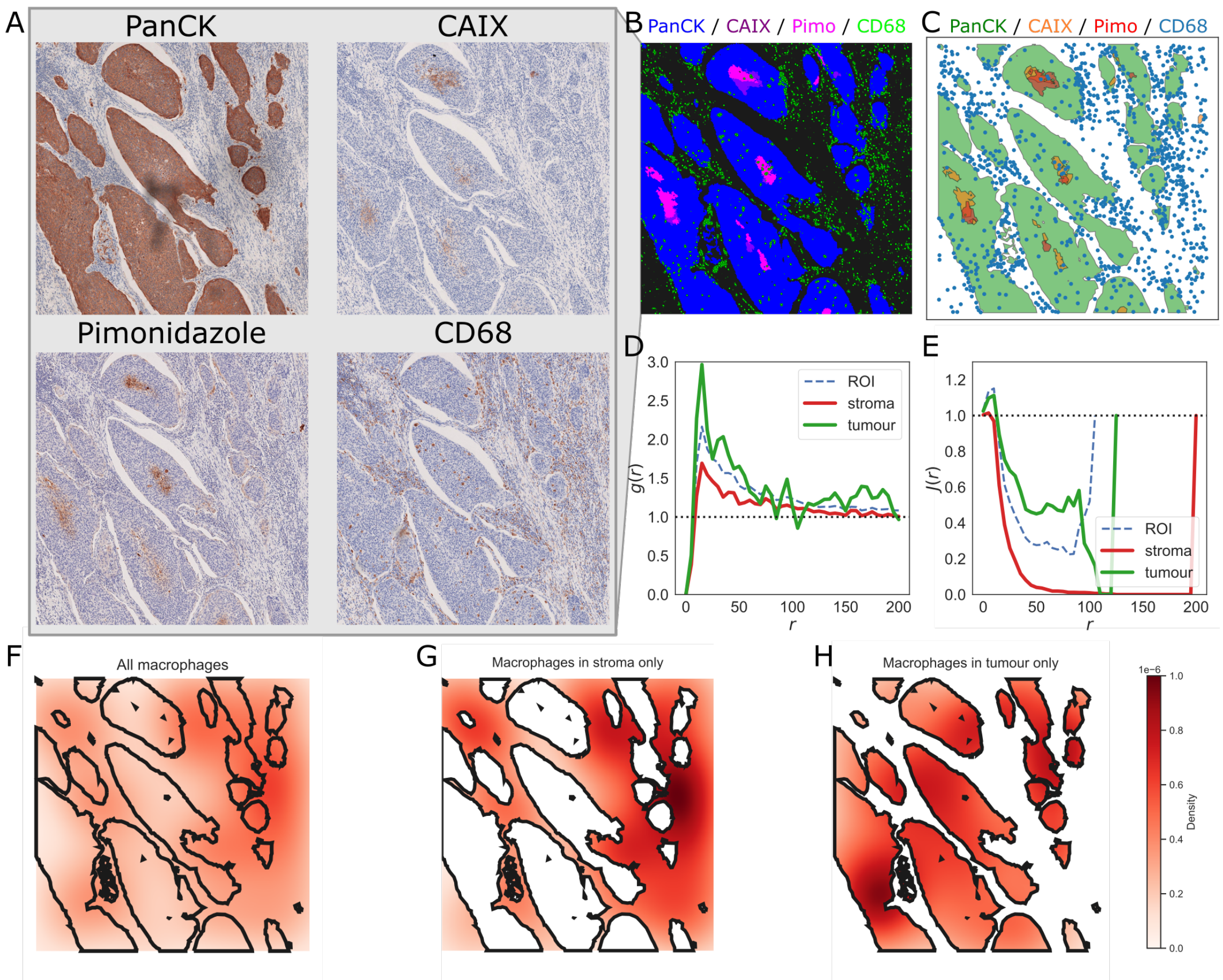

Supplementary Figure 2: **MuSpAn applied to stacked IHC data.**
A: A 1700 × 1700 pixel (1.5mm × 1.5mm) ROI from a human head and neck cancer sample, stained on consecutive slides in the tissue stack to show PanCK (tumour cells), CAIX (hypoxia), pimonidazole (severe hypoxia) and CD68 (macrophages).
B: Composite image showing the alignment of the four markers.
C: Boundaries and points from panel B shown as a MuSpAn domain.
D, E: Cross-PCFs (D) and J-function (E) describing macrophage-macrophage correlation within the domain. The dashed blue lines (ROI) show macrophage-macrophage correlation across the whole ROI. The solid green (tumour) and red (stroma) lines show macrophage-macrophage correlation, restricted to inside the PanCK regions (tumour) or outside (stroma). F: Kernel density estimation (KDE) of macrophage localisation within the ROI. Outlines of PanCK shapes are shown for reference, but not used in the estimation.
G: KDE of macrophage localisation outside of the PanCK (tumour) regions.
H: KDE of macrophage localisation within the PanCK (tumour) regions.

Supplementary Note 1.2 COVID-19 infected lung (Imaging Mass Cytometry)

We analyse a 2mm × 2mm ROI from an Imaging Mass Cytomtery (IMC) dataset of COVID-19 infected lung tissue, histologically classified as 'Organising Pneumonia' [S2] (Supplementary Fig. 3A-C). The original analysis pipeline involved three key steps: computing the QCM to identify cell pairs of interest, followed by cross-PCF and TCM analyses.

Using MuSpAn, we replicate and extend this pipeline. We first compute the QCM across all cell types (Supplementary Fig. 3D, quadrat size 100µm) and perform an adjacency permutation test (APT) using a Delaunay network with a 20µm cut-off [S5] (Supplementary Fig. 3E). We focus on two cell pairs: 'Blood vessels'-'UD structural SM' (high QCM correlation) and 'UD-structural SM'-'Myofibroblasts' (low QCM correlation). Interestingly, both pairs show positive spatial association in the APT, despite differing QCM scores.

We compute cross-PCFs and cross-K functions for both pairs (Supplementary Fig. 4A-B, Supplementary Fig. 5A-B). The 'UD structural SM'-'myofibroblast' pair shows no short-range correlation and weak anti-correlation at larger scales. To resolve the apparent contradiction in the negative QCM score, positive APT score, and neutral spatial statistics, we extend the analysis with KDEs (Supplementary Fig. 4C-D, Supplementary Fig. 5C-D), revealing that while 'UD structural SM' cells are widespread, their densest regions do not overlap with 'myofibroblast' clusters. Nearest-neighbour analysis (Supplementary Fig. 4E-F, Supplementary Fig. 5E-F) confirms that most 'myofibroblasts' are near 'UD structural SM' cells, but many 'UD structural SM' cells are distant from 'myofibroblasts'. Finally, TCMs (Supplementary Fig. 4G, Supplementary Fig. 5G) show that for this cell pair local positive correlation is confined to specific regions, balanced by areas of exclusion, explaining the neutral global PCF.

This example illustrates the need for caution when interpreting spatial statistics in isolation. The QCM suggests negative correlation due to mismatched distributions across the ROI, while the APT indicates positive interaction driven by a shared region of high cell density. The TCM helps reconcile these findings: local clustering is offset by areas rich in 'UD structural SM' cells but lacking 'myofibroblasts', resulting in a neutral global PCF. Together, these analyses show how different statistics capture distinct aspects of spatial organisation.

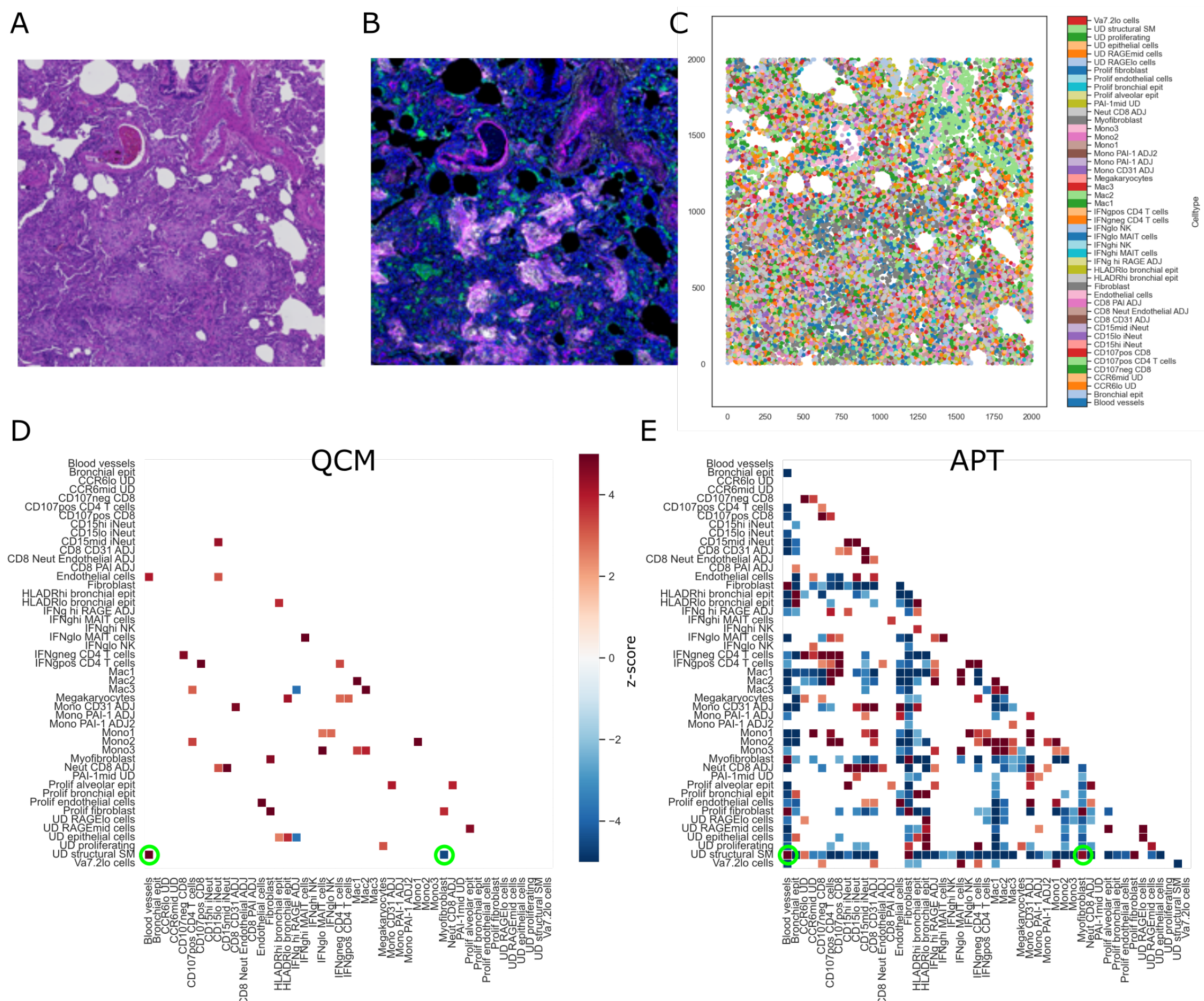

Supplementary Figure 3: **MuSpAn applied to Imaging Mass Cytometry (IMC) data.**
A: H&E staining of a 2mm × 2mm ROI from a human lung resected from a COVID-19 infected patient [S2].
B: False colour IMC staining of the same lung ROI.
C: Point cloud of cell centres from the ROI, with annotations from [S2].
D: Quadrat correlation matrix (QCM) for the cells in this ROI (square quadrats, edge length 100µm), showing statistically significant values only and highlighting the cell pairs analysed further.
E: Adjacency permutation test (APT) for the cells in this ROI (Delaunay network, maximum edge length 20µm), showing statistically significant values only and highlighting the cell pairs analysed further.

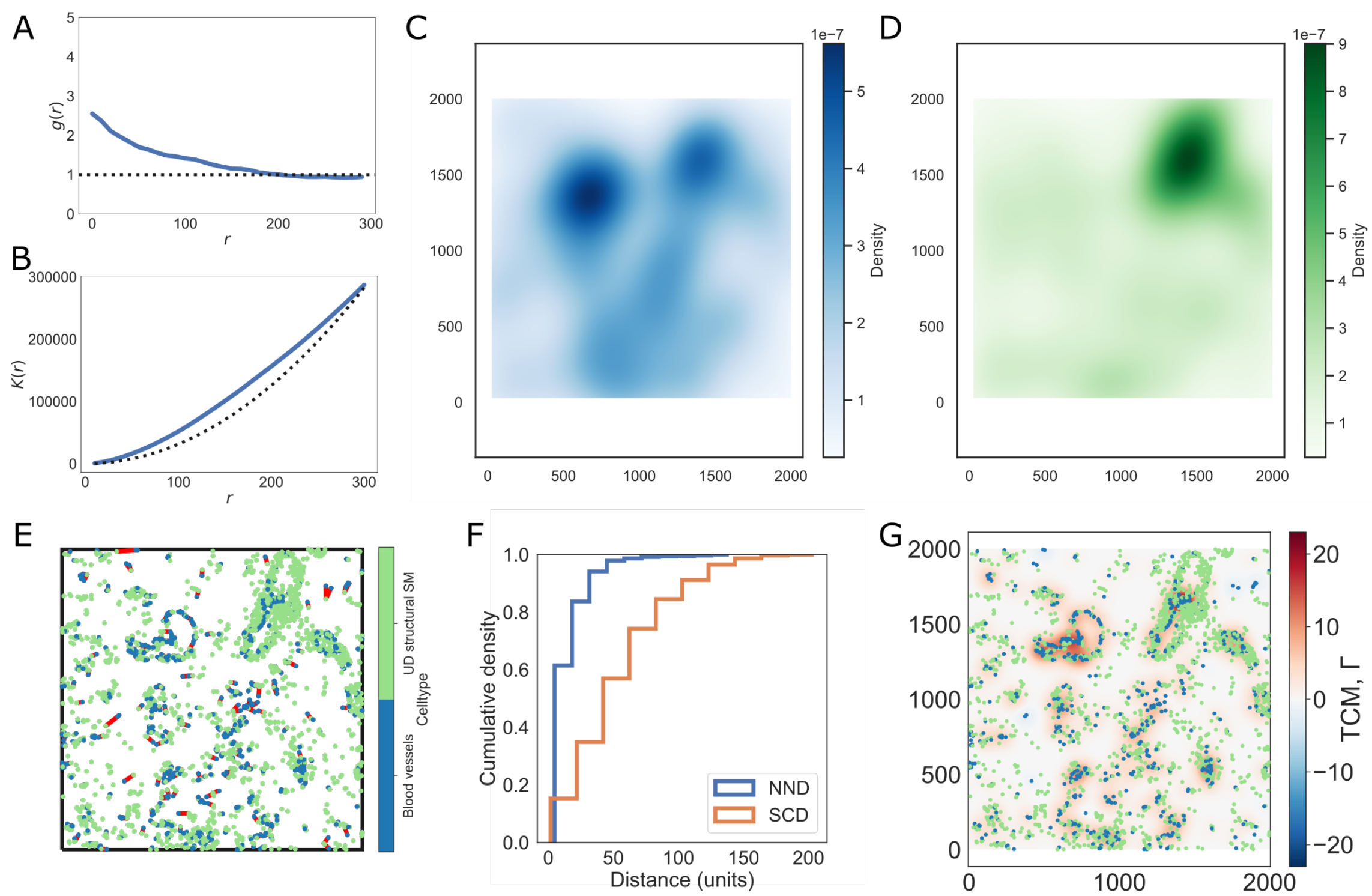

Supplementary Figure 4: **MuSpAn applied to IMC data - positive spatial correlation.** A: Cross-PCF function between blood vessels and 'UD structural SM' cells, indicating clusters of the two populations with a characteristic size of approximately 180µm.
B: Cross-K function between the two cell populations, indicating increased association at length scales up to 300µm. C: Kernel Density Estimation (KDE) of blood vessels in the ROI.
D: KDE of 'UD structural SM' cells in the ROI.
E: Nearest neighbour distances (red) from blood vessels to the nearest 'UD structural SM' cell, two populations which are highlighted as spatially correlated by the QCM in Supplementary Fig. 3D.
F: Cumulative density function of the nearest neighbour distribution (NND), compared with a null of the spherical contact distribution for blood vessels (SCD). G: Topographical Correlation Map (TCM) showing where blood vessels are locally positively or negatively associated with 'UD structural SM' cells.

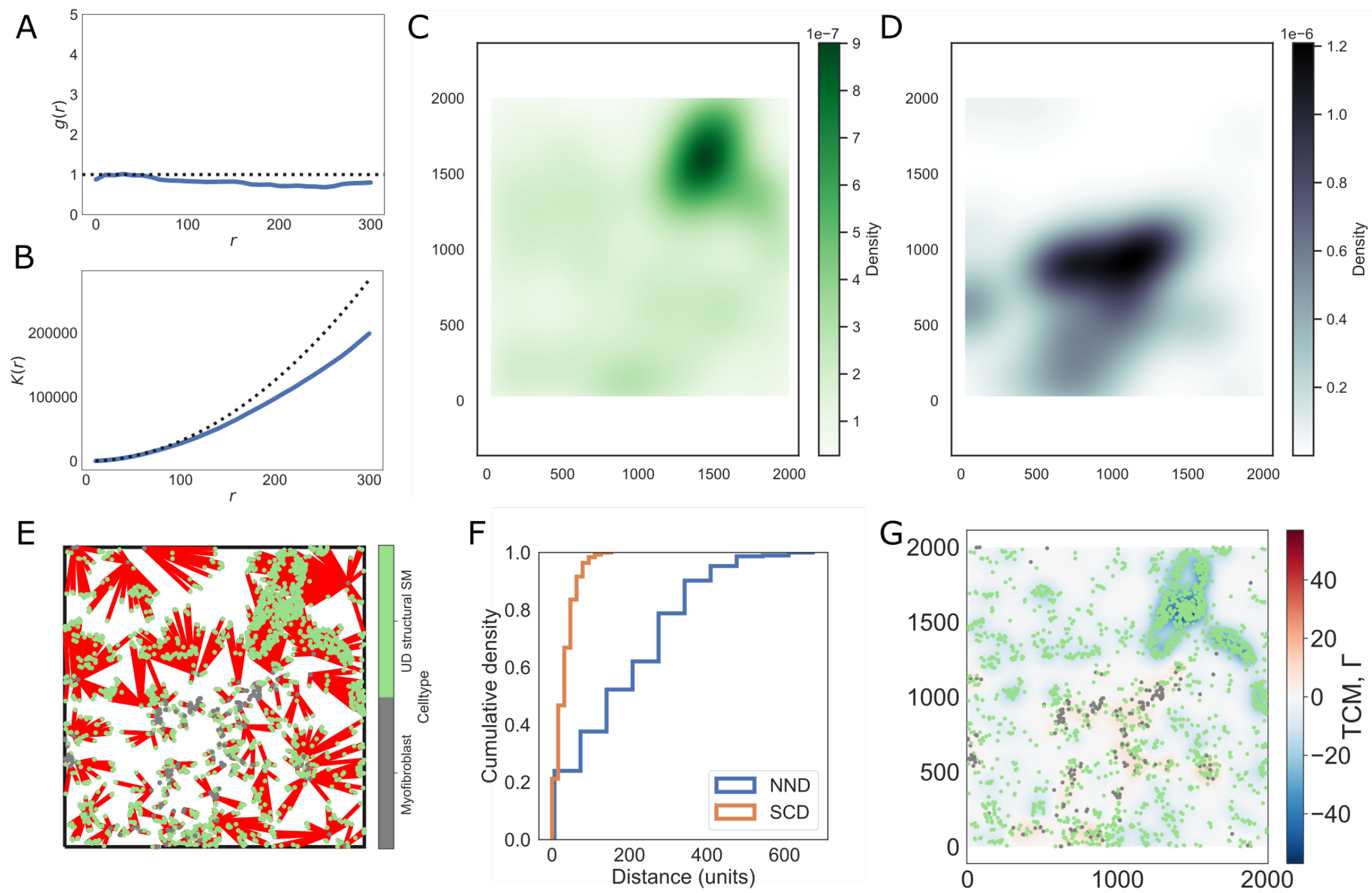

Supplementary Figure 5: **MuSpAn applied to IMC data - spatial anti-correlation.**
A: Cross-PCF between 'UD structural SM' cells and myofibroblasts, two populations which are highlighted as spatially anti-correlated by the QCM in Supplementary Fig. 3D. This indicates no strong positive or negative correlation between the populations at short length scales, and weak anti-correlation at length scales beyond 100µm.
B: Cross-K function between the two cell populations, indicating decreased association at length scales up to 300µm and beyond.
C: Kernel Density Estimation (KDE) of 'UD structural SM' cells in the ROI.
D: KDE of myofibroblasts in the ROI.
E: Nearest neighbour distances (red) from 'UD structural SM' cells to myofibroblasts.
F: Cumulative density function of the nearest neighbour distribution (NND), compared with a null of the spherical contact distribution for 'UD structural SM' cells (SCD). G: Topographical Correlation Map (TCM) showing where 'UD structural SM' cells are locally positively or negatively associated with myofibroblasts.

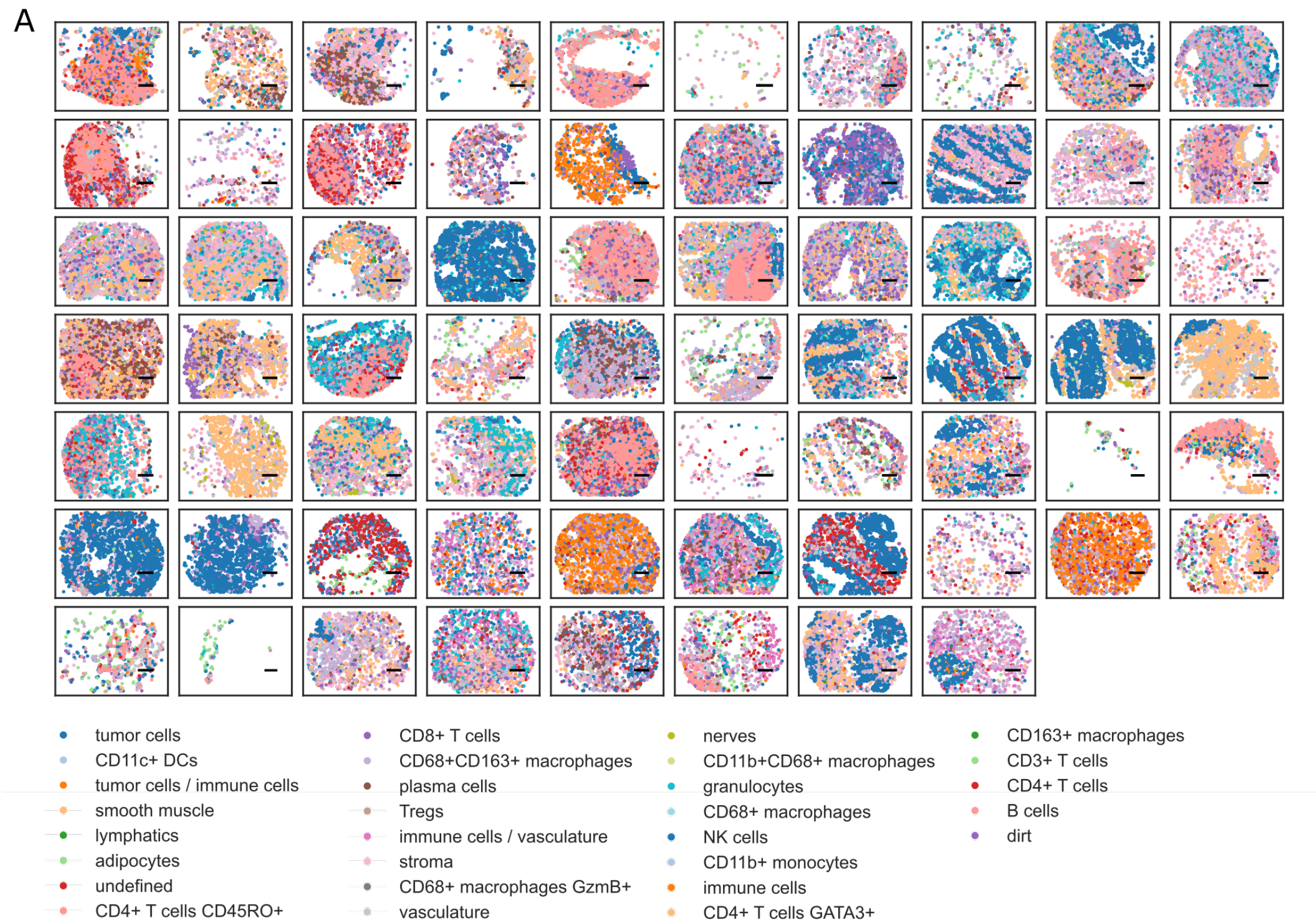

Supplementary Figure 6: **MuSpAn applied to CODEX data.**
A: MuSpAn visualisation of cell centres from the TMAs used in [S3].

Supplementary Note 1.3 CODEX - Schürch *et al.* (2020)

We use MuSpAn to reproduce and extend the analysis of CODEX tissue microarray (TMA) data in [S3]. Supplementary Fig. 6A shows a selection of 68 TMAs ('group 1', regions with "Crohn's like reaction", CLR) from this dataset. We use MuSpAn to conduct a neighbourhood enrichment study based on a k-nearest neighbours network (and assigned 10 k-means clusters using `muspan.networks.cluster_neighbourhoods()` to obtain an enrichment matrix for each cluster, Supplementary Fig. 7A). The clusters obtained from this analysis are in good qualitative agreement with those obtained by Schürch *et al.* [S3]. MuSpAn assigned clusters are shown in Supplementary Fig. 7B and D, and the corresponding cluster assignments for the same regions from [S3] are shown in Supplementary Fig. 7C and E (note that since neighbourhood cluster assignment is conducted on all samples simultaneously, not every cluster is represented in every TMA).

We extend this analysis by merging adjacent Voronoi cells in each TMA which share the same label to form contiguous regions within which all cells belong to the same cellular neighbourhood. This facilitates applying MuSpAn operations directly to the cellular neighbourhoods, treating each contiguous region as a separate shape. We conduct an adjacency permutation test (APT) on each TMA, obtaining a standardised effect size (SES, z-score) describing whether any two types of

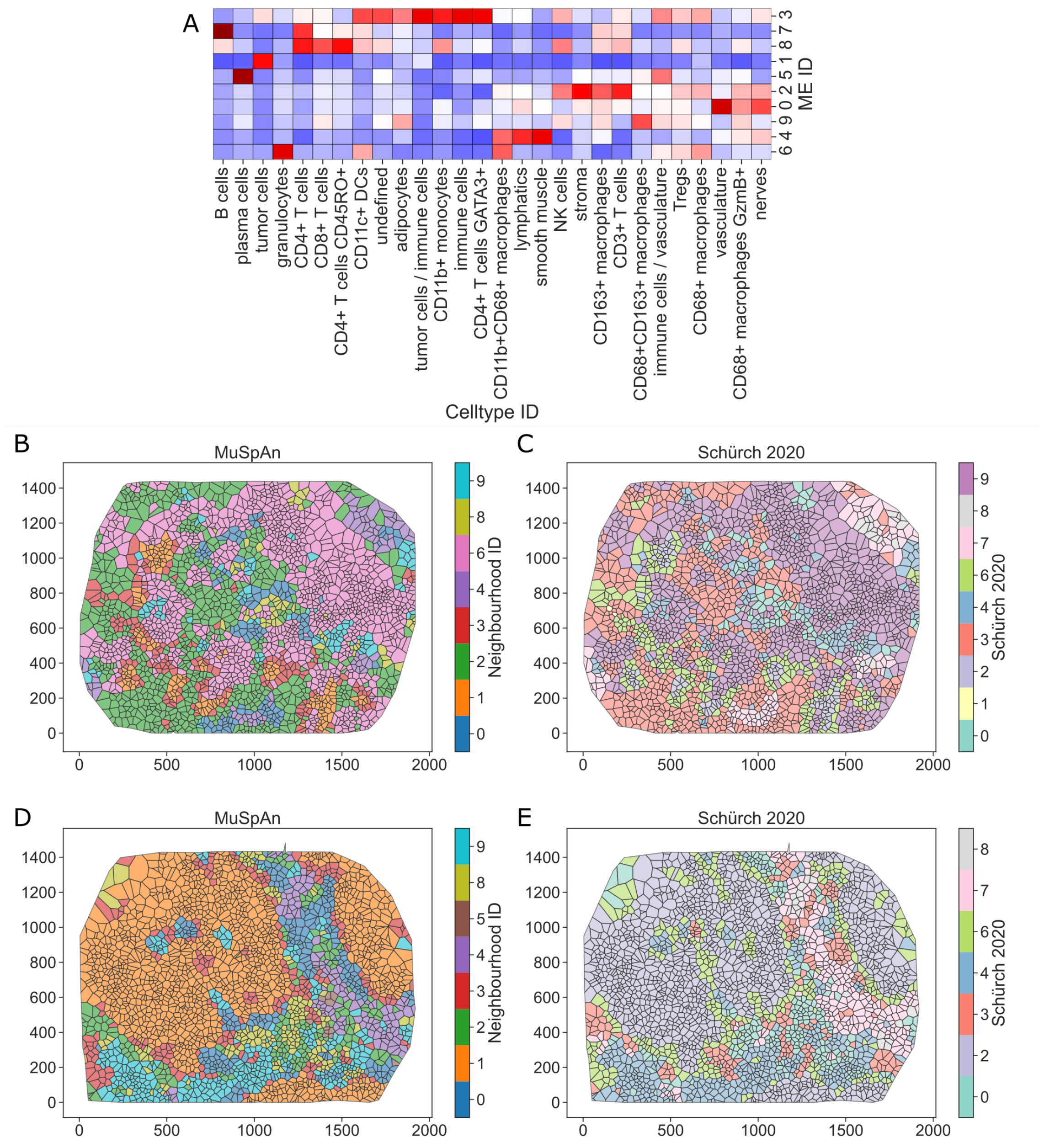

Supplementary Figure 7: **MuSpAn applied to CODEX data.**

A: A microenvironment enrichment matrix showing the celltypes which are enriched in each of the 10 microenvironment clusters (ME ID 0-9) detected in a MuSpAn reproduction of the Schürch *et al.* pipeline.

B-E: Voronoi representations of cell data from two TMAs (B/D and C/E), coloured according to the MuSpAn assigned cellular neighbourhoods (B, D) and to the Schürch *et al.* assigned neighbourhoods (C, E).

neighbourhood are found in direct contact more or less frequently than would be expected under random labelling. For a given pair of neighbourhoods, we compare the distribution of SES (z-score) in 'group 1' (Crohn's like reaction, CLR) and 'group 2' (diffuse inflammatory infiltration, DII) TMAs. Supplementary Fig. 8A shows the SES for interactions between Neighbourhood 4 and Neighbourhood 8 in each TMA, which are significantly higher in the DII regions than the CLR ($p \approx 0.0013$, unequal variance t-test). Similar tests applied to all combinations of Neighbourhoods identify a small number of statistically significant differences in the neighbourhood connectivity between CLR and DII (Supplementary Fig. 8B).

To better interpret these scores, we show the four TMAs from group 1 (CLR) with the lowest z-scores, and those from group 2 (DII) with the highest (Supplementary Fig. 8C). TMAs with low z-scores (CLR) generally have small neighbourhoods of types 4 and 8, which are rarely in direct contact. In contrast, the type 4 and 8 neighbourhoods in group 2 (DII) are much larger, and are found frequently in direct contact with one another. These neighbourhoods approximately correspond to the 'T cell enriched' (neighbourhood 8) and 'smooth muscle' (neighbourhood 4) cellular neighbourhoods described in [S3], suggesting that there is a significant difference between the localisation of T cell infiltration into stromal regions between CLR and DII regions.

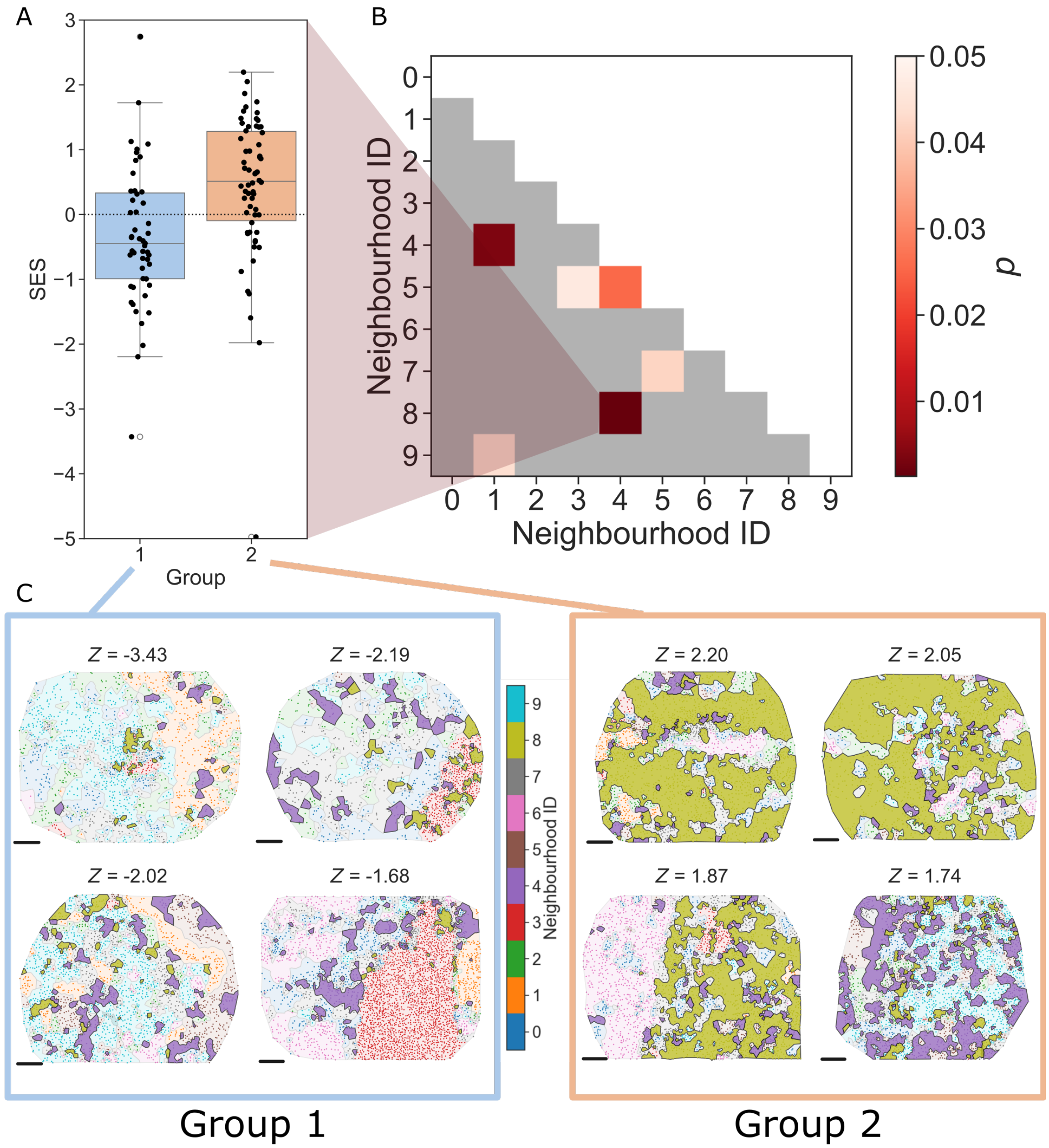

Supplementary Figure 8: **Neighbourhood contacts vary between CLR and DII TMAs** A: Standardised effect sizes (z-scores) for adjacency permutation test (APT) interactions between Neighbourhoods 4 and 8, within each TMA from group 1 (CLR) and group 2 (DII) ($p = 0.0013$, unequal variance t-test). B: p-values showing which neighbourhood-neighbourhood interactions have significantly different APT scores betwen group 1 and group 2 (unequal variance t-tests). C: The four TMAs with the lowest SES (group 1) or highest SES (group 2). Regions corresponding to neighbourhoods 4 (purple) and 8 (olive) are highlighted. In group 1, these neighbourhoods are small and rarely in direct contact. In group 2, the neighbourhoods are much larger and frequently share boundaries. Scalebars are 200 pixels.

Supplementary Note 2 Biologically-driven pipeline generation with MuSpAn

The examples in the main text (Fig. 6) and Supplementary Note 1 illustrate how MuSpAn enables the construction of spatial analysis pipelines tailored to specific biological hypotheses. Each pipeline applies distinct statistical methods to interrogate different aspects of spatial organisation. Crucially, spatial analysis is not a one-size-fits-all process - methods suitable for investigating one hypothesis may be inappropriate for another.

Effective pipeline design hinges on selecting statistics that capture the biological feature of interest from complementary perspectives. For clarity, we demonstrate how different mathematical approaches highlight distinct spatial features with reference to a simple synthetic dataset in which blue and orange cells form a ring, surrounded by randomly placed green points (Supplementary Fig. 9). We focus on three commonly-arising spatial features of a dataset: cell-cell contact (Supplementary Fig. 9A), description of local cellular neighbourhoods (Supplementary Fig. 9B), and characterisation of emergent tissue architectural features (Supplementary Fig. 9C). We emphasise that these examples represent just a subset of the perspectives that could analysed with MuSpAn, which is designed to make bespoke pipeline development simple. Our aim here is not to prescribe a fixed set of analyses, but to show how MuSpAn provides a flexible framework adaptable to diverse biological contexts.

Dataset

The synthetic dataset used in Supplementary Fig. 9 is available within MuSpAn as the *'Synthetic-Points-Architecture'* dataset, and can be easily loaded via the command `muspan.datasets.load_example_domain(`*'Synthetic-Points-Architecture'*`)`. The panels shown represent a subset of the full dataset centred around a ring of orange and blue points, with green points randomly placed throughout the domain; for full details on the dataset, see Figure 1 of [S6]; code for generating the panels shown is available alongside this paper.

Supplementary Note 2.1 Cell-cell contact

Supplementary Fig. 9A illustrates three complementary approaches for analysing cell-cell contact:

- **Adjacency networks** provide a global view of direct cell-cell interaction that considers all cells simultaneously. They can be constructed from segmentation masks, or inferred from cell centroids using a Delaunay triangulation. Network edges may be weighted by metrics such as intercellular distance, and statistical tests like adjacency permutation [S7] can assess contact patterns across multiple cell types simultaneously.
- **Nearest neighbour analysis** instead focusses on pairwise spatial relationships between specific cell types. For example, measuring the distance from each green cell to the nearest blue cell yields a distribution that can be tested for spatial association using metrics like the Average Nearest Neighbour Index [S8]. This approach is particularly useful when it is hypothesised that direct contact is not required, but proximity is biologically meaningful.
- **Local indicators of spatial association (LISA)** offer a spatially resolved view of co-occurrence. The topographical correlation map (TCM) [S9], for instance, highlights regions where green cells are locally enriched (red) or depleted (blue) relative to the blue cell population. MuSpAn supports several LISA variants, including the Local Getis-Ord statistic (Fig. 5I).

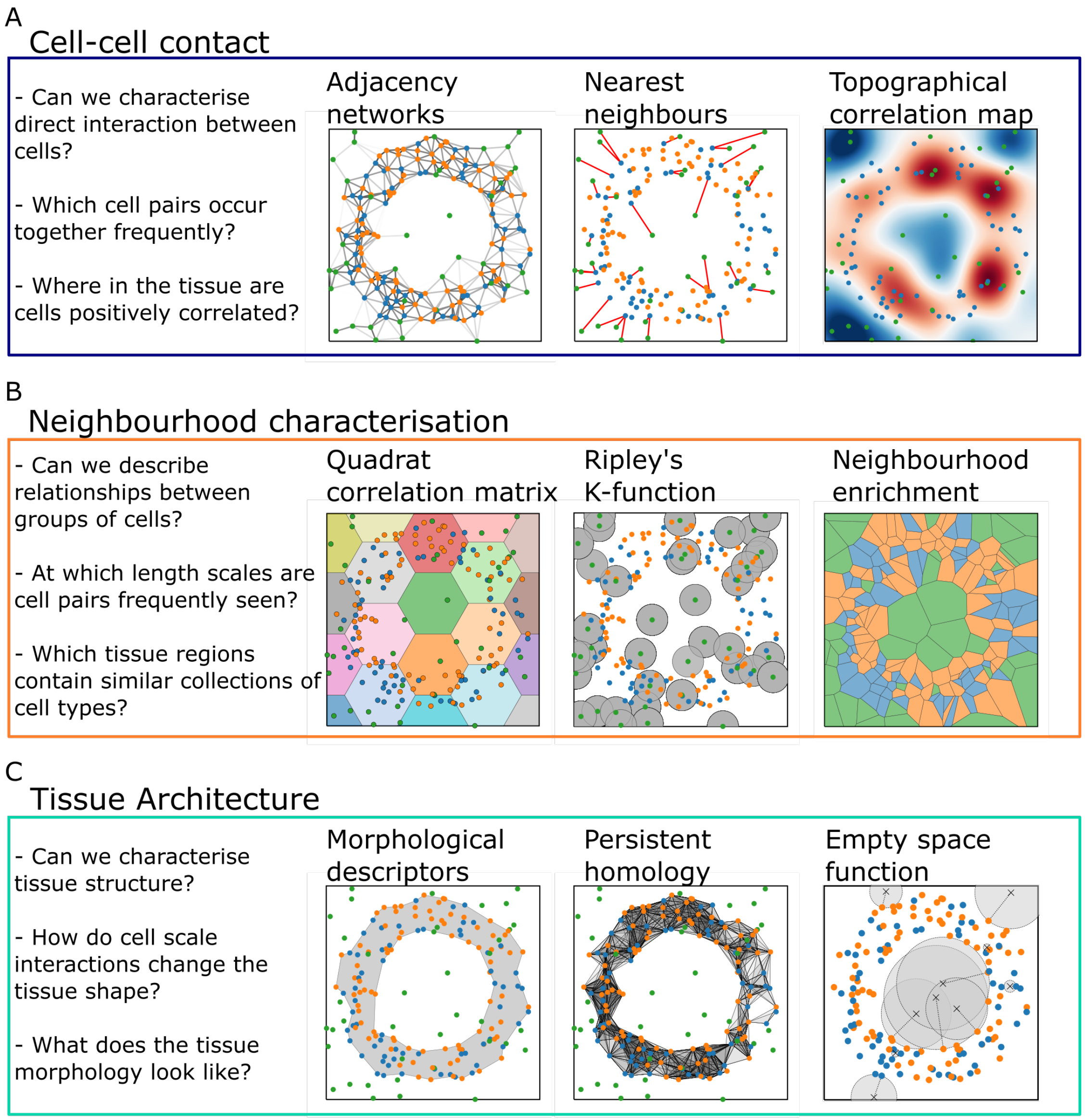

Supplementary Figure 9: **Biologically-driven pipeline development using MuSpAn**
Examples of how complementary mathematical methods can be combined to characterise different biological features. In each example we use the same illustrative points, and schematise how the methods considered use different aspects of the data. This synthetic dataset contains three cell types: blue and orange cells form a circle, and green cells are randomly distributed.
A: Cell-cell contact could be described using adjacency networks, nearest neighbour approaches, and topographical correlation maps.
B: Neighbourhood characterisation can be conducted using region-based approaches such as the QCM, spatial statistics like Ripley's K function, and neighbourhood enrichment scores.
C: Tissue architecture can be described using standard morphological descriptors (such as area/perimeter, circularity, convexity), persistent homology, and the empty-space function.

Supplementary Note 2.2 Neighbourhood characterisation

Supplementary Fig. 9B presents three strategies for describing local cellular neighbourhoods:

- **Quadrat based methods** divide the ROI into fixed-size tiles, aggregating spatial data within each. Metrics such as the quadrat correlation matrix (QCM) and Morisita-Horn index quantify label diversity and similarity across tiles [S10, S11], offering a coarse-grained view of spatial heterogeneity.
- **Spatial statistics** can examine the local environment around individual cells. For example, Ripley's cross-K function measures the spatial co-occurrence of two cell types within a defined radius [S12]. This approach captures average neighbourhood properties and can be extended to analyse interactions among three or more cell types [S9].
- **Region-based enrichment methods** define neighbourhoods based on locally dominant cell types. In the schematic, the domain is partitioned using Voronoi tessellation; the cellular composition within each region can then be characterised using, for example, k-nearest neighbour networks [S3]. MuSpAn includes several alternative methods for identifying and analysing spatially enriched zones.

Supplementary Note 2.3 Tissue architecture

To analyse multicellular structures at larger scales, Supplementary Fig. 9C shows three complementary approaches:

- **Morphological descriptors** quantify the shape of defined structures. Using α-shapes [S13], cell locations can be converted into explicit boundaries, enabling measurement of features such as area, circularity, and convexity (see Fig. 2).
- **Persistent homology (PH)** captures topological features like loops or connected components of point datasets when connected at different length scales, without requiring predefined boundaries. In the schematic, a Vietoris-Rips complex constructed from blue and orange cell positions identifies the ring structure. PH quantifies the persistence of such structures across spatial scales, offering a robust method for detecting architectural motifs.
- **Spatial statistics** including the empty space function (spherical contact distribution) provide a statistical view of voids within the ROI [S1]. Unlike nearest neighbour functions, which measure distances between cells, these functions assess the distance from random points to the nearest cell, characterising the size and distribution of empty regions.

Supplementary Note 3 Available quantitative tools

Table 1 contains a list of the data analysis methods available within MuSpAn v1.2.0; since the module is under continual development, subsequent versions contain a wider array of features. For further details about how each method works, we refer the interested reader to the citations in Table 1, or to the documentation available at https://docs.muspan.co.uk/ where quantitative tools available in later versions can be found.

Supplementary Table 1: A summary of the data analysis methods available within each module of MuSpAn v1.2.0, together with a brief summary of their purpose. For further details about these methods, please see the references provided.

**Method name** (`MuSpAn Module`)	**Summary**	**References**
Label Count (`summary_Statistics`)	Number of objects with a given label, within a given region .	-
Label Density (`summary_Statistics`)	Count, divided by region area.	[S2, S14]
Label Entropy (`summary_Statistics`)	Shannon entropy of a specified label.	[S11, S15]
Generate Distribution (`distribution`)	Convert point data into a distribution using general kernel.	-
Kernel Density Estimation (`distribution`)	Convert point data into a probability density distribution using a Gaussian kernel function.	[S16]
Kullback-Leibler Divergence (`distribution`)	Measures the similarity between two distributions using relative entropy.	[S17]
Wasserstein distance (`distribution`)	Measures the distance between two distributions.	[S6, S18]
Summarise Distribution (`distribution`)	Computes the mean and standard deviational ellipses of spatial point data.	-
Area (`geometry`)	Area of a shape.	-
Circularity (`geometry`)	Circularity of a shape.	-
Convexity (`geometry`)	Convexity of a shape (ratio of the area to the area of its convex hull)	-
Perimeter (`geometry`)	Perimeter of a shape.	-
Principle Axis (`geometry`)	Computes the angle and major axis of a polygon relative to the x-axis.	-

**Method name** (`MuSpAn Module`)	**Summary**	**References**
Adjacency Permutation Test (`networks`)	Test the correlation between adjacent labels in a network by randomly permuting them (also referred to as 'neighbourhood enrichment').	[S5, S7]
Alpha Index (`networks`)	A measure of connectivity which evaluates the number of cycles in a graph in comparison with the maximum number of cycles. (≈ 0 for a Tree, ≈ 1 for a maximal planar graph).	[S19, S20]
Betweenness Centrality (`networks`)	The sum of the fraction of all shortest paths that pass through each node.	[S20, S21]
Closeness Centrality (`networks`)	The average shortest distance from each node to all other reachable nodes.	[S20, S21]
Cluster Neighbourhoods (`networks`)	Cluster similar neighbourhoods based on the enrichment of labels within the neighbourhoods.	[S3, S22]
Community Detection (`networks`)	Perform community detection based on node connectivity.	[S23]
Compactness (`networks`)	Index in the range $[0, 1]$ that relates the area of a network to the length of its edges.	[S20, S21]
Cyclomatic Number (`networks`)	The number of elementary cycles within a graph.	[S20]
Edge Orientation Distribution (`networks`)	Compute the (symmetric) distribution of edge orientations.	[S20]
Gamma Index (`networks`)	A measure of connectivity that considers the relationship between the number of observed links and the number of possible links.	[S19, S20]
Network Generation (`networks`)	Generate a network based on MuSpAn objects. Available methods include Delaunay, k-Nearest Neighbours, and Proximity networks.	[S3, S20, S24–S26]
Katz Centrality (`networks`)	The influence of a node in a network, determined by considering the total number of walks, weighted by their lengths, that connect the node to all other nodes..	[S20]
k-Hop Neighbourhood (`Networks`)	Find the k-hop neighbourhood of a set of source objects	-
k-Nearest Neighbour Label Count (`networks`)	Count the occurrences of each label within k steps from a set of source objects	-
Laplacian Centrality (`networks`)	A measure of node connectivity in a graph	-

**Method name** (`MuSpAn Module`)	**Summary**	**References**
Network Distance (`networks`)	Measure the similarity of two networks, using a range of available distance metrics.	[S27]
Node Degree (`networks`)	Compute the degree of each node.	-
Generate Quadrats (`region-based`)	Create a mesh of quadrats covering the requested space.	-
Generate Hexgrid (`region-based`)	Create a mesh of regular hexagons covering the requested space.	-
Morisita Horn Index (`region-based`)	A measure of the similarity of observations of a label within a set of regions.	[S10, S11]
Quadrat Correlation Matrix (`region-based`)	Describe correlations between the counts of different cell types within a set of regions.	[S2, S28]
Average Nearest Neighbour Index (`spatial_statistics`)	A measure of spatial aggregation based on the average distance between pairs of objects.	[S8, S15]
Cross Ripley's K Function (`spatial_statistics`)	A measure of (cumulative) spatial aggregation between pairs of objects across length scales.	[S29]
Cross L Function (`spatial_statistics`)	A normalised version of Ripley's K-Function.	[S29]
Cross Pair Correlation Function (`spatial_statistics`)	A measure of spatial aggregation between pairs of objects across length scales.	[S9, S29]
Getis Ord Statistic (`spatial_statistics`)	A measure of spatial autocorrelation that identifies hot- and cold-spots in the distribution of a continuous label.	[S30–S32]
J-Function (`spatial_statistics`)	A test that identifes clustering and dispersion in point patterns. Relates the Spherical Contact Distribution and the Nearest Neighbour Distribution.	[S1, S33]
Moran's I (`spatial_statistics`)	Calculate both local and global Moran's I statistic within the domain. The local Moran's I statistic quantifies the spatial autocorrelation for each object in the population. The global Moran's I statistic quantifies the spatial autocorrelation for the entire population.	[S15, S26, S31]
Nearest Neighbour Distribution (`spatial_statistics`)	Calculate the distribution of distances between the closest objects in two populations.	[S22]
Spherical Contact Distribution (`spatial_statistics`)	Calculate the distance from randomly sampled points to the closest objects in a population. Also referred to as the 'Empty Space Distribution'.	[S1]

**Method name** (MuSpAn Module)	**Summary**	**References**
Topographical Correlation Map (spatial_statistics)	A spatially-resolved, quantitative summary of colocalisation between two point populations across a region of interest.	[S2, S9]
Weighted Pair Correlation Function (spatial_statistics)	An extension of the cross PCF which accounts for continuous labels.	[S9, S34]
Level Set Filtration (topology)	Calculate the level set filtration of a distribution or image.	[S6, S35]
Vietoris-Rips Filtration (topology)	Calculate the Vietoris-Rips filtration of a set of points.	[S35]
Vectorise Persistence (topology)	Convert persistence diagram into a vector for subsequent analysis.	[S36]
Cluster Data (helpers)	Wrapper for clustering methods implemented in scikit-learn.	[S37]
Crop Domain (helpers)	Return a new domain (or domains) based on a set of new boundary shapes (or label values).	-
Combine Shapes (shape_operations)	Combine two shapes in the domain using a specified operation.	-
Return Difference of Shapes (shape_operations)	Compute the difference between two shapes.	-
Return Intersection of Shapes (shape_operations)	Compute the intersection of two shapes.	-
Return Union of Shapes (shape_operations)	Compute the union of two shapes.	-

Supplementary Note 4 Comparison of MuSpAn with alternative tools

This supplementary note compares MuSpAn with existing software packages for analysis of spatial data.

Supplementary Note 4.1 Overview - comparison between MuSpAn and alternative tools

Here, we emphasise here the key design and application differences between MuSpAn and alternative packages often used in working with spatial data. Most notably, we emphasise that MuSpAn is a toolbox designed to provide quantitative spatial analysis of the spatial structure of generic spatial datasets in biology, while many other commonly used packages provide different functionality. In particular, we note that MuSpAn is not intended to perform all possible operations that may be of interest when working with spatial biology, with important operations such as differential gene expression analyses or cell type deconvolution falling outside of its remit.

Table 2 contains a brief comparison of several commonly used packages.

In particular, MuSpAn is designed to work with data that has been pre-processed using any other commonly used package. The design philosophy is to take in generic spatial data - that is, data with coordinates and associated metadata labels (e.g., cell types assigned by Seurat or ScanPy). This information can be derived from an almost limitless number of pre-processing pipelines; rather than restricting inputs to one ecosystem, MuSpAn assumes that these values can be extracted from any other data format using standard methods available within Python. For convenience, we also provide import functionality for common segmentation tools via the `muspan.io` module.

The key difference between MuSpAn and other packages designed for quantitative description of structure in spatial data (as opposed to packages working with other aspects of spatial omics workflows) is its emphasis on flexibility, mathematical breadth, and capacity for multiscale descriptive statistics. MuSpAn permits users to flexibly construct pipelines tailored to their own biological hypotheses 'on the fly' (see Supplementary Note 1 and Supplementary Note 2).

Where biological concepts such as 'spatial domains' or 'cellular neighbourhoods' do not have agreed on statistical definitions, MuSpAn permits users the flexibility to calculate these via their preferred route (see Fig. 5 for an example of this, where a variation of 'cellular neighbourhoods' are calculated using a Delaunay graph instead of the more common k-nearest neighbour graph). These derived objects can also be treated as primary objects for spatial analysis, ensuring that spatial patterning of cellular neighbourhoods (or other derived objects) can be identified as easily as spatial patterning of cell locations (see Supplementary Note 1.3 for an example of this).

Supplementary Table 2: Comparison of MuSpAn with alternative packages for analysis of spatial imaging data

Package	Package Aims	Strengths	Spatial limitations	Ref.
MuSpAn	Dedicated Python toolbox for quantitative multiscale description of spatial patterns and structures	Rich set of mathematical frameworks adds breadth and robustness (spatial statistics, topology, geometry, networks,...). Agnostic to imaging technology and preprocessing. Multiscale by design, and applicable to arbitrary spatial objects (e.g., cells, transcripts, cell neighbourhoods derived from other analyses). Flexible workflows. Focus on ensuring quantitative accuracy (boundary corrections, statistically robust spatial metrics) and cutting-edge mathematical methods.	No tools for pre-processing, segmentation, clustering or phenotype assignment.	-
Seurat	R toolkit for single-cell and spatial transcriptomics using scRNA-seq workflows	Mature ecosystem; strong integration with other R packages; widely used implementation of phenotype assignment methods.	Not focussed on spatial quantification - spatial analysis is largely an extension of non-spatial scRNA-seq workflows to apply to spatial data.	[S38]
ScanPy	Python toolkit for single-cell gene expression data analysis	Highly scalable; Widely-used and established framework for non-spatial data, integrated with scverse. Strong preprocessing and clustering tools for phenotype assignment.	Not designed for spatial analysis - most spatial functionality delegated to extension packages (see SquidPy below).	[S39]
SquidPy	Python framework for graph-based spatial quantification of AnnData.	Graph-based approach is fast; established framework (integrated with scverse, builds on ScanPy). Range of graph-based statistics available.	No spatial edge corrections; graph-based approach means that interpretation is tightly coupled to the choice of neighbourhood graph. Requires AnnData input. Limited support for non-graph metrics or explicit multiscale/geometric analysis.	[S26]
Giotto Suite	'End to end' platform for spatial omics visualisation and analysis	Rich tools for image visualisation filtering, normalising and clustering gene expression data.	Spatial quantification is largely linked to expression-driven workflows and spatial graphs; limited support for alternative mathematical frameworks (spatial statistics, geometry/topology-based analyses). Available quantification methods are primarily applied to cell or spot locations, with limited support for analysis of derived spatial objects (e.g. higher-order neighbourhoods or regions)	[S40]

Supplementary Note 4.2 Quantitiative comparison between MuSpAn and SquidPy

The most directly comparable package to MuSpAn is SquidPy, which is also a Python package focussed on providing metrics for spatial quantification, and provides several methods which overlap with some of those available within MuSpAn. Here, we present a brief quantitative comparison between some overlapping methods.

The subsequent tests were conducted using the most up to date versions of the software packages at time of writing this supplementary note (MuSpAn v1.2.5, SquidPy v1.8.1), in a clean virtual environment. Since SquidPy requires AnnData objects to conduct analyses, we chose to use an example dataset provided by SquidPy rather than converting the datasets used within this paper into AnnData objects.

We considered the default Visium dataset available within SquidPy, available using the command `sq.datasets.visium_hne_dataset()`. This returns an AnnData object containing the genetic profile of each spot, together with pre-assigned clusters.

Supplementary Note 4.2.1 Visualisation

While not a spatial metric, we have included a brief comparison of the default visualisations within MuSpAn (Supplementary Fig. 10A) and SquidPy (Supplementary Fig. 10B). Key differences are: MuSpAn plots the boundary of the region which will be used for boundary correction for spatial statistics; SquidPy overlays the spots on top of the corresponding slide image; MuSpAn places the origin at the lower left corner of the axes whereas SquidPy places the origin in the upper left corner.

Supplementary Note 4.2.2 Moran's I

While most spatial metrics within MuSpAn are not available within SquidPy, Moran's I is available in each. Both packages rely on networks to define the geometry between spots when computing Moran's I. We follow the calculations described at https://squidpy.readthedocs.io/en/latest/notebooks/examples/graph/compute_moran.html for SquidPy (k-nearest neighbours graph), and use a Delaunay triangulation for MuSpAn (the default choice). Both packages allow the underlying network to be changes for this analysis.

The output of the Moran's I calculations differs between the packages: SquidPy reports I values, whereas MuSpAn reports normalised z scores (since interpretation of an I value depends on the population size and distribution). We have converted the SquidPy output to the relevant z score to facilitate comparison (Supplementary Fig. 10C). We calculate Moran's I for the top 100 most variable genes within the panel, and compare the resulting z-scores (Supplementary Fig. 10C). There is good agreement between the packages, with small discrepancies in z-scores due to differences in the random distributions sampled to generate the z-scores and differences in definitions of spot connectivity induced by the different network choices.

Supplementary Note 4.2.3 Ripley's L-function

Both packages include functions derived from Ripley's K function; we compare computations of Ripley's L-function as a representative example of these. Under CSR, the L-function converges to the line $L(r) = r$ (solid gray line, Supplementary Fig. 10D). SquidPy does not permit calculations of cross-K or cross-L type functions, instead implementing Ripley's functions only for autocorrelation (i.e., to identify clustering or exclusion of a single labelled population). We therefore calculate the

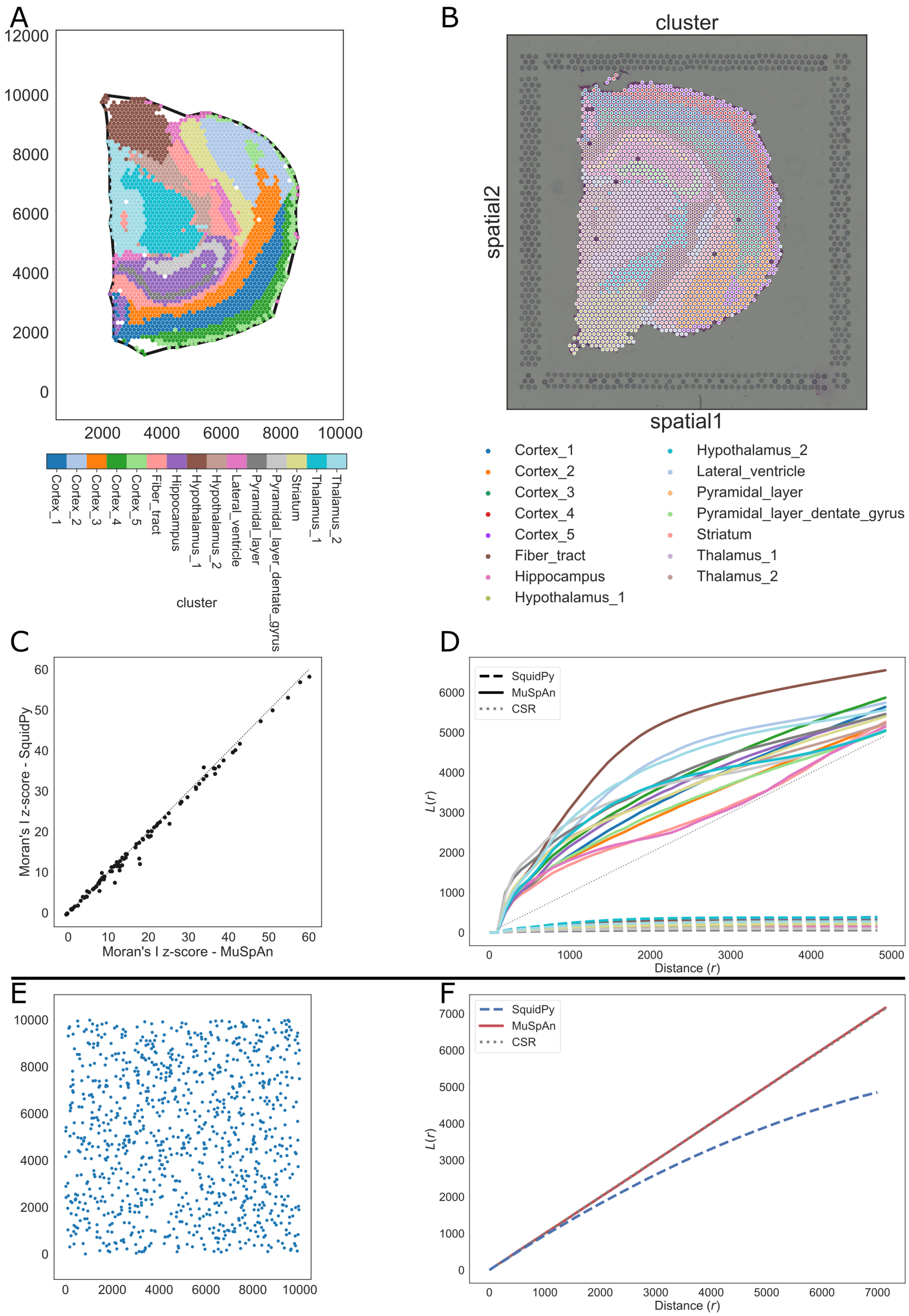

Supplementary Figure 10: **Comparison of MuSpAn and SquidPy**
Comparison of selected visualisation and statistical tools between MuSpAn and SquidPy on a Visium dataset.

A: MuSpAn visualisation of the Visium data, coloured by Cluster number. MuSpAn shows the domain boundary to clarify which area will be used for spatial statistical calculations. By default, MuSpAn shows data with the origin at the bottom left of the axis.

B: SquidPy visualisation of the same Visium dataset, coloured by cluster. SquidPy shows the spots overlaid on the background reference H&E image.

Supplementary Figure 10 *(previous page)*:
C: Comparison of Moran's I z-scores from SquidPy and MuSpAn for the 100 most highly variable genes in the dataset (note that SquidPy returns I rather than z; we have converted to z-scores to aid comparison). We note that minor discrepancies are expected due to different random permutations, and differences in the spatial structure assumed (SquidPy's analysis relies on an underlying k-nearest neighbours graph; MuSpAn by default uses a Delaunay triangulation to define adjacency between the spots for Moran's I calculation.).
D: Comparison of autocorrelation L-functions between SquidPy and MuSpAn. The dotted grey diagonal line shows the expected L-function under CSR; solid lines are from MuSpAn, dashed lines are from SquidPy. Line colours match the cluster colours in panel A. SquidPy's L-functions reflect strong exclusion between clusters, while MuSpAn's describe clustering of spots in the same cluster, more accurately reflecting the strong spatial colocalisation seen in panels A and B. The difference is likely an error in SquidPy's normalisation of the L-function, possibly caused by normalising values against the total number of spots rather than the number of spots of the population of interest.
E: Location of 1000 randomly sampled points used to assess the impact of boundary conditions on the L-function (panel F).
F: With a single population, SquidPy's normalisation term is unbiased and the L-function correctly tracks the expected function under CSR for small r. For large r, the deviation is consistent with a lack of boundary correction in calculating the L-function. MuSpAn correctly identifies the lack of spatial correlation at all length scales.

autocorrelation L-function, $L_{c,c}(r)$, for each cluster label c, in both MuSpAn and SquidPy. The resulting functions are plotted in Supplementary Fig. 10D, coloured according to the cluster labels in panel A.

Surprisingly, there is a marked difference between the outputs: the MuSpAn L-functions (solid lines) all lie above the line $L(r) = r$ which corresponds to the expected value of L under complete spatial randomness, suggesting strong spatial aggregation for all clusters. In contrast, the equivalent L-functions calculated using SquidPy lie well below the line $L(r) = r$, suggesting strong regularity/exclusion in the point patterns. Visual inspection of Supplementary Fig. 10A/B shows clear aggregates of spots for each cluster, which is consistent with the MuSpAn analysis, but not the SquidPy analysis.

According to SquidPy's documentation, SquidPy may underestimate Ripley's functions for large r values because they do not implement edge correction terms for points close to the domain boundary. Hypothesising that this might account for the large variation between MuSpAn and SquidPy in Supplementary Fig. 10D, we repeated the L-function analysis on a dataset with 1000 randomly placed points inside a square of edge length 10,000 (Supplementary Fig. 10E), generated according to complete spatial randomness.

Since MuSpAn includes edge corrections for points near the boundary, for this dataset we expect it to produce an L-function in close agreement with the theoretically expected result of $L(r) = r$ for all r (Supplementary Fig. 10F). As expected, SquidPy's L-function is in good agreement with the theory for small r ($r \lessapprox 1000$) but diverges for larger r as boundary effects become more important. The results in panel F suggest that the more extreme mismatches observed in Supplementary Fig. 10D are not due to errors in boundary correction. We hypothesise that the mismatch is related to an open issue (at time of writing) relating to inconsistent normalisation of Ripley's functions in

SquidPy when multiple point populations are present (https://github.com/scverse/squidpy/issues/813).

Supplementary Note 5 Further information on spatial analysis methods

Below, we describe the spatial analysis methods used to generate Figs. 2-5 of the main text. The methods are organised to mirror the subsections of the Results section in the main text.

Supplementary Note 5.1 Spatial analysis of individual cells

Supplementary Note 5.1.1 Circularity

Description: Circularity measures how closely a shape resembles a circle.
Definition: Let O be a polygon with area, A, and perimeter, P. Then, the circularity, C, of O is defined as

$$C = \frac{4\pi A}{P^2}. \tag{1}$$

Application: In Fig 2F, circularity is computed for all 'Cell boundary' objects labelled as SMC or PROG.

Supplementary Note 5.1.2 Principal angle

Description: A 2D shape has two principal axes: the principal major axis is the axis of maximum moment of area (maximising bend resistance), and the principal minor axis is the axis of minimum moment of area (minimum bend resistance) [S41].
Definition: The principal angle is the angle between the principal major axis and the line $y = 0$. Let O be a polygon defined by n vertices $\boldsymbol{v}_i = (x_i, y_i) \in \mathbb{R}^2$ $(1 \leq i \leq n)$, ordered anti-clockwise.

The second moments of area, I_x, I_y and I_{xy}, are given by

$$I_x = \frac{1}{12} \sum_{i=1}^{n-1} (x_i y_{i+1} - x_{i+1} y_i) \left(x_i^2 + x_i x_{x_{i+1}} + x_{i+1}^2 \right), \tag{2}$$

$$I_y = \frac{1}{12} \sum_{i=1}^{n-1} (x_i y_{i+1} - x_{i+1} y_i) \left(y_i^2 + y_i y_{x_{i+1}} + y_{i+1}^2 \right), \tag{3}$$

$$I_{xy} = \frac{1}{24} \sum_{i=1}^{n-1} (x_i y_{i+1} - x_{i+1} y_i) (x_i y_{i+1} + 2 x_i y_i + 2 x_{i+1} y_{i+1} + x_{i+1} y_i). \tag{4}$$

They describe how the vertices of a polygon are distributed with respect the x and y-axis. The principal angle, θ_O, of O is defined by

$$\theta_O = \frac{1}{2} \arctan \left(\frac{2 I_{xy}}{I_y - I_x} \right). \tag{5}$$

Application: In Fig 2G, the principal angle is computed for all 'Cell boundary' objects with labels SMC or PROG.

Supplementary Note 5.2 Cell proximity analysis

Supplementary Note 5.2.1 Nearest neighbour metrics

Description: The Average Nearest Neighbour Distance is the mean minimum distance from an object in one population to an object in another. It is closely related to the Average Nearest Neighbour Index (ANNI), which measures the degree of clustering (or dispersion) of a set of points by comparing the average nearest neighbour distance to the expected nearest neighbour distance in a random distribution of the same density of points, here assumed to be complete spatial randomness (CSR) [S8].

Definition: Let Ω be a spatial domain with area α. Let $d_{A,B}$ denote the minimum Euclidean distance between objects A and B, for A, B contained in Ω. Let $\mathcal{O}_A = \{A_1, ..., A_n\}$ and $\mathcal{O}_B = \{B_1, ..., B_m\}$ be two sets with objects A_i $(i = 1, ..., n)$ and B_j $(j = 1, ..., m)$ in Ω. Then the minimum nearest neighbour distance from a specific object A_k to an object in the set $\mathcal{O}_B$ is

$$\mathrm{NN}(A_k; \mathcal{O}_B) = \min_{j=1,\ldots,m} \{d_{A_k,B_j}\}. \tag{6}$$

The average nearest neighbour distance for objects from the set $\mathcal{O}_A$ to those in $\mathcal{O}_B$ is then

$$\mathrm{ANN}(\mathcal{O}_A, \mathcal{O}_B) = \frac{1}{n} \sum_{i=1}^{n} \mathrm{NN}(A_i; \mathcal{O}_B). \tag{7}$$

Assuming CSR, the expected average nearest neighbour distance, $\overline{\mathrm{ANN}}(\mathcal{O}_A; \mathcal{O}_B)$, is given by

$$\overline{\mathrm{ANN}}(\mathcal{O}_A; \mathcal{O}_B) = \frac{1}{2}\sqrt{\frac{\alpha}{m}}. \tag{8}$$

The Average Nearest Neighbour Index, $\mathrm{ANNI}(\mathcal{O}_A; \mathcal{O}_B)$ is expressed as the ratio of the observed to the expected average nearest neighbour distance:

$$\mathrm{ANNI}(\mathcal{O}_A; \mathcal{O}_B) = \frac{\mathrm{ANN}(\mathcal{O}_A; \mathcal{O}_B)}{\overline{\mathrm{ANN}}(\mathcal{O}_A; \mathcal{O}_B)}. \tag{9}$$

The significance of the difference between the observed nearest neighbour distribution and that expected under CSR is typically quantified using the z-score (standard normal variate), z, such that

$$z = \frac{\mathrm{ANN}(\mathcal{O}_A; \mathcal{O}_B) - \overline{\mathrm{ANN}}(\mathcal{O}_A; \mathcal{O}_B)}{\sigma_{\mathcal{O}_A,\mathcal{O}_B}}, \tag{10}$$

where the variance, $\sigma_{\mathcal{O}_A,\mathcal{O}_B}$, is evaluated as

$$\sigma_{\mathcal{O}_A,\mathcal{O}_B} = \frac{c\sqrt{\alpha}}{M}, \tag{11}$$

with $c = 0.26136$ [S8].

Application: The ANNI was used to investigate the spatial relationships between SMC and STR1 cells, and PROG and COL cells in Figs. 3B-E.

Supplementary Note 5.2.2 Quadrat correlation matrix

Description: The quadrat correlation matrix (QCM) describes correlations between counts of two labels of interest (here, Celltype IDs) within a set of 'quadrats', a regular tiling of the domain of interest with non-overlapping areas [S2, S6, S28, S42]. For a given pair of labels, the QCM quantifies how different the observed correlation coefficient across the quadrats is from that expected under a random shuffling of the object labels.

Definition: The QCM is calculated by comparing the observed matrix of cell counts within each quadrat, $\mathbf{O}$, against a null distribution of K null matrices, $\mathbf{N^k}$ for $k \in [1, ..., K]$, whose row and column sums are the same as those of $\mathbf{O}$. The standard effect size (SES / z-score) for Celltypes i and j is given by

$$SES_{ij} = (P_{O_{ij}} - \mu[P_{N^k_{ij}}])/\sigma[P_{N^k_{ij}}], \tag{12}$$

where P_A is the partial correlation matrix associated with matrix A, and μ and σ are the mean and standard deviation over all k. The entry of the QCM corresponding to Celltypes i and j is SES_{ij} if the p-value associated with SES_{ij} score is statistically significant after applying the Benjamini-Hochberg correction for multiple comparisons, and 0 otherwise.

Application: In Fig. 3J, the QCM was computed using a square lattice of edge length 75µm and shuffled over 1000 iterations ($K = 1000$). For simplicity, values shown in Fig. 3J are not filtered for statistical significance (so, strictly speaking, this is the SES matrix, rather than the QCM).

Supplementary Note 5.2.3 Topographical Correlation Map (TCM)

Description: The Topographical Correlation Map (TCM) is a spatial statistic derived from the general class of local indicators of spatial association (LISA) [S43], designed to identify the spatially resolved strength of positive or negative correlation between two cell populations [S2, S9]. Unlike the cross-PCF (see below), which provides a global summary, the TCM assigns a continuous-valued mark to each individual cell and smooths these to produce a spatial map of local associations.

Definition: Let C_A and C_B be two cell populations, and let $\boldsymbol{x}_i$ denote the spatial location of a cell of type C_A. To compute the TCM, we first define a mark $m_{AB}(r, \boldsymbol{x}_i)$ for each such cell, which quantifies its cumulative contribution to the cross-PCF up to radius r:

$$m_{AB}(r, \boldsymbol{x}_i) = \sum_{j=1}^{n} \mathbb{I}(C_B, c_j) \frac{I_{[0,r)}(|\boldsymbol{x}_i - \boldsymbol{x}_j|)}{\alpha_r(\boldsymbol{x}_i)} \Big/ \frac{N_{C_B}}{\alpha}, \tag{13}$$

where α is the total area of the domain, N_{C_B} is the number of cells of type C_B, $\alpha_r(\boldsymbol{x}_i)$ is the area of intersection between an annulus of radius r centred at $\boldsymbol{x}_i$ and the domain, and $\mathbb{I}(C_B, c_j)$ is an indicator function equal to 1 if $c_j \in C_B$, and 0 otherwise. This mark is conceptually similar to the cross-PCF: values $m_{AB} > 1$ indicate local clustering of C_B around $\boldsymbol{x}_i$, while values $m_{AB} < 1$ indicate local exclusion.

To facilitate comparison and aggregation across cells, m_{AB} is normalised to a bounded, symmetric score $\mu_{AB}(r, \boldsymbol{x}_i)$, using a tuning parameter γ:

$$\mu_{AB}(r, \boldsymbol{x}_i) = \begin{cases} 1 & \text{if } m_{AB} \geq \gamma, \\ \frac{m_{AB}-1}{\gamma-1} & \text{if } 1 < m_{AB} < \gamma, \\ \frac{1-\frac{1}{m_{AB}}}{\gamma-1} & \text{if } \frac{1}{\gamma} < m_{AB} < 1, \\ -1 & \text{if } m_{AB} \leq \frac{1}{\gamma}. \end{cases} \tag{14}$$

This ensures that $\mu_{AB} \in [-1, 1]$, where values near 1 and -1 represent the strongest possible clustering and exclusion respectively (bounded by γ), and values near 0 indicate no association. The scaling is symmetric, so $\mu_{AB} = 0.5$ and $\mu_{AB} = -0.5$ represent equal strengths of clustering and exclusion, respectively.

To generate a spatial map over the domain, each $\mu_{AB}(\boldsymbol{x}_i)$ is convolved with a Gaussian kernel of standard deviation σ, centred at $\boldsymbol{x}_i$. The resulting TCM, $\Gamma_{AB}(r, \boldsymbol{x})$, is defined as:

$$\Gamma_{AB}(r, \boldsymbol{x}) = \sum_{i=1}^{N_{C_A}} \frac{\mu_{AB}(r, \boldsymbol{x}_i)}{2\pi\sigma^2} \exp\left(-\frac{1}{2}\left(\frac{|\boldsymbol{x} - \boldsymbol{x}_i|}{\sigma}\right)^2\right). \tag{15}$$

Note that the TCM is not symmetric in its arguments; that is, $\Gamma_{AB}(r, \boldsymbol{x}) \neq \Gamma_{BA}(r, \boldsymbol{x})$, since the kernel locations are determined by the C_A population.
Application: The TCM was applied in the multiscale spatial profiling of immune–fibroblast interactions in CMS4-like colorectal tumours to quantify the spatial heterogeneity of cell–cell associations within regions of interest (ROIs). In this context, we examine selected fibroblast–immune cell type pairs using $r = 20$µm and $\sigma = 25$, to highlight localised clustering or exclusion patterns across the tissue microenvironment.

Supplementary Note 5.3 Analysis of cell-cell relationships across scales

Supplementary Note 5.3.1 Cross pair correlation function

Description: The cross pair correlation function (cross-PCF) is a spatial statistic that relates the observed number of point pairs from populations A and B that are separated by distance r against the expected number under a statistical null distribution.
Definition: Let $\mathbf{x}^A$ and $\mathbf{x}^B$ be the sets of locations of points in populations A and B respectively, and suppose there are N_A and N_B points in each set. Consider the indicator function

$$I_{[p,q)}(r) = \begin{cases} 1 & \text{if } r \in [p, q), \\ 0 & \text{otherwise.} \end{cases} \tag{16}$$

The cross-PCF, $g_{AB}(r)$, is given by

$$g_{AB}(r) = \frac{1}{N_A} \sum_{a=1}^{N_A} \sum_{b=1}^{N_B} \frac{I_{[0,dr)}(|\mathbf{x}_a^A - \mathbf{x}_b^B| - r)}{\alpha_r(\mathbf{x}_i)} / \frac{N_B}{\alpha}, \tag{17}$$

where α is the area of the domain of interest, and $\alpha_r(\mathbf{x})$ is the area of the intersection between the domain and an annulus centred at $\mathbf{x}$ with inner radius r and outer radius dr. This provides a boundary correction term for points close to the domain edge; alternative methods for boundary correction can be found in [S29].
Application: In Fig. 4B we use $dr = 15$µm and calculate $g_{AB}(r)$ at discrete values $r_k \in [0, 5, 10, ..., 250]$, excluding any points within 20µm of the region boundary from the calculation.

Supplementary Note 5.3.2 Vietoris-Rips filtration

Description: A Vietoris–Rips (VR) filtration is a method from persistent homology that can be used to quantify the topological features (e.g. connected components, loops, voids) of point clouds at multiple spatial scales [S35].
Definition: Let X denote a finite set of points in a domain, $\Omega \subset \mathbb{R}^2$, and $d_{i,j}$ represent the distance between points x_i and x_j. For a given scale parameter $\epsilon \geq 0$, the VR complex $R_\epsilon(X)$ is a simplicial complex defined as follows. A finite subset $\sigma = \{x_0, x_1, \ldots, x_k\} \subseteq X$ forms a k-simplex in $R_\epsilon(X)$ if and only if the pairwise distances between all points in σ satisfy $d_{i,j} \leq \epsilon$ for all $0 \leq i, j \leq k$. As the scale parameter ϵ increases, the VR complexes are nested, satisfying $R_{\epsilon_1}(X) \subseteq R_{\epsilon_2}(X)$ for any $0 \leq \epsilon_1 \leq \epsilon_2$. This nested sequence forms a filtration of simplicial complexes, denoted by $\{R_\epsilon(X)\}_{\epsilon \geq 0}$.

To quantify these topological features, the homology groups $H_k(R_\epsilon(X))$ of the VR complexes are computed for each dimension k as follows. Each k-simplex in $R_\epsilon(X)$ there exists a boundary operator ∂_k that maps it to a formal sum of its $(k-1)$-dimensional faces. The boundary operators satisfy the composition property $\partial_k \circ \partial_{k+1} = 0$, which ensures that the image of ∂_{k+1} is contained within the kernel of ∂_k. The k-th homology group is then defined as the quotient

$$H_k(R_\epsilon(X)) = \frac{\ker(\partial_k)}{\operatorname{im}(\partial_{k+1})}. \tag{18}$$

It captures the k-dimensional topological features, such as connected components ($k = 0$), loops ($k = 1$), and higher-dimensional voids. Persistent homology extends this computation across the filtration $\{R_\epsilon(X)\}_{\epsilon \geq 0}$. As ϵ increases, the boundary maps and the resulting homology groups change, leading to the birth and death of topological features.
Application: VR filtrations were used to identify crypt-like structures in Fig. 4F in the PROG population by restricting our attention to 0 and 1-dimensional features ($k \leq 1$) and defining $d_{i,j}$ as the minimum Euclidean distance between point objects i and j.

Supplementary Note 5.4 Spatial relationships at tissue scales

Supplementary Note 5.4.1 Neighbourhood clustering

Description: Microenvironment clustering seeks to group objects with similar compositions of labels located within a local neighbourhood of an object. These methods are inspired by those deployed in [S3].
Definition: Let $\mathcal{G}(V, E)$ be a network with nodes $v_i \in V$ and edges $e_{ij} \in E$ encode spatial relationships between nodes. Each node has a categorical label $c_i \in \{C_1, \ldots, C_m\}$ (i.e., its cell type). Let the microenvironment, $\mathcal{N}_i$, be the set of nodes about node i defined by a k-hop neighbourhood (see Table 1). For each neighbourhood, $\mathcal{N}_i$, the composition of each label category is computed to generate an observation matrix $\boldsymbol{O} \in \mathbb{N}^{|V| \times m}$ where

$$[\boldsymbol{O}]_{i,s} = \frac{1}{|\mathcal{N}_i|} \sum_{j \in \mathcal{N}_i} \delta(c_j, C_s) \tag{19}$$

and

$$\delta(c_i, C_s) = \begin{cases} 1 \text{ if } c_i = C_s, \\ 0 \text{ otherwise.} \end{cases} \tag{20}$$

Row-wise clustering is performed on $\boldsymbol{O}$ to identify similar microenvironment compositions, which partition the nodes $v_i \in V$ into disjoint sets S_t.

Cluster representatives (i.e., the mean or medoid composition vector of the cluster) are extracted from each set S_t, denoted by M_{i,S_t}.

Define the average composition of label C_i of the sample, M_i, by

$$M_i = \frac{1}{|V|} \sum_{j=1}^{|V|} \delta(c_j, C_i). \quad (21)$$

An microenvironment enrichment score, E_{i,S_t}, is then calculated by compare the representatives to the average composition,M_i, define as a log-fold change

$$E_{i,S_t} = \log_2 \left(\frac{M_{i,S_t}}{M_i} \right). \quad (22)$$

Application: In Fig. 5D, $\mathcal{G}(V, E)$ is a Delaunay network (see Table 1) on cell centres with edge distance threshold of 30µm. A 3-hop neighbourhood was used to generate microenvironment sets about each node. Clustering was performed using k-means with $k = 5$.

Supplementary Note 5.4.2 Adjacency permutation test

Description: The adjacency permutation test quantifies the significance of labelled nodes being adjacent in a network. This non-parametric test involves repeatedly shuffling node labels to generate a null distribution of a test statistic under the hypothesis of no spatial association [S7].

Definition: Let $\mathcal{G}(V, E)$ be a network with nodes $v_i \in V$ and edges $e_{ij} \in E$ where each node has a categorical label $c \in \{C_1, \ldots, C_m\}$. For every pair of categorical labels C_s and C_t, the number of observed edges, n_{C_s,C_t} , is calculated between nodes with these labels such that

$$n_{C_s,C_t} = \sum_{e_{ij} \in E} \delta(c_i, C_s)\, \delta(c_j, C_t), \quad (23)$$

where $\delta(c_i, C_s)$ is defined in equation (20). An observation matrix, $\boldsymbol{N} \in \mathbb{N}_0^{m \times m}$, is constructed using each categorical pair count, $[\boldsymbol{N}]_{s,t} = n_{C_s,C_t}$, for $1 \leq s, t \leq m$.

The node labels are then shuffled and the adjacent pair count procedure is repeated for K iterations, generating a null distribution of count matrices $\tilde{\boldsymbol{N}}_k$ for $1 \leq k \leq K$. The adjacency significance score for each pair of categorical labels, $S_{s,t}$, is calculated as z-score of the observed to the null,

$$S_{s,t} = \frac{[\boldsymbol{N}]_{s,t} - \mu\left([\tilde{\boldsymbol{N}}_k]_{s,t}\right)}{\sigma\left([\tilde{\boldsymbol{N}}_k]_{s,t}\right)} \quad (24)$$

are the mean and standard deviation over all k.

Application: The Adjacency Permutation Test was used to determine the adjacency significance of the ME regions that are in close proximity in Fig. 5G. After converting spatially contiguous regions of points with identical ME labels into shapes (ME regions, Fig. 5F), a proximity network of these shapes was generated where edges between ME regions were drawn if they less than 5µm apart. This proximity network equipped with ME ID labels was then subjected to a Adjacency Permutation Test with 1000 random samples ($K = 1000$).

Supplementary Note 5.4.3 Local Getis-Ord

Description: The local Getis-Ord statistic G_i^* is measures spatial autocorrelation by relating spatial weights (e.g., distances between objects) with label values [S32].
Definition: Let $\boldsymbol{x}_i \in \mathbb{R}^2$ represent the spatial position of the i-th object, $1 \leq i \leq n$, and associated with a continuous label $l_i \in \mathbb{R}$. If $w_{i,j} \in \mathbb{R}$ defines the spatial relationship between $\boldsymbol{x}_i$ and $\boldsymbol{x}_j$, then the local Getis-Ord statistic, G_i^*, for object i is given by

$$G_i^* = \frac{\sum_{j=1}^n w_{ij} l_j}{\sum_{i=1}^n \sum_{j=1}^n w_{ij} l_j}. \tag{25}$$

G_i^* is the weighted average of label values adjacent to object i. The value of G_i^* relative to the mean identifies whether an object belongs to a *hotspot* (+ve) or *coldspot* (-ve) for that label is typically reported as a z-score, z,

$$Z = \frac{G_i^* - \mu(G_i^*)}{\sigma(G_i^*)} \tag{26}$$

where μ and σ are the mean and standard deviation over all i, respectively.
Application: In Fig. 5I, the objects are hexagons, and we assign a weight function for the shapes i and j of the form

$$w_{ij} = \begin{cases} 1 \text{ if } d_{i,j} = 0, \\ 0 \text{ otherwise}, \end{cases} \tag{27}$$

where $d_{i,j}$ is the minimum distance between objects i and j. The labels l_i are the number of cells of PROG cells located within hexagon i.

Supplementary Note 5.4.4 Gaussian kernel density estimation

Description: Kernel density estimation (KDE) is a non-parametric method for estimating a probability density function from observations.
Definition: A Gaussian KDE uses Gaussian kernels centred on each observation, $\boldsymbol{x}_i$, to generate a distribution over a domain $\Omega \subset \mathbb{R}^2$. The resultant probability density function, $P(\boldsymbol{x})$, is determined by

$$P(\mathbf{x}) = \frac{1}{n\sigma\sqrt{2\pi}} \sum_{i=1}^{n} \exp\left(\frac{-||\boldsymbol{x} - \boldsymbol{x}_i||^2}{2\sigma^2}\right), \tag{28}$$

where n is the number of observations and σ is the bandwidth of the kernel. Increasing σ generates smoother representations of the observed point data, $\boldsymbol{x}_i$.
Application: In Fig. 5J, we independently apply Gaussian kernels, centred on SMC, PROG, and STR1 cells, with $\sigma = 0.05$.

Supplementary Note 5.4.5 Wasserstein distance

Description: The 2-Wasserstein distance (referred to here as the Wasserstein distance), describes the minimum distance required to transform one distribution onto another (see [S44] for further details).
Definition: Consider two discrete probability distributions $\boldsymbol{\mu} = (\mu_1, ..., \mu_n)$ and $\boldsymbol{\nu} = (\nu_1, ..., \nu_m)$ with spatial locations $\boldsymbol{x} = (\boldsymbol{x}_1, \ldots, \boldsymbol{x}_n)$ and $\boldsymbol{y} = (\boldsymbol{y}_1, \ldots, \boldsymbol{y}_m)$ within a domain $\Omega \subset \mathbb{R}^2$, respectively.

Then the 2-Wasserstein distance is defined as

$$W_2(\boldsymbol{\mu}, \boldsymbol{\nu}) = \min_{\boldsymbol{\pi} \in \Pi(\boldsymbol{\mu}, \boldsymbol{\nu})} \sum_{i,j} ||\boldsymbol{x}_i - \boldsymbol{y}_j||_2 \boldsymbol{\pi}_{i,j} \tag{29}$$

where $||\cdot||_2$ is the l_2-norm and

$$\Pi(\boldsymbol{\mu}, \boldsymbol{\nu}) = \{\boldsymbol{\pi}' \in \mathbb{R}_{\geq 0}^{n \times m} : \boldsymbol{\pi}' \mathbf{1}_m = \boldsymbol{\mu}, \boldsymbol{\pi}'^T \mathbf{1}_n = \boldsymbol{\nu}\} \tag{30}$$

is the set of all possible transport plans from $\boldsymbol{\mu}$ to $\boldsymbol{\nu}$. The mapping $\boldsymbol{\pi}$ provides the optimal transport plan that transforms $\boldsymbol{\mu}$ onto $\boldsymbol{\nu}$ with the minimum work.

Solving for $\boldsymbol{\pi}$ is computationally inefficient for distributions in more than one dimension and therefore we employ the sliced method to determine $\boldsymbol{\pi}$ [S45]. We project $\boldsymbol{\mu}$ and $\boldsymbol{\nu}$ onto a one-dimensional line drawn through the barycentre of the distributions at an angle $\theta \sim U[0, \pi)$ and then determine the 1-dimensional Wasserstein determined,

$$W_2(\mu^\theta, \nu^\theta) = \min_{\pi^\theta \in \Pi(\mu^\theta, \nu^\theta)} \sum_{i,j} ||x_i^\theta - y_j^\theta||_2 \pi_{i,j}^\theta. \tag{31}$$

The Wasserstein distance of the full-dimension dataset is then approximated by the root average of these projected distances such that

$$W_2(\boldsymbol{\mu}, \boldsymbol{\nu}) \approx \sqrt{\frac{1}{t} \sum_{k=1}^{t} W_2(\mu^{\theta_k}, \nu^{\theta_k})}. \tag{32}$$

Critically, equation (32) holds in equality in the limit $t \to \infty$ [S44].
Application: In Fig. 5K, μ and ν are weighted uniformly such that $\mu_i = \frac{1}{n}$ and $\nu_j = \frac{1}{m}$ for all $1 \leq i \leq n$ and $1 \leq j \leq m$. In addition, $t = 500$ was selected as the number of sliced projections for sufficient coverage of the spatial data.

Supplementary Note 5.4.6 KL-Divergence

Description: The Kullback-Leibler (KL) divergence is a method for comparing the statistical distance between two probability distributions by measuring the entropy of a distribution relative to the other [S17].
Definition: Let P and Q be two probability distributions defined over the sample space $\mathcal{X}$. The KL divergence is defined as

$$\mathrm{D}_{KL}(P||Q) = \sum_{\mathbf{x} \in \mathcal{X}} P(\mathbf{x}) \log\left(\frac{P(\mathbf{x})}{Q(\mathbf{x})}\right). \tag{33}$$

Thus, $D_{KL}(P||Q)$ measures the expected logarithmic difference between the probabilities P and Q, with the expectation taken under the distribution defined by P.
Application: In Fig. 5K, P and Q are the Gaussian KDEs associated with of the point clouds of the corresponding SMC, PROG, and STR1 cells.

Supplementary Note 6 Supplementary information for multiscale spatial profiling of fibroblast-immune interactions in CRC

Supplementary Note 6.1 Extended mouse information

All procedures were carried out in accordance with Home Office UK regulations and the Animals (Scientific Procedures) Act 1986. All mice were housed in individually ventilated cages at the animal unit either at the Functional Genetics Facility (Wellcome Centre for Human Genetics, University of Oxford). All mice were housed in a specific-pathogen-free (SPF) facility, with unrestricted access to food and water, and were not involved in any previous procedures. All strains used in this study were maintained on a C57BL/6J background for ≥6 generations. Mice were sacrificed when they reached humane-end points (exhibited anaemia, hunching and inactivity).

Supplementary Note 6.2 Cell type annotation

Spatial transcriptomic data from four AKPT murine colorectal cancer (CRC) samples were provided as HDF5 files containing regions of interest and processed using Seurat (v5.2.0) in R (v4.3.3). Each sample was converted into a Seurat object, merged, collapsed into a single layer using `JoinLayers()`, and normalized using `LogNormalize` with a scale factor of 500 (Supplementary Fig. 11A). Dimensionality reduction and clustering were performed using PCA and UMAP (dimensions 1–20), followed by initial clustering at a resolution of 0.1 (Supplementary Fig. 11B). Clusters expressing epithelial markers (*Epcam*, *Cdh1*) were identified and removed (Supplementary Fig. 11C), after which the remaining 'non-epithelial' cells were re-clustered at a resolution of 0.6 to identify stromal and immune populations (Supplementary Fig. 11D).

For each of the resulting 22 clusters, aggregate scaled expression of fibroblast (*Pdgfra*) and immune-associated genes (*Ptprc*, *Cd68*, *Ms4a7*, *Trem2*, *Spp1*, *Clec9a*, *Cxcl2*, *Foxp3*, *Cd3e*, *Cd8a*, and *Mzb1*) was calculated (Supplementary Figs. 11E-F). Clusters were annotated as fibroblast if *Pdgfra* expression was > 0, or immune if *Ptprc* expression was > 0. Clusters 3 and 4 expressed both markers but were assigned as fibroblast based on higher *Pdgfra* levels (cluster 3: *Pdgfra* = 2.30, *Ptprc* = 0.25; cluster 4: *Pdgfra* = 2.45, *Ptprc* = 0.03). This enabled a low-resolution classification of fibroblast, immune, and unlabelled populations.

Immune clusters were further subtyped by visualising scaled aggregate expression of immune markers using `ComplexHeatmap` (v2.18.0) (Supplementary Fig. 11G). Clusters enriched for distinct immune gene signatures were annotated as specific immune cell types, resulting in the identification of 12 immune populations, with the remaining clusters left unlabelled (Supplementary Fig. 11H).

Supplementary Note 6.3 Extended analysis methods

Dimension reduction

Dimensionality reduction of transcriptional counts and multiscale spatial feature vectors was performed using UMAP for visualisation. For each two-dimensional embedding, we set *n_neighbors*=100 for transcriptional data and *n_neighbors*=150 for spatial feature data, using the *Manhattan* distance metric in both datasets.

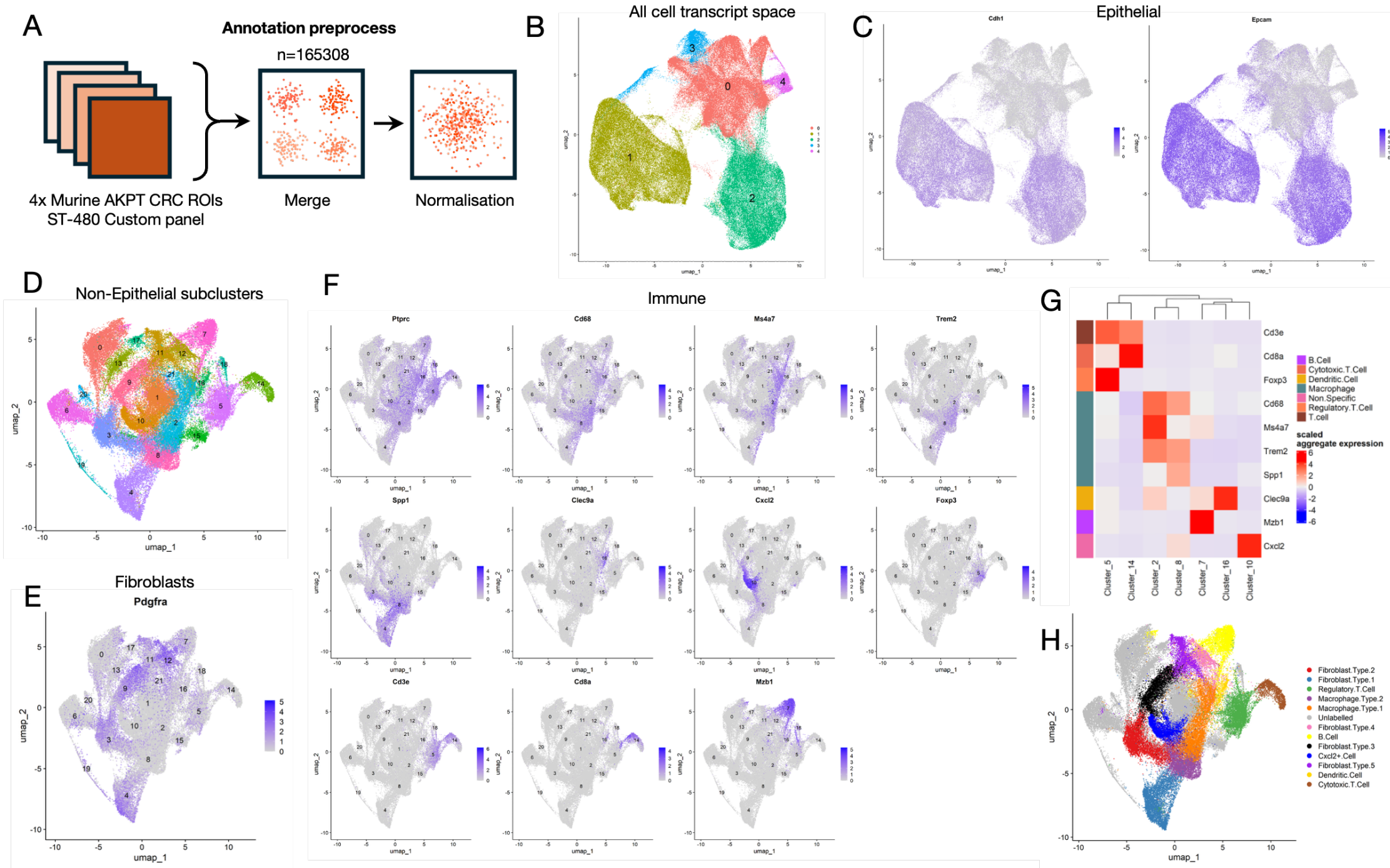

Supplementary Figure 11: **Annotation of murine AKPT colorectal tumours.**
A–H: Cell type annotation of four Xenium (480-gene custom panel) AKPT colorectal tumour samples. A: Schematic overview of the preprocessing workflow for cell type annotation. B: Two-dimensional UMAP projection of the 480-gene transcript space across all samples, coloured by coarse clustering. C: Expression of epithelial markers *Cdh1* and *Epcam* mapped onto the transcript UMAP from A. D: UMAP projection of transcript expression from non-epithelial cells (clusters 0, 3, and 4 from B), coloured by fine-grained clustering (clusters 0–21). E: Expression of the fibroblast marker *Pdgfra* on the non-epithelial UMAP projection from D. F: Expression of immune cell markers (*Ptprc, Cd68, Ms4a7, Trem2, Spp1, Clec9a, Cxcl2, Foxp3, Cd3e, Cd8a*, and *Mzb1*) on the non-epithelial UMAP projection from D. G: Heatmap showing hierarchical clustering of immune marker expression with supervised immune cell label annotations. H: Final cell type annotations for fibroblasts and immune cells overlaid on the non-epithelial transcript UMAP. Grey points indicate unclassified cells.

Trajectory inference

To infer a sequential organisation of fibroblast types, we constructed a weighted minimum spanning tree (WMST) connecting the medoids of transcriptionally defined fibroblast clusters [S46]. Pairwise distances between medoids were calculated using the 1-norm (Manhattan distance) of their transcript count vectors. The WMST was computed using the `minimum_spanning_tree` function from `networkx` (v3.4.2), and projected into the UMAP space to visualise inferred cluster trajectories.

Generating MuSpAn domains

MuSpAn domains for each AKPT colorectal cancer sample were extracted directly from Xenium Explorer (v3) using the `xenium_to_domain` helper function from the `io` module of MuSpAn. This function automatically maps cell boundary geometries to their corresponding annotations. A

pathology-derived region, annotated in Xenium Explorer (v3), was incorporated into the domain using the `add_shapes` function. Sample boundaries were estimated using an alpha shape ($\alpha = 100$) via the `estimate_boundary` function. Cell centroids were computed using the `convert_objects` function, allowing each cell to be represented either as a shape or a point for downstream spatial analysis.

Tumour annotation analysis

To assess whether selected fibroblasts were located within tumour annotations, we used the `is_A_contained_by_B` function from the `query` module of MuSpAn. Fibroblasts within tumour regions were assigned to an *in tumour* collection, and the proportion of each fibroblast type within this collection was calculated.

Cell-cell contact analysis

To analyse cell–cell contact, we first constructed a proximity network for each sample, representing physical interactions between adjacent cell boundaries. Networks were generated using the *proximity* method within the `generate_network` function of the `networks` module of MuSpAn, with contact defined as a distance of $\leq 1.5\,\mu$m between cell boundaries. Each resulting network was stored within the corresponding sample domain for downstream analysis.

Contact adjacency correlations were assessed using the `adjacency_permutation_test` function in the `networks` module, with statistical significance threshold $\alpha = 0.05$ and 500 randomised permutations on the contact networks previously constructed. This test was applied independently to each sample.

Topographical Correlation Maps (TCMs) were generated to visualise patterns of contact between immune and fibroblast populations within each sample. TCMs were computed using the `topographical_correlation_map` function from the `spatial_statistics` module of MuSpAn, with interaction radius $r = 20\,\mu$m, Gaussian kernel variance $\sigma = 25$, and a maximum correlation threshold of $\alpha = 3$. Further details on the `adjacency_permutation_test` and `topographical_correlation_map` are provided in Supplementary Note 5.

Neighbourhood analysis

To investigate immune neighbourhood composition surrounding selected fibroblasts, we use the `cluster_neighbourhoods` function from the `networks` module in MuSpAn. Neighbourhoods were defined as all nodes reachable within three edges (3-hop neighbourhoods; $k\_hop = 3$) from each fibroblast of interest, using the previously constructed cell boundary contact network.

For each fibroblast, the immune cell composition within its 3-hop neighbourhood was computed and square-root transformed across all samples. These transformed profiles were then clustered using k-means clustering ($cluster\_method =$ `kmeans`) with $k = 4$ ($n\_clusters = 4$). Cluster enrichment was quantified by computing the z-score of the representative centroid of each cluster ($neighbourhood\_enrichment\_as =$ `zscore`). Mathematical details for the `cluster_neighbourhoods` function are provided in Supplementary Note 5.

Spatial correlation at multiple scales

To assess spatial associations between selected fibroblast and immune cell populations across multiple length scales, we used the cross–Pair Correlation Function (cross-PCF). The cross-PCF was computed using the `cross_pair_correlation_function` from the `spatial_statistics` module of

MuSpAn. For all analyses, correlations were calculated up to a maximum radius of 500 μm (max_R = 500 μm), with an annulus width of 30 μm ($annulus\_width$ = 30 μm).

As a summary statistic, we computed the cumulative cross-PCF ratio, $C_{AB}(R)$, which captures the integrated spatial association between two populations up to radius R, defined as

$$C_{AB}(R) = \frac{1}{R}\int_0^R g_{AB}(r)\,dr, \tag{34}$$

where $g_{AB}(r)$ is the cross-PCF between populations A and B, and $R = 150\,\mu$m. The cumulative ratio is normalised against the expected value under a spatial null model, preserving interpretation of the cross-PCF. Further mathematical details of the cross-PCF computation are provided in Supplementary Note 5.

Multiscale spatial feature vectors

To enable integrated analysis of the fibroblast spatial context, we constructed multiscale feature vectors for each fibroblast by combining outputs from the aforementioned analyses. For each fibroblast, local contact-based neighbourhood features (1-hop and 3-hop immune subtype counts), TCM value with each immune population at it's location, and spatial association statistics derived from cross–pair correlation functions (cross-PCFs). Cross-PCF features included the cumulative spatial correlation up to 150 μm, as well as values sampled at radii of 0, 100, 200, 300, and 400 μm, capturing spatial interactions across multiple scales. The resultant pairwise computations between the fibroblast and immune subtypes produced a 87-dimensional vector for each fibroblast.

All features were computed independently for each fibroblast across all samples and combined into a single unified dataframe. To ensure comparability between features, each dimension was z-score normalised across all fibroblasts. For each fibroblast type, we computed the median of its multiscale feature vectors to obtain a representative profile. A WMST was then constructed using the 1-norm distance matrix between these fibroblast type medians. To visualise the inferred progression of fibroblast–immune interactions, both the fibroblast-level feature vectors and the WMST edges were projected into a shared two-dimensional space using the same UMAP embedding.

Supplementary Note 6.4 Assessing the effect of tumour compartmentalisation on fibroblast–immune spatial associations

To determine whether the fibroblast–immune spatial associations identified in AKPT tumours could be explained by tumour compartmentalisation, we performed an additional compartment-aware analysis using pathological annotations. As a representative example for comparing spatial association within and outside the tumour region, we focused on the CAF3–Cxcl2$^+$ Cell pair as both populations are present in both annotated regions (Supplementary Fig. 12A).

For a representative AKPT sample, we defined three analysis regions: (i) the whole tissue section (*All*); (ii) the area within the pathological tumour annotation (*In tumour*); and (iii) the area outside the tumour annotation (*Out of tumour*). Within each region, we repeated the Adjacency Permutation Test (APT; Fig. 6F-G) and cross-pair correlation function (xPCF; Fig. 6N-O) analyses for the CAF3–Cxcl2+ Cell pair (see also Supplementary Note 3).

Compartment-specific analysis was conducted using the `include_boundaries` and `exclude_boundaries` keyword arguments available for all MuSpAn analysis metrics, which restrict spatial metrics to computation within or outside the specified shapes while ensuring appropriate

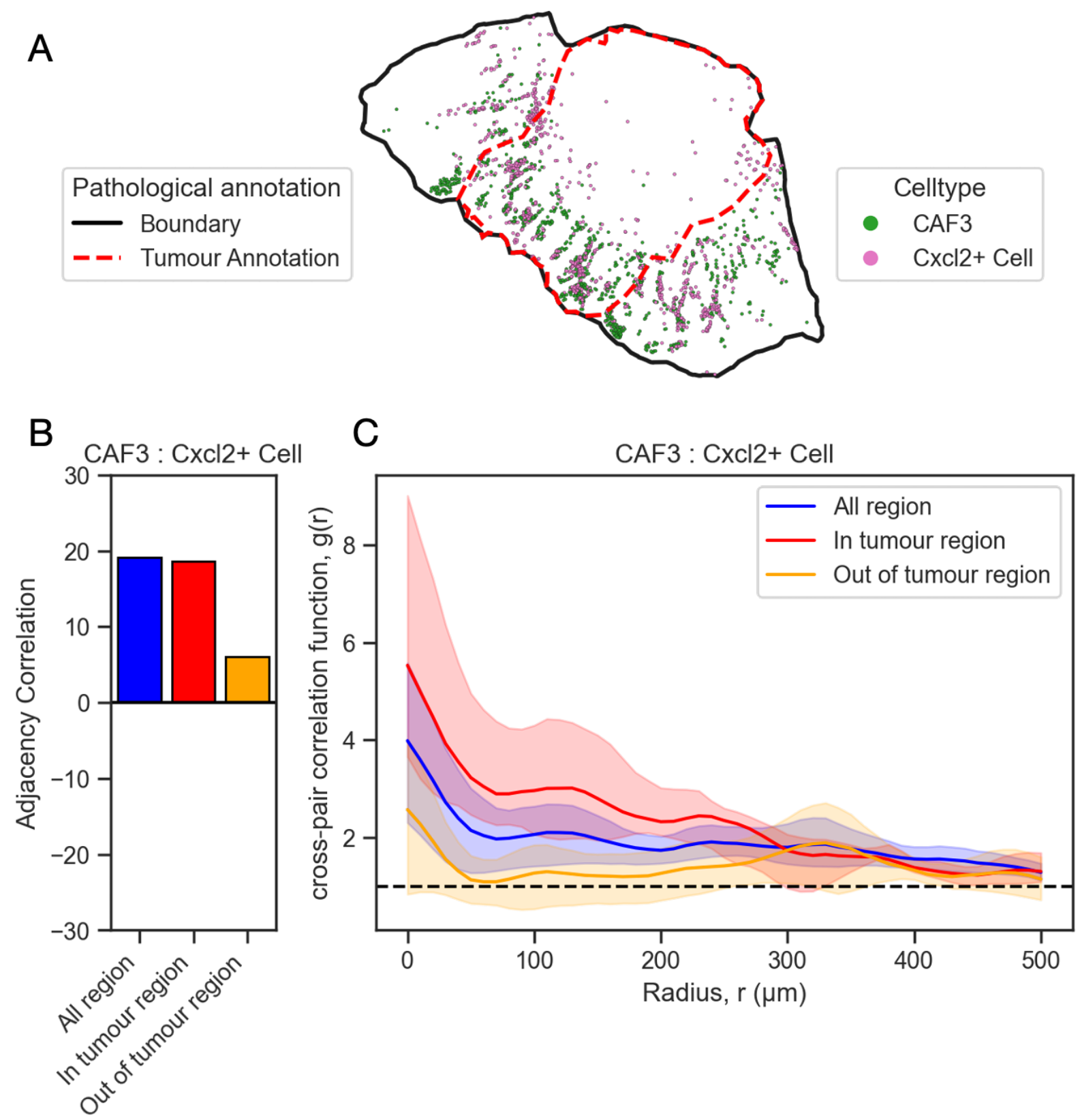

Supplementary Figure 12: **CAF3–Cxcl2$^+$ Cell spatial association is preserved across tumour compartments**

A: Representative region of interest from an AKPT tumour sample (Fig. 6), with pathological annotations defining tumour and non-tumour compartments. CAF3 and Cxcl2$^+$ Cells are overlaid (other cell populations not shown here).

B: Adjacency permutation test scores for CAF3–Cxcl2+ Cell pairs, computed across the whole region of interest, within the tumour compartment, and outside the tumour compartment. Scores are shown as z-scores for each analysis region.

C: Cross-pair correlation functions for CAF3–Cxcl2+ Cell pairs computed across the whole region of interest, within the tumour compartment, and outside the tumour compartment. Curves show the cross-pair correlation function as a function of radius, reported in μm.

boundary corrections are applied. Since MuSpAn automatically accounts for complex tissue and annotation boundaries during spatial quantification, the resulting measurements are comparable across the analysis regions. All parameters for the APT and xPCF analyses were held identical to those used in Fig. 6 (see Supplementary Note 6.3).

Comparing the results across the three regions showed that the spatial association between CAF3 and Cxcl2+ Cells described in Fig. 6 was preserved after controlling for tumour compartmentalisation. The APT analysis indicated significant enrichment in cell–cell contact between CAF3 and Cxcl2+ Cells when compared with randomly shuffled labels when the analysis was conducted either across the whole sample, within the tumour annotation, or outside the tumour annotation (Supplementary Fig. 12B). Similarly, the regional xPCF analysis showed consistent positive correlation at short spatial scales ($\lesssim 50\mu m$), irrespective of whether the analysis was performed across the whole sample or restricted to the tumour or non-tumour compartments (Supplementary Fig. 12C).

Together, these results indicate that the CAF3–Cxcl2+ Cell spatial association observed in AKPT tumours is not solely attributable to pathological tumour compartmentalisation. Instead, the persistence of both local contact enrichment and short-range co-localisation within separate tumour and non-tumour regions supports the interpretation that this fibroblast–immune relationship reflects a local spatial interaction that remains detectable after explicitly controlling for tumour compartment effects.

Supplementary Note 6.5 Fibroblast spatial–transcriptional alignment is independent of tumour compartment effects

The spatial microenvironment of cells differs substantially between pathologically annotated tumour and adjacent non-tumour regions, with distinct cellular compositions and interaction structures reported across these compartments [S6]. As several components of the spatial feature representation (including neighbourhood composition and higher-order interaction summaries) are expected to vary with such compartmental differences, we assessed whether the observed alignment between fibroblast spatial and transcriptional trajectories could be attributed to tumour localisation.

We compared trajectory structure and alignment in two settings: (i) all fibroblasts, and (ii) fibroblasts restricted to annotated tumour regions. As shown previously (Fig. 6E), restricting to tumour regions predominantly excludes CAF4 cells, consistent with their enrichment outside tumour annotations; this shift in composition is reflected in Supplementary Figs. 13A–B.

For each subset (i) and (ii), we recomputed diffusion pseudotime trajectories in both transcriptional space (480-dimensional) and spatial interaction feature space (87-dimensional) using diffusion-based methods [S47]. In addition, 2D UMAP embeddings were recomputed for the tumour-restricted subset (Supplementary Figs. 13C–D) to assess whether the global structure of the manifold was preserved.

Despite the substantial change in spatial context and fibroblast composition, the alignment between transcriptional and spatial trajectories was maintained. Specifically, the Spearman correlation between transcriptional and spatial pseudotime was $\rho = 0.885$ when considering all fibroblasts and $\rho = 0.882$ when restricted to tumour-resident fibroblasts (Supplementary Figs. 13C–D). This indicates that the concordance between transcriptional progression and spatial interaction structure is largely preserved even after accounting for tumour compartmentalisation.

Consistent with this, the ordering of fibroblast states along the trajectory remained stable. In the full dataset, fibroblasts progress from CAF1 through CAF4, whereas in the tumour-restricted analysis the ordering spans CAF1 to CAF3, reflecting the near-complete absence of CAF4 cells in tumour regions rather than a disruption of the underlying trajectory structure (Supplementary Figs. 13C–D).

To further assess whether the observed alignment could arise by chance, we performed a permutation-based significance test. For each subset independently, transcriptional pseudotime

values were randomly permuted across fibroblasts, and the Spearman correlation with spatial pseudotime was recomputed (1,000 permutations), generating a null distribution of alignment statistics. These null distributions were approximately normal (Shapiro–Wilk $p > 0.8$ in both cases). The observed correlations were highly extreme relative to their respective nulls, with z-scores $z_{\text{all}} = 113.13$ and $z_{\text{tumour}} = 101.15$ (both $p < 0.001$, Bonferroni-corrected), demonstrating that the alignment between transcriptional and spatial trajectories is not explained by random association.

Together, these analyses show that the correspondence between fibroblast transcriptional states and spatial interaction features is robust to tumour compartment restriction and is not driven solely by differences between tumour and non-tumour regions.

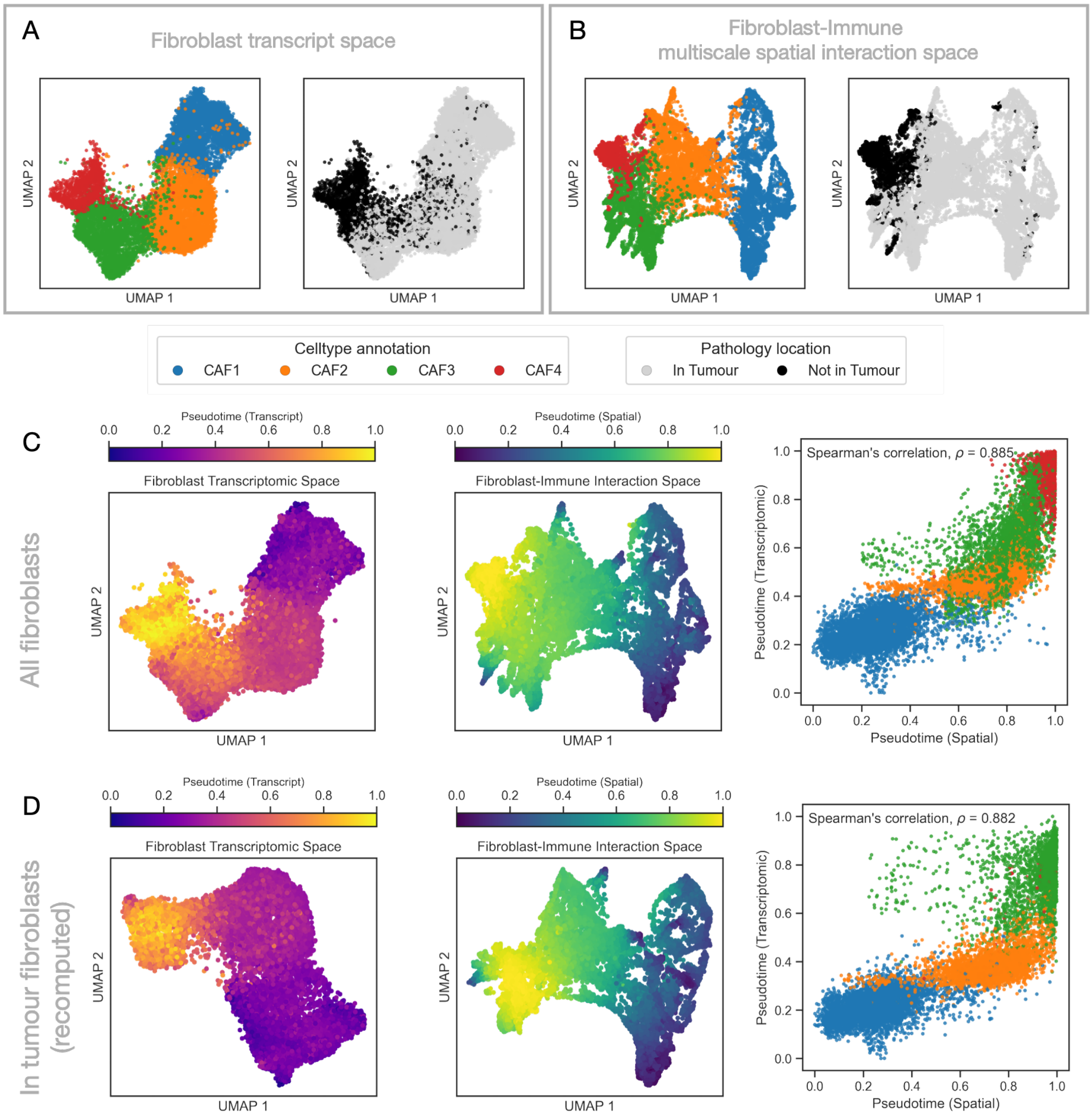

Supplementary Figure 13: **Extended trajectory analysis in fibroblast transcriptional and spatial interaction spaces.**

A–B: UMAP projections of fibroblast transcriptional profiles (A) and multiscale fibroblast–immune interaction features (B), coloured by fibroblast cell type and pathological region (tumour vs non-tumour) (n = 16,512).

C: UMAP projections of all fibroblasts in transcriptional space (left) and spatial interaction space (middle), coloured by diffusion pseudotime inferred in each respective space. Right: comparison of transcriptional and spatial pseudotime values, coloured by fibroblast cell type (n = 16,512).

D: UMAP projections of tumour-resident fibroblasts in transcriptional space (left) and spatial interaction space (middle), coloured by diffusion pseudotime inferred in each respective space. Right: comparison of transcriptional and spatial pseudotime values, coloured by fibroblast cell type (n = 13,397).

Supplementary Note 7 Computational runtime considerations

MuSpAn is designed to operate efficiently on standard personal computing hardware, without requiring access to high-performance computing (HPC) resources. However, computational runtime depends on both the analytical method employed and structural properties of the input data such as the number of objects.

In particular, methods from spatial statistics can incur additional computational cost when applied to geometrically complex domains, due to the need for edge correction and boundary handling (where runtimes of algorithms often scale with both the number of points and the number of edges in a bounding polygon). Similarly, topological approaches such as persistent homology are known to exhibit poor scaling with increasing numbers of objects, reflecting the combinatorial growth of simplicial complexes. In contrast, network-based methods do not require boundary corrections and typically scale with the number of nodes and edges. While this can be efficient for sparse graphs, runtimes may increase substantially for dense networks where the number of edges grows rapidly.

In this supplementary note, we provide a practical overview of computational performance. We evaluated the runtime of a representative set of metrics drawn from spatial statistics, network science, and ecology, applying each statistic to simulated domains containing between 1,000 and 100,000 objects (Supplementary Fig. 14). For moderately sized datasets (approximately 10,000 objects), all evaluated metrics completed within 5 seconds on a standard laptop (MacBook Pro, 2023 model).

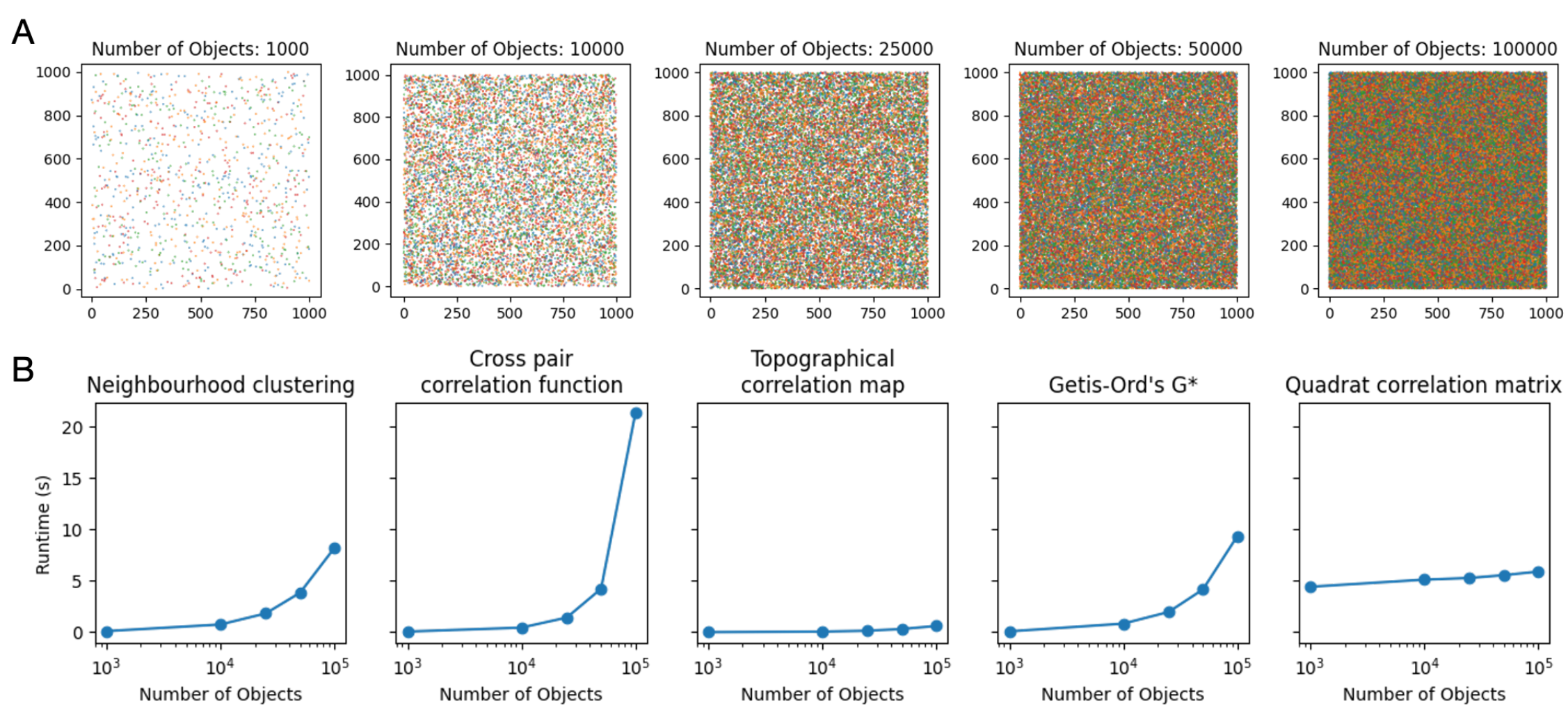

Supplementary Figure 14: **Example computational runtimes.**
A: Simulated spatial domains comprising 1,000–100,000 points generated from a Poisson point process. Each point is randomly assigned one of four categorical labels (A–D), indicated by colour.
B: Computational runtime of selected spatial metrics as a function of the number of objects. All computations were performed on a MacBook Pro (2023; Apple M2 Pro; 16 GB RAM).

In addition, we report the total runtime for all analyses presented in the main text in Supplementary Table 3, providing a comprehensive view of end-to-end computational requirements. Reported times are the cumulative times required to run all analyses to reproduce each figure.

**Analysis**	**Number of Point Objects**	**Number of Shape Objects**	**Total Number of Objects**	**Total Runtime (sec)**
Single cell<br>Fig. 2	70470	3709	74179	7.64
Local proximity<br>Fig. 3	3283	3361	6644	7.11
Multiscale<br>Fig. 4	13689	1	13690	25.67
Tissue scale<br>Fig. 5	126146	690	126836	205.46
Fib-Imm analysis<br>Fig. 6	125306	125310	250616	331.66

Supplementary Table 3: A summary of runtimes for spatial analysis presented in this study. All computations were performed on a MacBook Pro (2023; Apple M2 Pro; 16 GB RAM).

Supplementary Information References

[S1] Joshua A. Bull et al. "Combining multiple spatial statistics enhances the description of immune cell localisation within tumours". en. In: *Scientific Reports* 10.1 (Oct. 2020). Number: 1, p. 18624. ISSN: 2045-2322. DOI: 10.1038/s41598-020-75180-9. URL: https://www.nature.com/articles/s41598-020-75180-9 (visited on 11/27/2023).

[S2] Praveen Weeratunga et al. "Single cell spatial analysis reveals inflammatory foci of immature neutrophil and CD8 T cells in COVID-19 lungs". en. In: *Nature Communications* 14.1 (Nov. 2023). Number: 1, p. 7216. ISSN: 2041-1723. DOI: 10.1038/s41467-023-42421-0. URL: https://www.nature.com/articles/s41467-023-42421-0 (visited on 11/27/2023).

[S3] Christian M. Schürch et al. "Coordinated Cellular Neighborhoods Orchestrate Antitumoral Immunity at the Colorectal Cancer Invasive Front". English. In: *Cell* 182.5 (Sept. 2020), 1341–1359.e19. ISSN: 0092-8674, 1097-4172. DOI: 10.1016/j.cell.2020.07.005. URL: https://www.cell.com/cell/abstract/S0092-8674(20)30870-9 (visited on 11/27/2023).

[S4] Oliver Vipond et al. "Multiparameter persistent homology landscapes identify immune cell spatial patterns in tumors". In: *Proceedings of the National Academy of Sciences* 118.41 (Oct. 2021), e2102166118. DOI: 10.1073/pnas.2102166118. URL: https://www.pnas.org/doi/abs/10.1073/pnas.2102166118 (visited on 11/27/2023).

[S5] Praveen Weeratunga et al. *Temporo-spatial cellular atlas of the regenerating alveolar niche in idiopathic pulmonary fibrosis*. en. Pages: 2024.04.10.24305440. Apr. 2024. DOI: 10.1101/2024.04.10.24305440. URL: https://www.medrxiv.org/content/10.1101/2024.04.10.24305440v1 (visited on 04/29/2024).

[S6] Joshua A. Bull et al. *Integrating diverse statistical methods to analyse stage-discriminatory cell interactions in colorectal neoplasia*. en. Pages: 2024.06.02.597010 Section: New Results. June 2024. DOI: 10.1101/2024.06.02.597010. URL: https://www.biorxiv.org/content/10.1101/2024.06.02.597010v1 (visited on 11/04/2024).

[S7] Mark M. Fredrickson and Yuguo Chen. "Permutation and randomization tests for network analysis". In: *Social Networks* 59 (Oct. 2019), pp. 171–183. ISSN: 0378-8733. DOI: 10.1016/j.socnet.2019.08.001. URL: https://www.sciencedirect.com/science/article/pii/S0378873318303381 (visited on 11/29/2024).

[S8] Philip J. Clark and Francis C. Evans. "Distance to Nearest Neighbor as a Measure of Spatial Relationships in Populations". en. In: *Ecology* 35.4 (Oct. 1954), pp. 445–453. ISSN: 00129658. DOI: 10.2307/1931034. URL: http://doi.wiley.com/10.2307/1931034 (visited on 11/26/2024).

[S9] Joshua A. Bull et al. "Extended correlation functions for spatial analysis of multiplex imaging data". en. In: *Biological Imaging* (Feb. 2024). DOI: 10.1017/S2633903X24000011. URL: https://doi.org/10.1017/S2633903X24000011 (visited on 12/04/2023).

[S10] Carlo C. Maley et al. "An ecological measure of immune-cancer colocalization as a prognostic factor for breast cancer". en. In: *Breast Cancer Research* 17.1 (Sept. 2015), p. 131. ISSN: 1465-542X. DOI: 10.1186/s13058-015-0638-4. URL: https://doi.org/10.1186/s13058-015-0638-4 (visited on 11/19/2024).

[S11] Muhammad Shaban et al. "A Novel Digital Score for Abundance of Tumour Infiltrating Lymphocytes Predicts Disease Free Survival in Oral Squamous Cell Carcinoma". en. In: *Scientific Reports* 9.1 (Sept. 2019), p. 13341. ISSN: 2045-2322. DOI: 10.1038/s41598-019-49710-z. URL: https://www.nature.com/articles/s41598-019-49710-z (visited on 11/19/2024).

[S12] B. D. Ripley. "Modelling Spatial Patterns". en. In: *Journal of the Royal Statistical Society: Series B (Methodological)* 39.2 (1977). _eprint:

https://onlinelibrary.wiley.com/doi/pdf/10.1111/j.2517-6161.1977.tb01615.x, pp. 172–192. ISSN: 2517-6161. DOI: 10.1111/j.2517-6161.1977.tb01615.x. URL: https://onlinelibrary.wiley.com/doi/abs/10.1111/j.2517-6161.1977.tb01615.x (visited on 02/22/2024).

[S13] H. Edelsbrunner, D. Kirkpatrick, and R. Seidel. "On the shape of a set of points in the plane". In: *IEEE Transactions on Information Theory* 29.4 (July 1983). Conference Name: IEEE Transactions on Information Theory, pp. 551–559. ISSN: 1557-9654. DOI: 10.1109/TIT.1983.1056714. URL: https://ieeexplore.ieee.org/document/1056714 (visited on 03/06/2024).

[S14] Clarence K. Mah et al. "Bento: a toolkit for subcellular analysis of spatial transcriptomics data". In: *Genome Biology* 25.1 (Apr. 2024), p. 82. ISSN: 1474-760X. DOI: 10.1186/s13059-024-03217-7. URL: https://doi.org/10.1186/s13059-024-03217-7 (visited on 08/22/2024).

[S15] Yuzhou Feng et al. "Spatial analysis with SPIAT and spaSim to characterize and simulate tissue microenvironments". en. In: *Nature Communications* 14.1 (May 2023), p. 2697. ISSN: 2041-1723. DOI: 10.1038/s41467-023-37822-0. URL: https://www.nature.com/articles/s41467-023-37822-0 (visited on 03/25/2024).

[S16] Pauli Virtanen et al. "SciPy 1.0: fundamental algorithms for scientific computing in Python". en. In: *Nature Methods* 17.3 (Mar. 2020), pp. 261–272. ISSN: 1548-7105. DOI: 10.1038/s41592-019-0686-2. URL: https://www.nature.com/articles/s41592-019-0686-2 (visited on 05/17/2024).

[S17] S. Kullback and R. A. Leibler. "On Information and Sufficiency". In: *The Annals of Mathematical Statistics* 22.1 (Mar. 1951), pp. 79–86. ISSN: 0003-4851, 2168-8990. DOI: 10.1214/aoms/1177729694. URL: https://projecteuclid.org/journals/annals-of-mathematical-statistics/volume-22/issue-1/On-Information-and-Sufficiency/10.1214/aoms/1177729694.full (visited on 11/19/2024).

[S18] Cédric Villani. "The Wasserstein distances". en. In: *Optimal Transport: Old and New*. Ed. by Cédric Villani. Grundlehren der mathematischen Wissenschaften. Berlin, Heidelberg: Springer, 2009, pp. 93–111. ISBN: 978-3-540-71050-9. DOI: 10.1007/978-3-540-71050-9_6. URL: https://doi.org/10.1007/978-3-540-71050-9_6 (visited on 03/06/2024).

[S19] M. G. Sreelekha, K. Krishnamurthy, and M. V. L. R. Anjaneyulu. "Interaction between Road Network Connectivity and Spatial Pattern". In: *Procedia Technology*. International Conference on Emerging Trends in Engineering, Science and Technology (ICETEST - 2015) 24 (Jan. 2016), pp. 131–139. ISSN: 2212-0173. DOI: 10.1016/j.protcy.2016.05.019. URL: https://www.sciencedirect.com/science/article/pii/S2212017316301025 (visited on 11/29/2024).

[S20] Marc Barthélemy. "Spatial networks". In: *Physics Reports* 499.1 (Feb. 2011), pp. 1–101. ISSN: 0370-1573. DOI: 10.1016/j.physrep.2010.11.002. URL: https://www.sciencedirect.com/science/article/pii/S037015731000308X (visited on 11/29/2024).

[S21] Akrati Saxena and Sudarshan Iyengar. *Centrality Measures in Complex Networks: A Survey*. arXiv:2011.07190. Nov. 2020. DOI: 10.48550/arXiv.2011.07190. URL: http://arxiv.org/abs/2011.07190 (visited on 11/29/2024).

[S22] Caleb R. Stoltzfus et al. "CytoMAP: A Spatial Analysis Toolbox Reveals Features of Myeloid Cell Organization in Lymphoid Tissues". en. In: *Cell Reports* 31.3 (Apr. 2020), p. 107523. ISSN: 22111247. DOI: 10.1016/j.celrep.2020.107523. URL: https://linkinghub.elsevier.com/retrieve/pii/S221112472030423X (visited on 11/27/2023).

[S23] Vincent D. Blondel et al. "Fast unfolding of communities in large networks". en. In: *Journal of Statistical Mechanics: Theory and Experiment* 2008.10 (Oct. 2008), P10008. ISSN: 1742-5468. DOI: 10.1088/1742-5468/2008/10/P10008. URL: https://dx.doi.org/10.1088/1742-5468/2008/10/P10008 (visited on 11/29/2024).

[S24] Zhenqin Wu et al. "Graph deep learning for the characterization of tumour microenvironments from spatial protein profiles in tissue specimens". en. In: *Nature Biomedical Engineering* 6.12 (Dec. 2022), pp. 1435–1448. ISSN: 2157-846X. DOI: 10.1038/s41551-022-00951-w. URL: https://www.nature.com/articles/s41551-022-00951-w (visited on 03/25/2024).

[S25] Ajit J. Nirmal and Peter K. Sorger. *SCIMAP: A Python Toolkit for Integrated Spatial Analysis of Multiplexed Imaging Data*. arXiv:2405.02076 [q-bio]. May 2024. DOI: 10.48550/arXiv.2405.02076. URL: http://arxiv.org/abs/2405.02076 (visited on 05/20/2024).

[S26] Giovanni Palla et al. "Squidpy: a scalable framework for spatial omics analysis". en. In: *Nature Methods* 19.2 (Feb. 2022), pp. 171–178. ISSN: 1548-7105. DOI: 10.1038/s41592-021-01358-2. URL: https://www.nature.com/articles/s41592-021-01358-2 (visited on 05/20/2024).

[S27] Peter Wills and François G. Meyer. "Metrics for graph comparison: A practitioner's guide". en. In: *PLOS ONE* 15.2 (Feb. 2020), e0228728. ISSN: 1932-6203. DOI: 10.1371/journal.pone.0228728. URL: https://journals.plos.org/plosone/article?id=10.1371/journal.pone.0228728 (visited on 11/29/2024).

[S28] Naia Morueta-Holme et al. "A network approach for inferring species associations from co-occurrence data". en. In: *Ecography* 39.12 (2016). _eprint: https://onlinelibrary.wiley.com/doi/pdf/10.1111/ecog.01892, pp. 1139–1150. ISSN: 1600-0587. DOI: 10.1111/ecog.01892. URL: https://onlinelibrary.wiley.com/doi/abs/10.1111/ecog.01892 (visited on 11/27/2023).

[S29] A Baddeley, E Rubak, and R Turner. *Spatial Point Patterns: Methodology and Applications with R*. 1st Edition. Boca Raton, London, New York: CRC Press, Taylor & Francis Group, 2016. URL: https://www.routledge.com/Spatial-Point-Patterns-Methodology-and-Applications-with-R/Baddeley-Rubak-Turner/p/book/9781482210200.

[S30] Eloise Withnell and Maria Secrier. "SpottedPy quantifies relationships between spatial transcriptomic hotspots and uncovers environmental cues of epithelial-mesenchymal plasticity in breast cancer". In: *Genome Biology* 25.1 (Nov. 2024), p. 289. ISSN: 1474-760X. DOI: 10.1186/s13059-024-03428-y. URL: https://doi.org/10.1186/s13059-024-03428-y (visited on 11/25/2024).

[S31] Oscar E. Ospina et al. *spatialGE: A user-friendly web application to democratize spatial transcriptomics analysis*. en. Pages: 2024.06.27.601050 Section: New Results. July 2024. DOI: 10.1101/2024.06.27.601050. URL: https://www.biorxiv.org/content/10.1101/2024.06.27.601050v1 (visited on 08/22/2024).

[S32] Arthur Getis and J. K. Ord. "The Analysis of Spatial Association by Use of Distance Statistics". en. In: *Geographical Analysis* 24.3 (1992). _eprint: https://onlinelibrary.wiley.com/doi/pdf/10.1111/j.1538-4632.1992.tb00261.x, pp. 189–206. ISSN: 1538-4632. DOI: 10.1111/j.1538-4632.1992.tb00261.x. URL: https://onlinelibrary.wiley.com/doi/abs/10.1111/j.1538-4632.1992.tb00261.x (visited on 11/26/2024).

[S33] M. N. M. van Lieshout and A. J. Baddeley. "A nonparametric measure of spatial interaction in point patterns". en. In: *Statistica Neerlandica* 50.3 (1996). _eprint: https://onlinelibrary.wiley.com/doi/pdf/10.1111/j.1467-9574.1996.tb01501.x, pp. 344–361. ISSN: 1467-9574. DOI: 10.1111/j.1467-9574.1996.tb01501.x. URL: https://onlinelibrary.wiley.com/doi/abs/10.1111/j.1467-9574.1996.tb01501.x (visited on 02/29/2024).

[S34] Joshua A. Bull and Helen M. Byrne. "Quantification of spatial and phenotypic heterogeneity in an agent-based model of tumour-macrophage interactions". en. In: *PLOS Computational Biology* 19.3 (Mar. 2023), e1010994. ISSN: 1553-7358. DOI: 10.1371/journal.pcbi.1010994. URL:

https://journals.plos.org/ploscompbiol/article?id=10.1371/journal.pcbi.1010994 (visited on 11/27/2023).

[S35] Ulrich Bauer. "Ripser: efficient computation of Vietoris–Rips persistence barcodes". en. In: *Journal of Applied and Computational Topology* 5.3 (Sept. 2021), pp. 391–423. ISSN: 2367-1734. DOI: 10.1007/s41468-021-00071-5. URL: https://doi.org/10.1007/s41468-021-00071-5 (visited on 03/06/2024).

[S36] Dashti Ali et al. "A Survey of Vectorization Methods in Topological Data Analysis". In: *IEEE Transactions on Pattern Analysis and Machine Intelligence* 45.12 (Dec. 2023). Conference Name: IEEE Transactions on Pattern Analysis and Machine Intelligence, pp. 14069–14080. ISSN: 1939-3539. DOI: 10.1109/TPAMI.2023.3308391. URL: https://ieeexplore.ieee.org/abstract/document/10235748 (visited on 03/06/2024).

[S37] Fabian Pedregosa et al. "Scikit-learn: Machine Learning in Python". en. In: *Journal of Machine Learning Research* 12 (2011), pp. 2825–2830.

[S38] Andrew Butler et al. "Integrating single-cell transcriptomic data across different conditions, technologies, and species". en. In: *Nature Biotechnology* 36.5 (May 2018), pp. 411–420. ISSN: 1546-1696. DOI: 10.1038/nbt.4096. URL: https://www.nature.com/articles/nbt.4096 (visited on 05/07/2026).

[S39] F. Alexander Wolf, Philipp Angerer, and Fabian J. Theis. "SCANPY: large-scale single-cell gene expression data analysis". en. In: *Genome Biology* 19.1 (Feb. 2018), p. 15. ISSN: 1474-760X. DOI: 10.1186/s13059-017-1382-0. URL: https://doi.org/10.1186/s13059-017-1382-0 (visited on 04/30/2026).

[S40] Jiaji G. Chen et al. "Giotto Suite: a multiscale and technology-agnostic spatial multiomics analysis ecosystem". en. In: *Nature Methods* 22.10 (Oct. 2025), pp. 2052–2064. ISSN: 1548-7105. DOI: 10.1038/s41592-025-02817-w. URL: https://www.nature.com/articles/s41592-025-02817-w (visited on 04/30/2026).

[S41] Andrzej Kwinta and Joanna Bac-Bronowicz. "Regular polygons in 2D objects shape description". en. In: *Geomatics, Landmanagement and Landscape* 4 (2020). ISSN: 2300-1496. DOI: 10.15576/GLL/2020.4.43. URL: https://repo.ur.krakow.pl/info/article/UR62ad65e95a3a45efb63ae1d773088563/ (visited on 11/29/2024).

[S42] Chandler D. Gatenbee et al. "Immunosuppressive niche engineering at the onset of human colorectal cancer". en. In: *Nature Communications* 13.1 (Apr. 2022), p. 1798. ISSN: 2041-1723. DOI: 10.1038/s41467-022-29027-8. URL: https://www.nature.com/articles/s41467-022-29027-8 (visited on 01/26/2026).

[S43] Luc Anselin. "Local Indicators of Spatial Association—LISA". en. In: *Geographical Analysis* 27.2 (1995). _eprint: https://onlinelibrary.wiley.com/doi/pdf/10.1111/j.1538-4632.1995.tb00338.x, pp. 93–115. ISSN: 1538-4632. DOI: 10.1111/j.1538-4632.1995.tb00338.x. URL: https://onlinelibrary.wiley.com/doi/abs/10.1111/j.1538-4632.1995.tb00338.x (visited on 11/05/2024).

[S44] Jiaqi Xi and Jonathan Niles-Weed. "Distributional Convergence of the Sliced Wasserstein Process". en. In: *Advances in Neural Information Processing Systems* 35 (Dec. 2022), pp. 13961–13973. URL: https://proceedings.neurips.cc/paper_files/paper/2022/hash/5a5e9197ea547141b4977a5a198bbaac-Abstract-Conference.html (visited on 03/05/2024).

[S45] Nicolas Bonneel et al. "Sliced and Radon Wasserstein Barycenters of Measures". en. In: *Journal of Mathematical Imaging and Vision* 51.1 (Jan. 2015), pp. 22–45. ISSN: 1573-7683. DOI: 10.1007/s10851-014-0506-3. URL: https://doi.org/10.1007/s10851-014-0506-3 (visited on 03/05/2024).

[S46] Wouter Saelens et al. “A comparison of single-cell trajectory inference methods”. en. In: *Nature Biotechnology* 37.5 (May 2019), pp. 547–554. ISSN: 1546-1696. DOI: 10.1038/s41587-019-0071-9. URL: https://www.nature.com/articles/s41587-019-0071-9 (visited on 09/15/2025).
[S47] Laleh Haghverdi et al. “Diffusion pseudotime robustly reconstructs lineage branching”. en. In: *Nature Methods* 13.10 (Oct. 2016), pp. 845–848. ISSN: 1548-7105. DOI: 10.1038/nmeth.3971. URL: https://www.nature.com/articles/nmeth.3971 (visited on 05/05/2026).
