# Supplementary material for: MuSpAn: a toolbox for multiscale spatial analysis
Source: Nat Commun. 2026 Aug 13;17:9737. doi: 10.1038/s41467-026-75649-7 (PMC13562714; doi:10.1038/s41467-026-75649-7)
Supplement: Supplementary file 2 [file 41467_2026_75649_MOESM2_ESM.pdf]

nature portfolio

Corresponding author(s): Joshua A. Bull

Last updated by author(s): Jul 1, 2026

Reporting Summary

Nature Portfolio wishes to improve the reproducibility of the work that we publish. This form provides structure for consistency and transparency in reporting. For further information on Nature Portfolio policies, see our Editorial Policies and the Editorial Policy Checklist.

Statistics

For all statistical analyses, confirm that the following items are present in the figure legend, table legend, main text, or Methods section.

n/a	Confirmed	
☐	☒	The exact sample size (*n*) for each experimental group/condition, given as a discrete number and unit of measurement
☐	☒	A statement on whether measurements were taken from distinct samples or whether the same sample was measured repeatedly
☐	☒	The statistical test(s) used AND whether they are one- or two-sided<br>*Only common tests should be described solely by name; describe more complex techniques in the Methods section.*
☐	☒	A description of all covariates tested
☐	☒	A description of any assumptions or corrections, such as tests of normality and adjustment for multiple comparisons
☐	☒	A full description of the statistical parameters including central tendency (e.g. means) or other basic estimates (e.g. regression coefficient) AND variation (e.g. standard deviation) or associated estimates of uncertainty (e.g. confidence intervals)
☐	☒	For null hypothesis testing, the test statistic (e.g. *F*, *t*, *r*) with confidence intervals, effect sizes, degrees of freedom and *P* value noted<br>*Give P values as exact values whenever suitable.*
☒	☐	For Bayesian analysis, information on the choice of priors and Markov chain Monte Carlo settings
☐	☒	For hierarchical and complex designs, identification of the appropriate level for tests and full reporting of outcomes
☐	☒	Estimates of effect sizes (e.g. Cohen's *d*, Pearson's *r*), indicating how they were calculated

Our web collection on statistics for biologists contains articles on many of the points above.

Software and code

Policy information about availability of computer code

Data collection	All software used is fully described in the manuscript and available at the links provided.
Data analysis	All software used is fully described in the manuscript and available at the links provided. MuSpAn is freely available for non-commercial use via the MuSpAn website (https://www.muspan.co.uk/get-the-code) under an Academic Use Licence. Comprehensive user guides including installation, documentation, and detailed tutorials are available via https://docs.muspan.co.uk. Scripts that reproduce the analysis conducted in this paper can be found at https://github.com/MuSpAn-Multiscale-Spatial-Analysis/Supporting_material_muspan_paper, or https://doi.org/10.5281/zenodo.20933039. A public Github repository for issues tracking, feature suggestion, and community discussion, is available at https://github.com/MuSpAn-Multiscale-Spatial-Analysis/MuSpAn-Public.

For manuscripts utilizing custom algorithms or software that are central to the research but not yet described in published literature, software must be made available to editors and reviewers. We strongly encourage code deposition in a community repository (e.g. GitHub). See the Nature Portfolio guidelines for submitting code & software for further information.

Data

Policy information about availability of data

All manuscripts must include a data availability statement. This statement should provide the following information, where applicable:

- Accession codes, unique identifiers, or web links for publicly available datasets
- A description of any restrictions on data availability
- For clinical datasets or third party data, please ensure that the statement adheres to our policy

The `healthy murine colon' dataset used in Figures 1-5 is available from 10x Genomics (https://www.10xgenomics.com/datasets/fresh-frozen-mouse-colon-with-xenium-multimodal-cell-segmentation-1-standard). MuSpAn domains containing the cell location data used in Figures 2-6 are provided at https://doi.org/10.5281/zenodo.17176282. The synthetic dataset used in Supplementary Fig. 9 is provided within the MuSpAn package, via the command muspan.datasets.load_example_domain(`Synthetic-Points-Architecture').

Research involving human participants, their data, or biological material

Policy information about studies with human participants or human data. See also policy information about sex, gender (identity/presentation), and sexual orientation and race, ethnicity and racism.

Reporting on sex and gender	We have not collected or studied original human data in this manuscript. Examples in the supporting information reproducing results of other pipelines on human data use publicly available datasets, please consult the original manuscripts for their reporting information.
Reporting on race, ethnicity, or other socially relevant groupings	We have not collected or studied original human data in this manuscript. Examples in the supporting information reproducing results of other pipelines on human data use publicly available datasets, please consult the original manuscripts for their reporting information.
Population characteristics	We have not collected or studied original human data in this manuscript. Examples in the supporting information reproducing results of other pipelines on human data use publicly available datasets, please consult the original manuscripts for their reporting information.
Recruitment	We have not collected or studied original human data in this manuscript. Examples in the supporting information reproducing results of other pipelines on human data use publicly available datasets, please consult the original manuscripts for their reporting information.
Ethics oversight	We have not collected or studied original human data in this manuscript. Examples in the supporting information reproducing results of other pipelines on human data use publicly available datasets, please consult the original manuscripts for their reporting information.

Note that full information on the approval of the study protocol must also be provided in the manuscript.

Field-specific reporting

Please select the one below that is the best fit for your research. If you are not sure, read the appropriate sections before making your selection.

☒ Life sciences ☐ Behavioural & social sciences ☐ Ecological, evolutionary & environmental sciences

For a reference copy of the document with all sections, see nature.com/documents/nr-reporting-summary-flat.pdf

Life sciences study design

All studies must disclose on these points even when the disclosure is negative.

Sample size	The sample size used in this study was based on practical considerations. Sample size was determined by availability of suitable AKPT mouse tissues for Xenium spatial transcriptomics and was not based on a formal statistical power calculation. The cohort size of n = 4 biological replicates was considered sufficient to detect meaningful biological effects in this exploratory spatial analysis while minimising animal use.
Data exclusions	No data were excluded from the analyses. All AKPT mouse tissue samples included in Fig. 6 that passed the Xenium quality-control criteria and were analyzed.
Replication	The experiment was performed on tissues from 4 AKPT mice (n = 4 biological replicates). The Xenium spatial transcriptomics analysis was performed once on this cohort.
Randomization	Animals were not randomly allocated to experimental groups because this study analyzed tissues from a predefined AKPT mouse cohort. Grouping was determined by genotype and experimental design, and covariates were not relevant to allocation. During the experimental procedures, randomisation was maintained for the order in which mice were handled and tested, reducing potential biases due to environmental or temporal factors.
Blinding	Blinding was not feasible in this study, as sample preparation required group-specific handling. To minimise bias, we used strict, standardised protocols for all sample processing and data analysis. Investigators were not blinded to group allocation during data collection or analysis, as blinding was not relevant for this descriptive spatial transcriptomics analysis of a single mouse model cohort.

Reporting for specific materials, systems and methods

We require information from authors about some types of materials, experimental systems and methods used in many studies. Here, indicate whether each material, system or method listed is relevant to your study. If you are not sure if a list item applies to your research, read the appropriate section before selecting a response.

Materials & experimental systems

n/a	Involved in the study	
☒	☐	Antibodies
☒	☐	Eukaryotic cell lines
☒	☐	Palaeontology and archaeology
☐	☒	Animals and other organisms
☒	☐	Clinical data
☒	☐	Dual use research of concern
☒	☐	Plants

Methods

n/a	Involved in the study	
☒	☐	ChIP-seq
☒	☐	Flow cytometry
☒	☐	MRI-based neuroimaging

Animals and other research organisms

Policy information about studies involving animals; ARRIVE guidelines recommended for reporting animal research, and Sex and Gender in Research

Laboratory animals	We utilised the Villin-CreERT2;Apcfl/fl;KrasG12D/+;Trp53fl/fl;Tgfβr1fl/fl (n = 2) or Villin-CreERT2;Apcfl/+;KrasG12D/+;Trp53fl/fl;Tgfβr1fl/fl (n = 2) (AKPT) murine model of colorectal cancer. Driver alleles were initiated as follows: A is Apcfl/fl, K is KrasG12D, P is p53fl/fl, T is Tgfβr1fl/fl. This combination recapitulates the genetic and microenvironmental features of aggressive, stromal rich, colorectal cancer. All animals were on C57Bl/6J background.<br><br>Mice were housed under standard institutional conditions under a 13 h light/11 h dark cycle at controlled ambient temperature and humidity, with ad libitum access to food and water.
Wild animals	n/a
Reporting on sex	Both females and males have been used in this study, we did not report sex as a variable in our mouse experiments.
Field-collected samples	No field-collected samples.
Ethics oversight	All procedures were carried out in accordance with Home Office UK regulations (including approval by the local animal welfare ethical review committee, the University of Oxford Animal welfare ethical review board (AWERB)) and the Animals (Scientific Procedures) Act 1986. All mice were housed at the Functional Genetics Facility (Centre for Human Genetics, University of Oxford)

Note that full information on the approval of the study protocol must also be provided in the manuscript.

Plants

Seed stocks	n/a
Novel plant genotypes	n/a
Authentication	n/a
