# Supplementary material for: MuSpAn: a toolbox for multiscale spatial analysis
Source: Nat Commun. 2026 Aug 13;17:9737. doi: 10.1038/s41467-026-75649-7 (PMC13562714; doi:10.1038/s41467-026-75649-7)
Supplement: Supplementary file 3 [file 41467_2026_75649_MOESM3_ESM.pdf]

nature portfolio

Peer Review File

MuSpAn: A Toolbox for Multiscale Spatial Analysis

Corresponding Author: Dr Joshua Bull

This file contains all reviewer reports in order by version, followed by all author rebuttals in order by version.

Version 1:

Reviewer comments:

Reviewer #1

(Remarks to the Author)
This manuscript presents MuSpAn, a Python toolbox for multiscale spatial analysis that unifies spatial statistics and graph-/shape-based operations via a common data model (domain–objects–labels–query). The implementation is broad, and the case studies show that the package can support exploratory analysis in spatial omics and multiplexed imaging. Several central claims would benefit from stronger, more explicit evidence and controls—especially regarding (i) what MuSpAn adds beyond existing toolkits, (ii) robustness/statistical validity of the biological readouts, and (iii) reproducibility across datasets and modalities.
Major comments
The manuscript would be stronger with direct, quantitative comparisons on the same ROIs and preprocessing (same centroids/labels and ROI boundary), showing what MuSpAn enables that a single-scale or existing workflow (e.g., Squidpy, Giotto) would miss. e.g., for Fig. 5B–G, this looks closer to a MuSpAn-specific strength (treating derived regions as first-class objects). Please clarify what additional insight the region-level adjacency test provides beyond a cell-level ME-label adjacency/enrichment analysis available in Squidpy/Giotto.

Provide a short rationale for each scale and include a short tutorial for users on how to choose these parameter values in practice. e.g., ≤30 µm for Delaunay edges (Fig. 5); subcellular transcript localization (<10 µm), cell–cell contact (≈100 µm), tissue structures (≥1000 µm) (Fig. 1B); ~60 µm for neighbourhood composition (Fig. 6I–M); r<20 µm for TCM (Fig. 6H); and ~500 µm for tissue-scale correlations (Fig. 6N–O).

Across the Xenium mouse colon exemplar (Figs. 3–5), the AKPT Xenium CRC analysis (Fig. 6), and the cross-modality case studies (Appendix A; IHC/IMC/CODEX), several conclusions rely on spatial significance testing or z-score–based enrichment (e.g., ANNI, QCM/SES, APT on contact/Delaunay networks, Local Getis–Ord*, cross-PCF/cross-K, and TCM). Because these outputs can depend on the null model, spatial inhomogeneity (including tumour–stroma compartment structure), and boundary effects, the manuscript should state the null hypothesis and randomization scheme for each test (e.g., for APT, whether permutations are global or stratified by tumour vs non-tumour; for Local Getis–Ord*, the spatial weights and whether z-scores are asymptotic or permutation-based, with multiple-testing control), and add basic negative controls such as global and compartment-stratified label permutations and at least one within-compartment (tumour-only vs outside-tumour) reanalysis for a highlighted fibroblast–immune association.

The 87D multiscale feature space and the UMAP/trajectory in Fig. 6P are interpreted as a fibroblast-state–specific interaction continuum aligned with transcriptional progression. It is reasonable that part of this structure reflects spatial compartment differences. However, because several inputs (1-hop contacts, 3-hop neighbourhood composition, PCF-derived summaries, TCM) are expected to vary with tumour vs non-tumour localization and density gradients—and CAF4 is enriched outside the tumour annotation (Fig. 6D–E)—it would be helpful to quantify how much of the “continuum” remains after accounting for compartment effects. For example, the authors could (i) recompute the embedding/trajectory within tumour-only and/or non-tumour-only regions, (ii) adjust for tumour distance / an in-tumour indicator, and/or (iii) use a stratified permutation null that shuffles fibroblast states within compartments to test whether the spatial-feature trajectory–transcriptional alignment exceeds what is expected from compartment enrichment alone.

Some important results in this manuscript depend on segmentation-derived geometries. For example, proximity networks based on cell-boundary distances (Fig. 3F–H; Fig. 6F–G) and the derived 1-hop/3-hop neighbourhoods (Fig. 6I–L) are sensitive to the quality of cell/nucleus segmentation and the resulting geometries. Provide wrappers for widely used segmentation tools (e.g., QuPath, Cellpose, StarDist, DeepCell) that export to MuSpAn objects, if possible. If full

integration/api is tedious, please provide a maintained preprocessing pipeline (scripts/notebooks + documentation) that users can run to obtain the required MuSpAn objects in a reproducible way.

Minor comments
“QuPath” appears as “Qupath” in at least one place.
Recalibrate the wording around “interactions” and “drivers” to avoid over-interpreting spatial compartmentalization–driven associations as evidence of causal cell–cell interactions.
Please report runtime and memory usage as a function of dataset size (e.g., increasing numbers of cells) for the main analysis components.

(Remarks on code availability)
NA

Reviewer #2

(Remarks to the Author)
This manuscript reports a Python package that unifies many existing spatial data analysis tools for application to spatial biology. I think this work is a potentially useful contribution to the field, but not exactly for the reasons stated by the authors. I'd also like to better understand its relationship to existing work.

The authors frame MuSpAn as valuable because it is a single piece of software that unifies existing methods. But this no longer seems like a convincing rationale given that AI agents like Biomni should have little trouble achieving the same thing without requiring the user to even know Python. If this isn't exactly the case right now, it is likely to be the case within a few months.

The authors also argue that MuSpAn is valuable because of what it can do. However, many of the analyses presented in the manuscript seem highly descriptive and it is not clear what interesting biological insights can actually be obtained from the tools that MuSpAn provides. For example, in their murine intestinal neoplasia analysis, the main finding that seems to emerge is that there are four fibroblast subtypes with distinct neighborhoods. In this case, it is not clear, at least to me, what new insights that MuSpAn can provide that could not be obtained with scanpy and squidpy.

It also seems the MuSpAn is missing some key analyses. I saw no discussion of spatial domain detection, differential gene expression analysis, enrichment analysis, cell type deconvolution, etc. I checked the MuSpAn documentation and some of these seem to exist but were not discussed in the paper, and others do not seem to have been implemented. Would a user need to use other packages to do these? If so, then what is the advantage of MuSpAn over other classical spatial analysis packages?

To me, the most interesting section of the paper was the Overview section that described MuSpAn's design philosophy. I actually think this should be emphasized earlier in the paper. This is a useful organizing framework that helps users think about all spatial data that I haven't yet seen explicitly written out elsewhere. I think it's useful because it can help scientists think about what questions to pose of their spatial data. In my opinion this is the primary roadblock to progress at this point, rather than implementation. A discussion of this framework, followed by examples of how to use it, would be great.

(Remarks on code availability)

Version 2:

Reviewer comments:

Reviewer #1

(Remarks to the Author)
I do not have further comments. All my comments were addressed.

(Remarks on code availability)

Reviewer #2

(Remarks to the Author)
The authors have addressed many of my comments but I have a few follow-ups for them to consider.

I am not entirely convinced by their response to my Comment 1. In the response, the authors write, "At present, however, AI tools alone do not replicate the analyses provided by MuSpAn." However, I think AI should be able to implement from scratch the same spatial methods implemented in MuSpAn. Indeed the authors write in the revised text that "Agentic AI approaches to conduct data analysis currently rely on lower-level implementations of code to conduct analyses." I'm not sure why this is a problem. It would be useful to add to Appendix D an example of an analysis that MuSpAn can do that squidpy cannot, then to prompt a language model to implement the same analysis. One prompt I tried using the free version of

ChatGPT was "I have a Xenium dataset in anndata format. Give python code to calculate cross-L functions for two genes. Use the exact transcript locations, not cell locations." I'm curious how the results from the Python code I got would compare to MuSpAn. If the results are similar, more justification of the benefits of MuSpAn would be useful.

In response to my Comment 3 I appreciate the author's more refined characterization of MuSpAn as providing "quantitative metric[s] designed to describe the spatial structure of biological data." I think I know what they mean. However, it seems reasonable to characterize the identification of genes that have different expression levels in different domains (i.e., finding domain marker genes using differential expression) as one such metric, which MuSpAn does not provide. Is there another way to characterize MuSpAn's contribution?

(Remarks on code availability)

Open Access This Peer Review File is licensed under a Creative Commons Attribution 4.0 International License, which permits use, sharing, adaptation, distribution and reproduction in any medium or format, as long as you give appropriate credit to the original author(s) and the source, provide a link to the Creative Commons license, and indicate if changes were made.
In cases where reviewers are anonymous, credit should be given to 'Anonymous Referee' and the source.
The images or other third party material in this Peer Review File are included in the article's Creative Commons license, unless indicated otherwise in a credit line to the material. If material is not included in the article's Creative Commons license and your intended use is not permitted by statutory regulation or exceeds the permitted use, you will need to obtain permission directly from the copyright holder.
To view a copy of this license, visit https://creativecommons.org/licenses/by/4.0/

Reference: Manuscript ID NCOMMS-25-02115A-Z

MuSpAn: A Toolbox for Multiscale Spatial Analysis

Joshua A. Bull, Joshua W. Moore, Shania M. Corry, Muyang Lin, Hayley L. Belnoue-Davis, Eoghan J. Mulholland-Illingworth, Simon J. Leedham and Helen M. Byrne

Response to Reviewers

We thank the reviewers for their helpful comments. We have revised the manuscript in line with their suggestions, and include below a point-by-point response with associated changes to the text. We hope that the revised paper addresses their comments and is now suitable for publication in *Nature Communications*.

Response to Reviewer 1

Preliminary comments. This manuscript presents MuSpAn, a Python toolbox for multiscale spatial analysis that unifies spatial statistics and graph-/shape-based operations via a common data model (domain–objects–labels–query). The implementation is broad, and the case studies show that the package can support exploratory analysis in spatial omics and multiplexed imaging. Several central claims would benefit from stronger, more explicit evidence and controls—especially regarding (i) what MuSpAn adds beyond existing toolkits, (ii) robustness/statistical validity of the biological readouts, and (iii) reproducibility across datasets and modalities.

Comment 1. The manuscript would be stronger with direct, quantitative comparisons on the same ROIs and preprocessing (same centroids/labels and ROI boundary), showing what MuSpAn enables that a single-scale or existing workflow (e.g., Squidpy, Giotto) would miss. e.g., for Fig. 5B–G, this looks closer to a MuSpAn-specific strength (treating derived regions as first-class objects). Please clarify what additional insight the region-level adjacency test provides beyond a cell-level ME-label adjacency/enrichment analysis available in Squidpy/Giotto.

Response: A key difference between MuSpAn and existing packages lies in its design philosophy. MuSpAn enables the flexible combination of multiple methods to interrogate structure in spatial data, including multiscale analyses, such as the region-level adjacency test highlighted in Fig. 5G (see also Appendix A.3).

To clarify how MuSpAn differs from existing tools, we have added a new Supporting Information section (Appendix D) that provides a direct comparison between MuSpAn and other spatial biology packages.

Appendix D also includes a direct quantitative comparison between MuSpAn and SquidPy using a Visium dataset available within SquidPy (chosen as SquidPy accepts only AnnData inputs).

All SquidPy analyses use an underlying graph model that defines cell-cell connectivity. As MuSpAn supports arbitrary network representations, and the implemented quantitative methods overlap, all SquidPy analyses can, in principle, be reproduced within MuSpAn. Therefore we compared a representative subset: visualisation, Ripley's L-function, and Moran's I.

Visualisation results and Moran's I were consistent across packages, whereas substantial discrepancies were observed for Ripley's functions. While we attribute this partially to SquidPy not supporting boundary corrections, analyses on randomly sampled point data show that while accounting for boundary correction terms is important, these effects are insufficient to explain the full discrepancy. We therefore speculate that this may relate to a known issue with SquidPy's handling of multiple cell populations (Appendix D; see also `https://github.com/scverse/squidpy/issues/813`).

Manuscript changes:

- Page 10: "We extended this 'cellular neighbourhood' analysis by converting contiguous regions of cells assigned to the same ME ~~were converted~~ into shapes. This enabled us to conduct an adjacency permutation test (APT) on the derived cell neighbourhood objects which revealed ordered transitions from the serosal wall through to the epithelial cell compartment (Fig. 5E-G). A similar analysis performed directly on the ME labelled cell objects (Fig. 5E) would lead to misleading z-scores strongly affected by cell densities along the borders of the regions."

- Page 15: "Existing tools ~~often specialise in single imaging platforms or narrow questions, leaving researchers without flexible options~~ are often specialised for a single imaging platform or a small set of questions, limiting researchers' flexibility to compare or integrate methods across scales (see the Supplementary Information, Appendix D, for a detailed comparison between MuSpAn and selected spatial biology packages)."

- Pages 50 - 54: A new study (Appendix D) compares MuSpAn with existing packages for spatial imaging data. We summarise key differences between MuSpAn, SquidPy, ScanPy, Seurat, and Giotto Suite, and compare selected quantitative spatial analysis metrics available in both SquidPy and MuSpAn on the same dataset.

Comment 2. Provide a short rationale for each scale and include a short tutorial for users on how to choose these parameter values in practice. e.g., $\leq$ 30µm for Delaunay edges (Fig. 5); subcellular transcript localization ($<$ 10µm), cell–cell contact ($\approx$ 100µm), tissue structures ($\geq$ 1000µm) (Fig. 1B); $\approx$ 60 µm for neighbourhood composition (Fig. 6I–M); $r <$ 20µm for TCM (Fig. 6H); and $\approx$ 500µm for tissue-scale correlations (Fig. 6N–O).

Response: Where specific length scales are chosen, we have added text to explain these choices. The length scales associated with Fig. 1B represent approximate biological scales and are included to emphasise the multiscale nature of spatial biology, rather than as parameters used in spatial analyses.

MuSpAn and its constituent metrics are defined geometrically and are agnostic to spatial scale; as such, scale parameters must reflect the biological length scales of interest to the user. We have added text to clarify this.

Manuscript changes:

- Page 5: "...These include analysis of data across different length scales, calculating statistics within irregularly shaped regions, and the use of multiple complementary methods to support the same biological conclusions. First we consider cell-scale geometry and subcellular transcript localisation, then we progress up the spatial scales to characterise the whole tissue section. The length scales considered are selected to showcase MuSpAn's ability to analyse biological data across spatial scales; any spatial method can be

applied to data at any biological scale, and lengthscale parameters should be motivated by biological, rather than mathematical, considerations. Where appropriate, we explain briefly why specific length scales were chosen for these examples."

- Page 6: "...Lastly, quadrat correlation analysis confirmed positive correlation for SMC-STR1 and exclusion for PROG-COL (Fig. 3I-K), and also identified other, non-random, positive and negative cell associations. Quadrats of size 75µm × 75µm were chosen to ensure that most quadrats would contain around 20-30 cells. Together, these data..."
- Page 6: "...The cross pair correlation function (cross-PCF) identified short to medium range clustering of PROG cells (Fig. 4B, $g > 1$ for $r < 150$µm) but exclusion of PROG-SMCs (Fig. 4B, $g < 1$ for $r < 80$µm), consistent with the discrete compartmentalisation of the epithelium and sub-crypt lamina propria. The cross-PCF was calculated for $r < 250$µm (approximately the width of the epithelium), using annuli of width 15µm (approximately one cell diameter). MuSpAn automatically corrected for boundary effects..."
- Page 10: "We used Delaunay graphs (≤ 30µm, approximating a cell-cell contact network inferred from cell centres) to define cellular neighbourhoods; assigning each cell..."
- Page 10: "Secondly, we compartmentalised the ROI into a hexagonal lattice of side length 100µm (reflecting the approximate width of the tissue structures observed above). Cell counts within..."
- Page 9 (Fig 4 caption): "B: Cross-PCFs for PROG-SMC (blue curve, exclusion) and PROG-PROG (orange curve, colocalisation). Inset, annuli of radii 50µm and 200µm ~~were used to calculate~~show stages in calculating the cross-PCF, with automatic boundary correction (see insets, corresponding to black box in A)."
- Page 10: "...A multiscale workflow (Fig. 6A) was constructed, analysing immune context around cancer-associated fibroblast subtypes (Fig. 6B-E) at three scales: direct cell–cell contact (~~< 10µm~~$\lesssim$ 10µm, Fig. 6F-H), immune neighbourhood composition (~~< 60µm~~$\lesssim$ 60µm, Fig. 6I-M), and pairwise spatial correlations (~~< 500µm~~$\lesssim$ 500µm, Fig. 6N-P)."
- Page 13: "...At the cell–cell contact scale, proximity networks (≤ 1.5µm ; ensuring connections only between cells in direct contact) defined 1-hop fibroblast neighbourhoods (Fig. 6F)."
- Page 13: "Topographical Correlation Maps (TCM, $r < 20$µm; chosen to identify correlation between cells within close proximity) highlighted regional variation in cell interactions..."
- Page 13: "At the neighbourhood scale (≈ 60µm; representing environments within approximately 3 cell diameters), 3-hop immune environments around fibroblasts were clustered by immune cell composition,..."
- Page 13: "At the tissue scale (≈ 500µm; representing a substantial section of each ROI), cross-PCFs confirmed these associations..."

Comment 3. Across the Xenium mouse colon exemplar (Figs. 3–5), the AKPT Xenium CRC analysis (Fig. 6), and the cross-modality case studies (Appendix A; IHC/IMC/CODEX), several conclusions rely on spatial significance testing or z-score–based enrichment (e.g., ANNI, QCM/SES, APT on contact/Delaunay networks, Local Getis–Ord*, cross-PCF/cross-

K, and TCM). Because these outputs can depend on the null model, spatial inhomogeneity (including tumour–stroma compartment structure), and boundary effects, the manuscript should state the null hypothesis and randomization scheme for each test (e.g., for APT, whether permutations are global or stratified by tumour vs non-tumour; for Local Getis–Ord*, the spatial weights and whether z-scores are asymptotic or permutation-based, with multiple-testing control), and add basic negative controls such as global and compartment-stratified label permutations and at least one within-compartment (tumour-only vs outside-tumour) reanalysis for a highlighted fibroblast–immune association.

Response: All MuSpAn metrics requiring a null comparison use models of spatial randomness (via random shuffling or statistical CSR, depending on the metric). MuSpAn compares observed data against spatial randomness to identify differences in spatial correlation relative to this baseline, rather than conducting significance testing against arbitrary null models. This allows deviations from a random baseline to be detected without making positive statements about the nature of any clustering or exclusion (as would arise from testing against a specific statistical process).

Where null hypotheses, boundary corrections, and randomisation schemes are not explicitly described in the main text, details are provided in Appendix E. MuSpAn automatically includes boundary corrections when appropriate, and users can specify analysis boundaries via the `include_boundaries` and `exclude_boundaries` keyword arguments. Additional signposting to this information has been included in the main text.

Compartmentalisation (e.g., tumour vs stroma) is a key design feature in MuSpAn. All analyses can be restricted to spatially relevant compartments via the `include_boundaries` and `exclude_boundaries` keyword arguments (see, e.g., the tutorial *Selecting boundaries for spatial statistics* available at `https://docs.muspan.co.uk/latest/tutorials.html`). We have expanded discussion of compartmentalisation in the main text.

We have also performed a new, within-compartment analysis that extends the fibroblast study in Fig. 6, and shows that the observed correlations are not driven solely by compartmentalisation. These results are presented in Appendix F.4. In addition, Appendix F.5 (see response to Comment 4 below) provides a broader reanalysis of fibroblast spatial-transcriptional alignment while explicitly controlling for tumour compartment effects.

Manuscript changes:

- Page 3: "Over 100 analysis methods are available to interrogate objects within a given region of interest (ROI, shape object), described in detail in the Supplementary Information. ~~The~~ Collections of shapes can be used to interactively restrict analyses to a specific set of ROIs such as manual annotations or tissue compartments. Within these regions, boundary corrections and updates to null models are calculated automatically to ensure spatial quantification methods remain statistically valid. These methods are organised into six modules..."

- Page 5: "Many of the descriptive statistics in these examples use significance testing to identify non-random spatial structure. MuSpAn uses tests against null hypotheses of complete spatial randomness (CSR) to highlight data which exhibit non-random spatial structure; full details of the null models used in this section can be found in the Supplementary Information (Appendix E)."

- Page 20: "Where no boundary is specified, analyses will use the domain boundary. This functionality enables users to assess the impact of compartmentalisation on spatial structure, for instance by comparing the value of within-compartment statistics against those calculated ignoring compartment boundaries. See Appendix A in the SI for examples of spatial analysis within shape boundaries."

- Page 66: Addition of Appendix F.4: Assessing the effect of tumour compartmentalisation on fibroblast–immune spatial associations.
- Page 68: Addition of Appendix F.5: Fibroblast spatial–transcriptional alignment is independent of tumour compartment effects.

Comment 4. The 87D multiscale feature space and the UMAP/trajectory in Fig. 6P are interpreted as a fibroblast-state–specific interaction continuum aligned with transcriptional progression. It is reasonable that part of this structure reflects spatial compartment differences. However, because several inputs (1-hop contacts, 3-hop neighbourhood composition, PCF-derived summaries, TCM) are expected to vary with tumour vs non-tumour localization and density gradients—and CAF4 is enriched outside the tumour annotation (Fig. 6D–E)—it would be helpful to quantify how much of the "continuum" remains after accounting for compartment effects. For example, the authors could (i) recompute the embedding/trajectory within tumour-only and/or non-tumour-only regions, (ii) adjust for tumour distance / an in-tumour indicator, and/or (iii) use a stratified permutation null that shuffles fibroblast states within compartments to test whether the spatial-feature trajectory–transcriptional alignment exceeds what is expected from compartment enrichment alone.

Response: We thank the reviewer for highlighting the possible influence of tumour compartmentalisation on the inferred spatial-feature continuum. We agree that several components of the multiscale feature representation may vary between tumour and non-tumour regions, and that it is, therefore, important to distinguish compartment-driven effects.

We performed additional analyses comparing Fig. 6 with an equivalent study restricted to the tumour compartment (Appendix F.5). We recomputed the embedding and diffusion trajectories for tumour-compartment fibroblasts (Fig. F13C–D). As expected, CAF4 cells were depleted (this population is enriched outside the tumour), but the trajectory structure was preserved and alignment between spatial-feature and transcriptional pseudotime was unchanged (Spearman's $\rho = 0.885$ for all fibroblasts vs. $\rho = 0.882$ for tumour-only fibroblasts). A permutation analysis, randomising fibroblast pseudotime locations, confirms that pseudotime in transcriptional space predicts pseudotime in the spatial-interaction space ($p < 0.001$).

These analyses demonstrate that the fibroblast spatial–transcriptional continuum is not driven by tumour compartmentalisation, but reflects a robust, state-associated organisation of fibroblast–immune interactions. This observation has been clarified in the main text (Results, Fig. 6P).

Manuscript changes:

- Page 14: "...Trajectory inference further supported a continuum of fibroblast–immune ~~interactions~~associations that mirrors transcriptional progression. Notably, this alignment is not driven by tumour compartmentalisation: the trajectory structure and its correspondence with transcriptional state are preserved when the analysis is restricted to tumour-resident fibroblasts only (see Supplementary Information, Appendix F)."
- Page 68: Addition of Appendix F.5: Fibroblast spatial–transcriptional alignment is independent of tumour compartment effects.

Comment 5. Some important results in this manuscript depend on segmentation-derived geometries. For example, proximity networks based on cell-boundary distances (Fig. 3F–H; Fig. 6F–G) and the derived 1-hop/3-hop neighbourhoods (Fig. 6I–L) are sensitive to the quality of cell/nucleus segmentation and the resulting geometries. Provide wrappers for widely used segmentation tools (e.g., QuPath, Cellpose, StarDist, DeepCell) that export to MuSpAn objects, if possible. If full integration/api is tedious, please provide a maintained preprocessing pipeline (scripts/notebooks + documentation) that users can run to obtain the required MuSpAn objects in a reproducible way.

Response: We agree that many spatial analysis methods are sensitive to input geometries. As MuSpAn operates downstream of segmentation pipelines, it can take as input the output of any tool that generates geometric objects (e.g. shapes or points).

MuSpAn supports import of generic shapes and points via its standard `add_data` functions, which require only object coordinates:

- Points - `domain.add_points()`
- Shapes (no holes) - `domain.add_shapes()`
- Shapes (with holes) - `domain.add_shapes_with_internal_holes()`

The MuSpAn website (`https://docs.muspan.co.uk`) provides maintained documentation and tutorials explaining how domains from arbitrary coordinate data. In particular, the first three 'Getting Started' tutorials describe how to add points or shapes from the Python workspace or from .csv files (`https://docs.muspan.co.uk/latest/tutorials.html`).

MuSpAn also provides wrappers to import objects directly from several commonly used segmentation tools and data platforms, with optional loading of associated metadata as labels. While full API integration with all segmentation packages is infeasible, given rapid evolution of these tools, MuSpAn provides API wrappers for QuPath, Xenium Explorer and SpatialData:

- QuPath - `muspan.io.qupath_to_domain()`
- Xenium Explorer - `muspan.io.xenium_to_domain()`
- SpatialData - `muspan.io.spatialdata_to_domain()`

In response to the reviewer's comments, we have added a helper function to import the labelled segmentation masks produced by tools such as DeepCell, CellPose, and StarDist, removing the need for users to convert masks into geometric objects using external packages. This functionality is available from MuSpAn v1.2.5, via:

- Labelled segmentation masks - `muspan.io.segmentation_mask_to_domain()`

As with other MuSpAn functionality, unit tests have been added and complete documentation is available online (`https://docs.muspan.co.uk/latest/generated/muspan.io.segmentation_mask_to_domain.html`). We have also added a tutorial (`https://docs.muspan.co.uk/latest/_collections/importing_data/Importing%20from%20labelled%20tiff.html`) demonstrating this functionality on CellPose segmentation mask, noting that it applies equally to masks generated from, e.g., StarDist or DeepCell.

Manuscript changes:

- Page 3: "It accepts spatial coordinates (e.g., centroids, boundaries) and metadata (e.g., cell types, marker intensities, morphometrics), and is compatible with outputs from any generic segmentation and phenotyping pipelines, or . Additionally, MuSpAn provides helper functions to facilitate data import from frameworks including SpatialData [19,33], QuPath [14], and Xenium Explorer (10x), or from the segmentation masks produced by tools such as CellPose [34], StarDist [35], and CellSAM [36]."

Comment 6. "QuPath" appears as "Qupath" in at least one place.

Response: We thank the reviewer for spotting this typo which has now been corrected.

Manuscript changes:

- Page 16: "Cell or object identification, region annotation, and labelling should be conducted prior to spatial analysis, usingavailable using available pipelines or image analysis software (e.g., Xenium explorer, QuPathQupath, Halo, Deepcell)."

Comment 7. Recalibrate the wording around "interactions" and "drivers" to avoid over-interpreting spatial compartmentalization–driven associations as evidence of causal cell–cell interactions.

Response: We thank the reviewer for highlighting the potential over-interpretation of spatial associations. We have revised the text in the manuscript to avoid causal language (e.g. "drivers" and "'interaction"), more accurately reflecting the descriptive nature of the analyses.

Manuscript changes:

- Page 2: "Advances in imaging technologies now enable these interactionsspatial associations to be mapped at unprecedented resolution...
- Page 2: "Here, we demonstrate the capability of MuSpAn to interrogate cross-cell compartment spatial associationsinteractions across length scales in normal and neoplastic mouse intestine."
- Page 6: "Together, these data highlight MuSpAn's ability to integrate multiple analytical methods to assess proximity based cell interactionscorrelations."
- Page 6: "These tools can be used to rapidly identify significant cell interactionscorrelations across heterogenous cell types within a tissue ecosystem. Applied in this way, MuSpAn acts as a hypothesis generating discovery tool to quantify local tissue cellular organisation and to begin to decipher the signalling networks that underpin key identified proximity-based cell interactionsassociations."
- Page 6: "Having identified some proximity based cell interactionscolocalisation and exclusions, we next used two complementary techniques..."
- Page 10: "...designed to characterise interactionsspatial associations between fibroblasts and immune cell subtypes..."
- Page 10: "Multiscale spatial profiling of fibroblast–immune associationsinteractions in murine intestinal neoplasia"

- Page 10: "Having demonstrated the capacity of MuSpAn to conduct exploratory analyses of multiscale, cross compartment cell ~~interactions~~structure in normal mouse colon tissue, we next used it for focussed analysis of pathological cancer-associated fibroblast-immune cell ~~interactions~~relationships in murine intestinal neoplasia."

- Page 13: "'...Adjacency Permutation Tests (APT) revealed subtype-specific ~~interactions~~ contact co-localisations:..."

- Page 13: "...chosen to identify correlation between cells within close proximity) highlighted regional variation in cell contact co-localisation ~~interactions~~:..."

- Page 14: "...By integrating spatial data from direct contact to tissue-wide co-localisation, ~~MuSpAn uncovers coordinated stromal–immune organisation, reinforcing stromal architecture as a driver of immune evasion and therapy resistance.~~ MuSpAn reveals coordinated stromal–immune organisation across scales, providing a spatial framework consistent with reported links between stromal architecture and immune evasion or therapy resistance."

- Page 14: "Trajectory inference further supported a continuum of fibroblast–immune ~~interactions~~associations that mirrors ~~the~~ transcriptional progression."

- Page 15: "We have highlighted the convergent use of diverse mathematical tools to identify and quantify cell morphology, distribution and cross-compartmental ~~interactions~~spatial associations, and demonstrated the power of this for biological discovery."

**Comment 8.** Please report runtime and memory usage as a function of dataset size (e.g., increasing numbers of cells) for the main analysis components.

Response: We have added a dedicated supplementary section (Appendix G) benchmarking computational runtime across simulated datasets spanning 1,000–100,000 cells for selected analysis components (Fig. G13), together with end-to-end runtimes for the main analyses in the manuscript (Table G3). These results characterise how computational cost scales with dataset size and clarify that MuSpAn is designed to run on minimal hardware (e.g., standard laptops), without requiring HPC resources.

Manuscript changes:

- Page 15: "MuSpAn's user-focussed design permits most statistics to be executed and visualised with a single line of code, and moderately sized datasets can be analysed in seconds on a standard laptop without requiring high performance computing resources (see Supplementary Information, Appendix G). MuSpAn's ~~while its~~ *query* system is intuitive for routine use..."

- Page 71: Addition of Appendix G: Computational runtime considerations.

Response to Reviewer 2

Preliminary comments. This manuscript reports a Python package that unifies many existing spatial data analysis tools for application to spatial biology. I think this work is a potentially useful contribution to the field, but not exactly for the reasons stated by the authors. I'd also like to better understand its relationship to existing work.

Comment 1. The authors frame MuSpAn as valuable because it is a single piece of software that unifies existing methods. But this no longer seems like a convincing rationale given that AI agents like Biomni should have little trouble achieving the same thing without requiring the user to even know Python. If this isn't exactly the case right now, it is likely to be the case within a few months.

Response: We agree that AI promises to play an increasingly important role in spatial analysis, and scientific computing more broadly. At present, however, AI tools alone do not replicate the analyses provided by MuSpAn. For example, the agent mentioned by the reviewer (Biomni) appears to construct analysis pipelines by combining existing packages and libraries, meaning its capabilities can only be aligned with existing tools. The analyses that can be conducted using MuSpAn may serve as useful methodological substrate for agentic AI in the future, with MuSpAn acting as a component of agent-based workflows for spatial analysis.

While it remains unclear which specific statistical methods future agentic AI systems will require, there is currently strong demand from researchers for interactive tools to explore spatial datasets. In particular, the spatial biology community continues to require well-defined methodological frameworks for interrogating specific biological hypotheses (see Appendix B). We have expanded our discussion of AI approaches to reflect this point.

Manuscript changes:

- Page 15: "Beyond exploratory analysis, MuSpAn complements the growing role of AI in spatial biology. While AI excels at pattern recognition, it often lacks interpretability [41]. MuSpAn delivers directly interpretable metrics that provide mechanistic insight (e.g., describing cell–cell contact, exclusion zones, morphology). Metrics can be vectorised for integration into AI workflows, bridging interpretable quantification and predictive modelling (an approach successfully applied in TDA [42, 43]). Agentic AI approaches to conduct data analysis currently rely on lower-level implementations of code to conduct analyses, suggesting that packages to conduct data analysis will continue to be needed if promises of AI-authored data analysis pipelines are fulfilled."

Comment 2. The authors also argue that MuSpAn is valuable because of what it can do. However, many of the analyses presented in the manuscript seem highly descriptive and it is not clear what interesting biological insights can actually be obtained from the tools that MuSpAn provides. For example, in their murine intestinal neoplasia analysis, the main finding that seems to emerge is that there are four fibroblast subtypes with distinct neighborhoods. In this case, it is not clear, at least to me, what new insights that MuSpAn can provide that could not be obtained with scanpy and squidpy

Response:

A key strength of MuSpAn is the provision of *quantitative* yet *interpretable* descriptive metrics. The analyses in Figs. 2-5 demonstrate these tools and how they can be applied. More

broadly, MuSpAn enables qualitative observations of spatial patterning to be translated into quantitative descriptions that support rigorous statistical comparison. This is illustrated in the murine intestinal neoplasia analysis (Fig. 6), where a combination of tools identifies and quantifies differences in the immune landscape surrounding fibroblasts with distinct transcriptional profiles.

MuSpAn does not simply reproduce the functionality of existing tools. We have expanded the Supplementary Information (Appendix D) to clarify differences between MuSpAn and packages used by the spatial biology community. In particular, ScanPy has minimal overlap with MuSpAn; ScanPy is primarily designed for clustering and analysis of single-cell gene expression data rather than quantifying spatial structure.

The closest comparison is with SquidPy, which provides a subset of the spatial analysis methods within MuSpAn. In Appendix D, we present a direct quantitative comparison with MuSpAn on a Visium dataset. MuSpAn provides a general infrastructure which uses abstract geometric objects to enable analysis across arbitrary biological units (e.g., transcripts, cells, or derived spatial features). By contrast, SquidPy is restricted to graph-based representations of spatial data and requires AnnData objects, which limits implementation of spatial statistical approaches. In particular, methods that rely on geometric formulations cannot be directly captured by graph-based models, and so standard spatial statistical considerations such as boundary corrections are not supported (see Appendix D).

Finally, we note that many SquidPy methods are limited to measuring autocorrelation, while MuSpAn additionally supports cross-type spatial analysis within a unified framework.

Manuscript changes:

- Page 3: "Over 100 analysis methods are available to interrogate objects within a given region of interest (ROI, shape object), described in detail in the Supplementary Information. ~~The~~ Collections of shapes can be used to interactively restrict analyses to a specific set of ROIs such as manual annotations or tissue compartments. Within these regions, boundary corrections and updates to null models are calculated automatically to ensure spatial quantification methods remain statistically valid. These methods are organised into six modules..."

- Page 10: "We extended this 'cellular neighbourhood' analysis by converting contiguous regions of cells assigned to the same ME ~~were converted~~ into shapes. This enabled us to conduct an adjacency permutation test (APT) on the derived cell neighbourhood objects which revealed ordered transitions from the serosal wall through to the epithelial cell compartment (Fig. 5E-G). A similar analysis performed directly on the ME labelled cell objects (Fig. 5E) would lead to misleading z-scores strongly affected by cell densities along the borders of the regions."

- Page 15: "Existing tools ~~often specialise in single imaging platforms or narrow questions, leaving researchers without flexible options~~ are often specialised for a single imaging platform or a small set of questions, limiting researchers' flexibility to compare or integrate methods across scales (see the Supplementary Information, Appendix D, for a detailed comparison between MuSpAn and selected spatial biology packages)."

- Page 20: "Where no boundary is specified, analyses will use the domain boundary. This functionality enables users to assess the impact of compartmentalisation on spatial structure, for instance by comparing the value of within-compartment statistics against those calculated ignoring compartment boundaries. See Appendix A in the SI for examples of spatial analysis within shape boundaries."

- Page 50: A new study (Appendix D) has been performed which compares MuSpAn with existing tools commonly used to analyse spatial imaging data. We summarise key differences between MuSpAn, SquidPy, ScanPy, Seurat, and Giotto Suite, and we also quantitatively compare selected quantitative spatial analysis metrics available in both SquidPy and MuSpAn.

> **Comment 3.** It also seems the MuSpAn is missing some key analyses. I saw no discussion of spatial domain detection, differential gene expression analysis, enrichment analysis, cell type deconvolution, etc. I checked the MuSpAn documentation and some of these seem to exist but were not discussed in the paper, and others do not seem to have been implemented. Would a user need to use other packages to do these? If so, then what is the advantage of MuSpAn over other classical spatial analysis packages?

Response:

Can MuSpAn conduct these analyses? MuSpAn is not intended to replace the existing ecosystem of spatial biology tools, but to provide a suite of quantitative descriptive metrics for spatial data after segmentation and labelling. This is illustrated in Fig. 1A, where MuSpAn is positioned downstream of tools which support tasks like cell segmentation (e.g., CellPose) or differential gene expression and cell type deconvolution (e.g., ScanPy and the scVerse).

Consequently, differential gene expression analysis and cell type deconvolution are not implemented in MuSpAn. However, spatial domain detection and enrichment analysis can be performed using MuSpAn, although we have avoided assigning this terminology to these tasks due to the lack of community consensus in their formal definitions. In practice, these analyses can be implemented via `muspan.networks.cluster_neighbourhoods()` and `muspan.helpers.cluster_data()`, allowing both domain detection and neighbourhood enrichment analyses (using clustering methods of their choice from the `scikit-learn` library). We have added tutorial resources to clarify these workflows (see `https://docs.muspan.co.uk/latest/_collections/network_analysis/Network%20methods%20-%202%20-%20community_detection.html`, `https://docs.muspan.co.uk/latest/_collections/network_analysis/Network%20methods%20-%204%20-%20neighbourhood_analysis_continuous.html`, and `https://docs.muspan.co.uk/latest/_collections/workflows/Workflows%20-%203%20-%20Identifying%20spatial%20niches.html`).

Would a user need to use other packages to do these? Yes, MuSpAn is designed to be used alongside other tools. Tasks such as cell type deconvolution and differential gene expression analysis are typically performed using packages like Seurat or ScanPy; since these tools enable cell clustering and annotation rather than interrogating spatial relationships they appear earlier in the analysis pipeline (Fig. 1A). MuSpAn is designed to complement these approaches by enabling downstream quantitative analysis of spatial structure.

What is the advantage of MuSpAn? In this work, we use ‘spatial analysis’ to refer specifically to the quantitative characterisation of spatial organisation (see Fig. 1A); we have clarified this distinction in the manuscript. In this context, packages such as ScanPy and Seurat primarily analyse gene expression data and do not directly quantify spatial structure, whereas MuSpAn focusses on this component. More directly comparable tools include SquidPy and Giotto Suite, which we now discuss in Appendix D. MuSpAn provides a flexible framework based on geometric representations, enabling analyses across arbitrary biological objects (e.g., transcripts, cells, neighbourhoods, or annotated regions). In contrast, other tools are typically based on graph representations which limit the implementation of certain statistical approaches, or require specific input data structures (e.g., AnnData).

MuSpAn supports multiple internal data representations, including graph-based approaches, but also enables continuous-space statistics, probability-based methods, and more general geometric analyses. It also incorporates boundary corrections and flexible domain definitions, which are important components of spatial statistical analysis. These differences are now described in detail in Appendix D.

Manuscript changes:

- Page 3: "MuSpAn is a Python package for spatial analysis, which we here take to encompass any quantitative metric designed to describe the spatial structure of biological data, and which is applicable following segmentation and phenotype assignment (Fig. 1A)."
- Page 15: "Existing tools ~~often specialise in single imaging platforms or narrow questions, leaving researchers without flexible options~~ are often specialised for a single imaging platform or a small set of questions, limiting researchers' flexibility to compare or integrate methods across scales (see the Supplementary Information, Appendix D, for a detailed comparison between MuSpAn and selected spatial biology packages)."
- Page 50: A new study (Appendix D) has been performed which compares MuSpAn with existing tools commonly used to analyse spatial imaging data. In this Appendix, Table D.2 summarises key differences between MuSpAn, SquidPy, ScanPy, Seurat, and Giotto Suite. We also compare selected quantitative spatial analysis metrics available in both SquidPy and MuSpAn (Moran's I, L-functions).

Comment 4. To me, the most interesting section of the paper was the Overview section that described MuSpAn's design philosophy. I actually think this should be emphasized earlier in the paper. This is a useful organizing framework that helps users think about all spatial data that I haven't yet seen explicitly written out elsewhere. I think it's useful because it can help scientists think about what questions to pose of their spatial data. In my opinion this is the primary roadblock to progress at this point, rather than implementation. A discussion of this framework, followed by examples of how to use it, would be great.

Response: We thank the reviewer for highlighting this aspect of MuSpAn. Indeed, a central feature of MuSpAn is its use of geometric representations, which allow analysis of arbitrary objects regardless of their biological interpretation or whether they are derived from other analyses.

Since MuSpAn does not prescribe a specific interpretation of the objects it analyses, it provides an extremely flexible framework for spatial analysis. This contrasts with approaches that focus on particular biological units (e.g., 'single cell'-centric analyses), and enables consistent analysis across a range of spatial scales and derived features.

We have added text to the Introduction and Results sections to emphasise the distinguishing features of our framework, and to better illustrate how it can guide the formulation of biological questions.

Manuscript changes:

- Page 2: "Considerable progress has been made in addressing technical challenges such as storage, visualization, segmentation, and phenotyping, often with the help of Artificial Intelligence-based (AI) tools **bankhead˙qupath˙2017**, **greenwald˙whole-cell˙2022**, **berg˙ilastik˙2019**, **schapiro˙histocat˙2017**, **yuan˙benchmarking˙2024**, **marconato˙spatialdata˙202**

chiu˙napari˙2022, **goldsborough˙instanseg˙2024**. Tools such as DeepCell **marks˙cellsam˙2025**, StarDist **schmidt˙cell˙2018**, and CellPose **stringer˙cellpose˙2021** provide increasingly robust cell segmentation, while packages including ScanPy **wolf˙scanpy˙2018** and Seurat **hao˙dictionary˙2024** work with spatial data to assign phenotypes to distinct cells. However, after applying these tools~~However,~~ a central challenge remains: extracting meaningful biological insight from spatially complex data. Averaged metrics such as cell counts or densities obscure patterns of cellular positioning that often hold the key to biological function. For example, the prognostic significance of immune cells in cancer differs dramatically depending on whether they cluster at the tumour margin or infiltrate the lesion **bruni˙immune˙2020**. There is a critical need for tools providing interpretable quantitative metrics designed to describe the spatial structure and organisation of biological data, a process we will refer to as spatial analysis."

- Page 2: "...Few platforms support systematic comparison and integration of diverse methods; notably SquidPy provides a range of graph-based approaches with efficient data structures **palla˙squidpy˙2022**, **virshup˙scverse˙2023** and Giotto Suite **chen˙giotto˙2025** includes an end-to-end ecosystem containing several spatial analysis methods. However, opportunities~~. Opportunities~~ to combine complementary mathematical approaches ~~therefore~~ remain underexploited, despite improving feature detection **bull˙integrating˙2024**. In response to this, we present a new platform, MuSpAn, which extends the work of previous toolboxes by exploiting the generic mathematical structures underlying spatial data to empower the user to conduct generic spatial analyses on cells, cell-derived objects such as neighbourhoods, or indeed any spatial objects including manual annotations, tissue boundaries, transcripts, and more."

- Page 3: "MuSpAn is a Python package for spatial analysis, which we here take to encompass any quantitative metric designed to describe the spatial structure of biological data and which is applicable following segmentation and phenotype assignment (Fig. 1A)."

- Page 3: "The key design principle underlying MuSpAn is that quantitative descriptions of spatial structure can be applied to any geometric objects, regardless of their biological interpretation. Intuitively, we understand that a polygon has an area which can be measured, regardless of whether the polygon represents the computationally detected boundary of a cell, a manually drawn annotation, or the boundary of a necrotic region of tissue on a digital pathology slide. MuSpAn provides a vast library of quantitative descriptive tools for working with arbitrary geometric data, based on mathematical methods developed in fields as diverse as astronomy, ecology, and statistics. In spatial biology, we can derive second-order objects from primary objects of biological interest: a collection of transcripts in Xenium data can be viewed as belonging to a single cell, clusters of cells with similar profiles may be treated as forming a single emergent tissue unit (e.g., a colorectal crypt), and these can in turn be viewed as geometric objects that form part of a larger emergent structure. By providing flexible tools to interrogate the spatial arrangement of these structures that divorce their geometric interpretation from their biological one, the key strength of MuSpAn is its capacity to move easily between different biological spatial scales, and to chain together complex spatial analyses of derived objects into flexible computational pipelines."

Oxford Mathematics

Andrew Wiles Building
Radcliffe Observatory Quarter
Woodstock Road
Oxford OX2 6GG
United Kingdom

+44 (0)1865 273525
enquiries@maths.ox.ac.uk
www.maths.ox.ac.uk

UNIVERSITY OF OXFORD
Mathematical Institute

Response to Further Reviewer Comments

We thank the reviewer for their further helpful comments. We have revised the manuscript in line with their suggestions, and include below a point-by-point response. We hope that the revised paper addresses their comments and is now suitable for publication in *Nature Communications*.

Comment 1:

I am not entirely convinced by their response to my Comment 1. In the response, the authors write, "At present, however, AI tools alone do not replicate the analyses provided by MuSpAn." However, I think AI should be able to implement from scratch the same spatial methods implemented in MuSpAn. Indeed the authors write in the revised text that "Agentic AI approaches to conduct data analysis currently rely on lower-level implementations of code to conduct analyses." I'm not sure why this is a problem. It would be useful to add to Appendix D an example of an analysis that MuSpAn can do that squidpy cannot, then to prompt a language model to implement the same analysis. One prompt I tried using the free version of ChatGPT was "I have a Xenium dataset in anndata format. Give python code to calculate cross-L functions for two genes. Use the exact transcript locations, not cell locations." I'm curious how the results from the Python code I got would compare to MuSpAn. If the results are similar, more justification of the benefits of MuSpAn would be useful.

We agree that, in principle, any sufficiently capable AI system that can write arbitrary code could reproduce the computational steps implemented in MuSpAn. At present, this does not appear to be reliably achievable from a single prompt. Using the reviewer's suggested prompt, we obtained non-functioning code: the model assumed an incorrect structure for the *anndata* object, relied on an unpublished external library (*pointpats*) for the L-function, and omitted essential components such as boundary corrections. The model also provided no indication that these omissions would materially affect the results, which is precisely the type of issue MuSpAn is designed to handle robustly and transparently.

With sufficient iterative prompting and user expertise, we agree that one could guide an AI system to produce code that replicates MuSpAn's functionality. However, this would require substantial additional work beyond simply calling an L-function implementation, and would not resemble the experience of using a stable, well-tested, domain-specific software package. As noted in our initial response, an AI model trained on MuSpAn itself would likely be able to conduct these analyses more directly by using MuSpAn as a substrate.

We also respectfully note the parallel between the reviewer's observation that "AI should be able to implement from scratch the same spatial methods implemented in MuSpAn" and the analogous statement that "any competent programmer should be able to implement from scratch the same spatial methods implemented in MuSpAn." Both are true, but neither diminishes the value of providing a rigorously developed, validated, and user-friendly software package for the community.

In response to the reviewer's comment, we have made the following alterations to the text:

"~~Agentic AI approaches to conduct data analysis currently rely on lower-level implementations of code to conduct analyses, suggesting that packages to conduct data analysis will continue to be needed if promises of AI-authored data analysis pipelines are fulfilled.~~ Agentic AI currently relies on lower-level implementations of code to conduct data analyses, indicating that dedicated analytical packages will remain important even as AI-authored pipelines evolve. Although these systems may increasingly automate spatial analyses, reliable biological interpretation will still depend on domain-specific tools that provide robust and transparent quantification."

Comment 2:

In response to my Comment 3 I appreciate the author's more refined characterization of MuSpAn as providing "quantitative metric[s] designed to describe the spatial structure of biological data." I think I know what they mean. However, it seems reasonable to characterize the identification of genes that have different expression levels in different domains (i.e., finding domain marker genes using differential expression) as one such metric, which MuSpAn does not provide. Is there another way to characterize MuSpAn's contribution?

We agree that MuSpAn cannot provide every possible quantitative metric for characterising spatial structure. Our description of MuSpAn as a package providing quantitative metrics designed to describe spatial structure is

Oxford Mathematics

Andrew Wiles Building
Radcliffe Observatory Quarter
Woodstock Road
Oxford OX2 6GG
United Kingdom

+44 (0)1865 273525
enquiries@maths.ox.ac.uk
www.maths.ox.ac.uk

University of Oxford
Mathematical Institute

intended to convey its scope, without implying exhaustiveness. It is unclear what alternative phrasing would be more accurate, and adding qualifiers such as “many” or “a wide range of” throughout would not materially improve clarity.

Regarding differential expression, we appreciate the reviewer’s clarification. As noted previously, terminology in spatial biology is not always used consistently across the field. If differential expression is taken to refer to identifying genes enriched or depleted across spatial domains, rather than its more common single-cell usage, MuSpAn can indeed support this: users may assign labels to spatial regions and compute summary metrics (including gene expression) for objects within those regions. As illustrated in Figs. 2 and 5, these region-level summaries can then be used for downstream comparisons or analyses. In this sense, MuSpAn can certainly be used to identify spatially enriched gene signatures, and even conduct further spatial analyses based on this (e.g., quantifying spatial patterning in the locations of domains with high or low gene expression for some gene).

Our prior comments were designed to make clear that MuSpAn does not implement a full differential-expression workflow in the traditional single-cell transcriptomics / RNA-seq sense.
